# Supplementary material for: A Resident Narrative Medicine Curriculum to Promote Professional Identity Development: Story-Based Sessions Grounded in Narrative Learning Theory
Source: MedEdPORTAL. 2024 Oct 22;20:11446. doi: 10.15766/mep_2374-8265.11446 (PMC11493853; doi:10.15766/mep_2374-8265.11446)
Supplement: Supplementary file 1 — Facilitator Guide.docxBurnout and Moral Injury.pptxCompassion Fatigue.pptxWorking Through a Pandemic.pptxDifficult Patient.pptxThe New Normal.pptxFinding Meaning.pptxUnpublished Narratives.docxSurvey.docx [file mep_2374-8265.11446-s001.zip › E. Difficult Patient.pptx]

## Slide 1
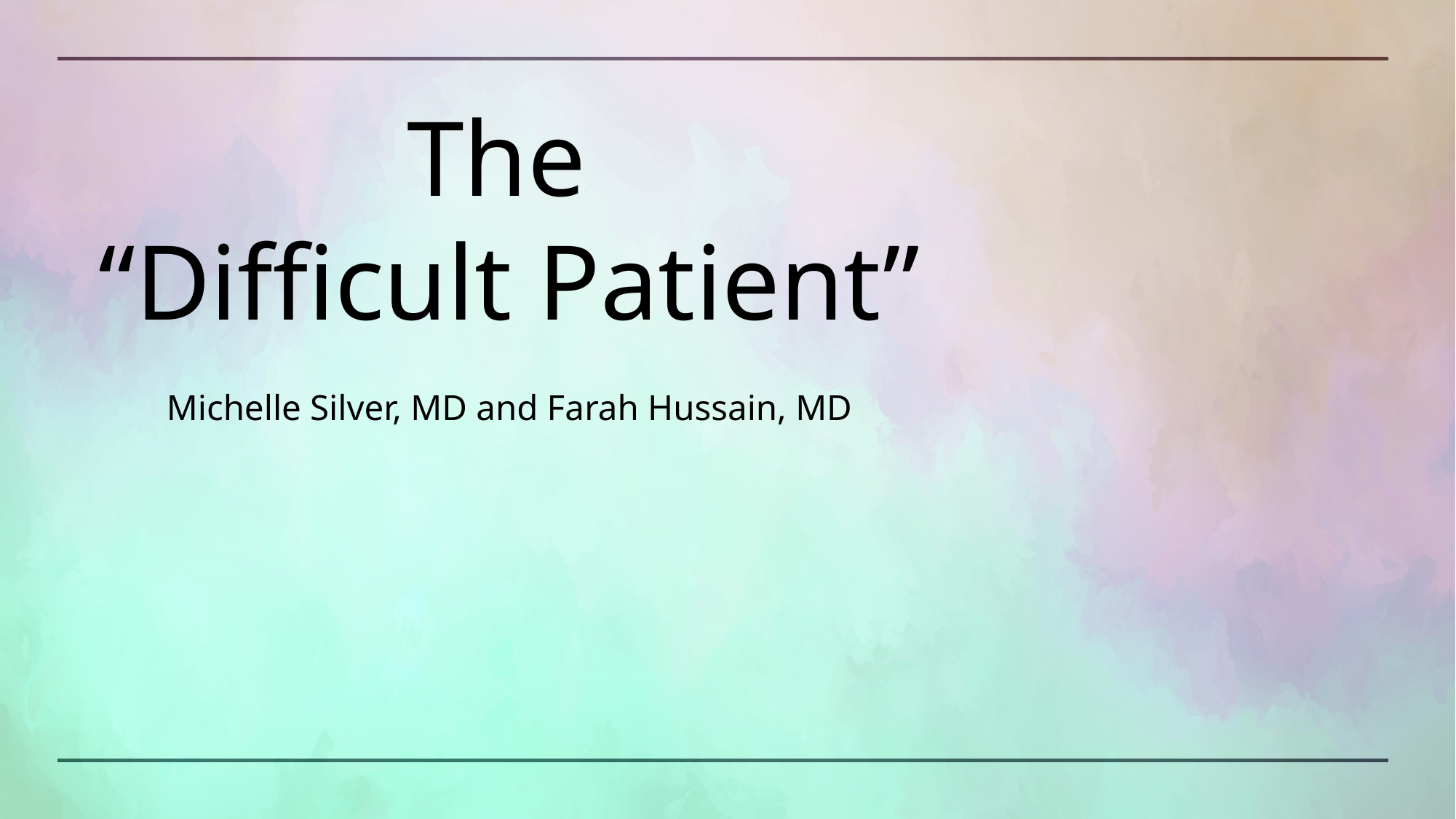

# The “Difficult Patient”
Michelle Silver, MD and Farah Hussain, MD

## Slide 2
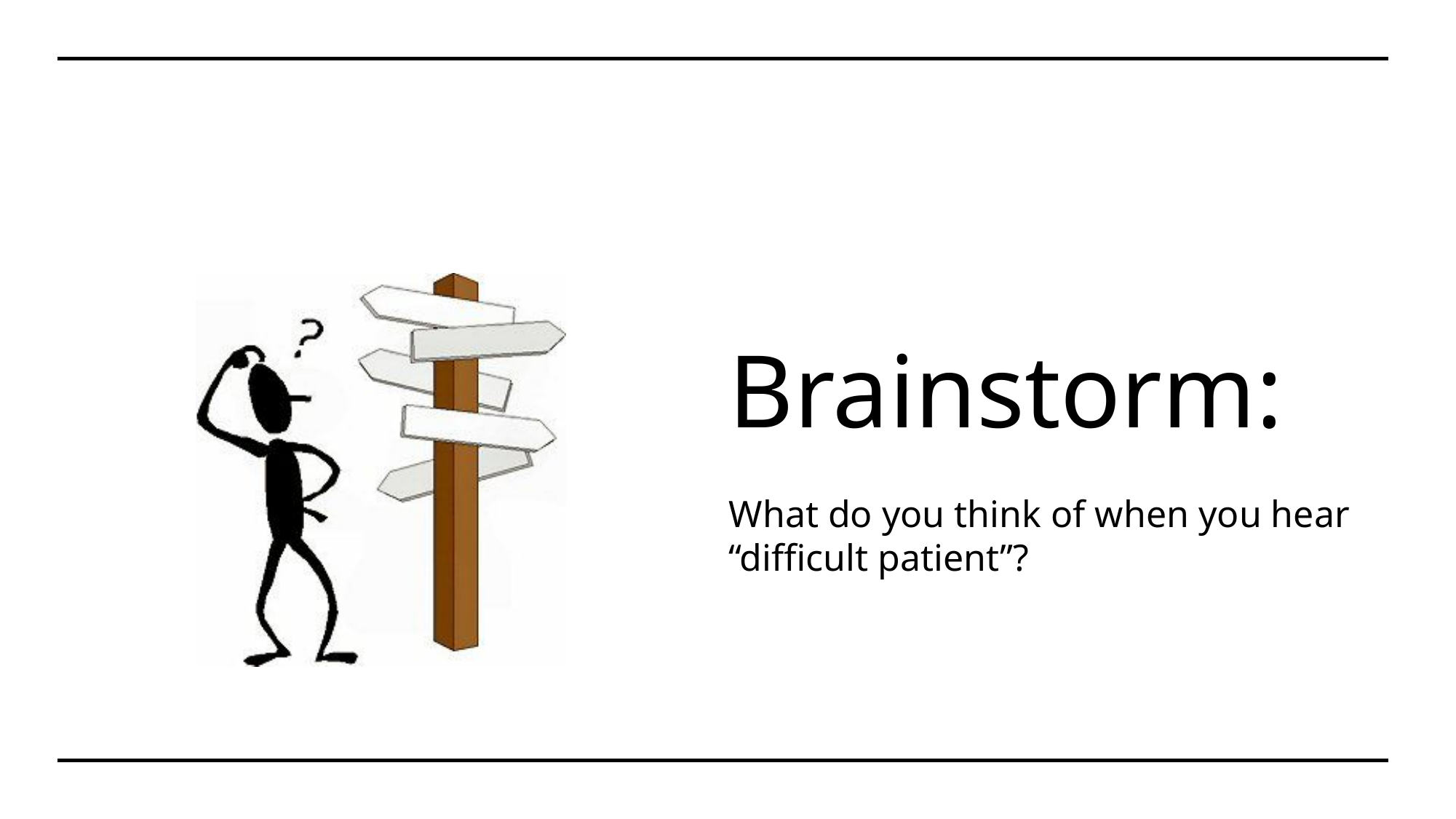

# Brainstorm:
What do you think of when you hear “difficult patient”?

## Slide 3
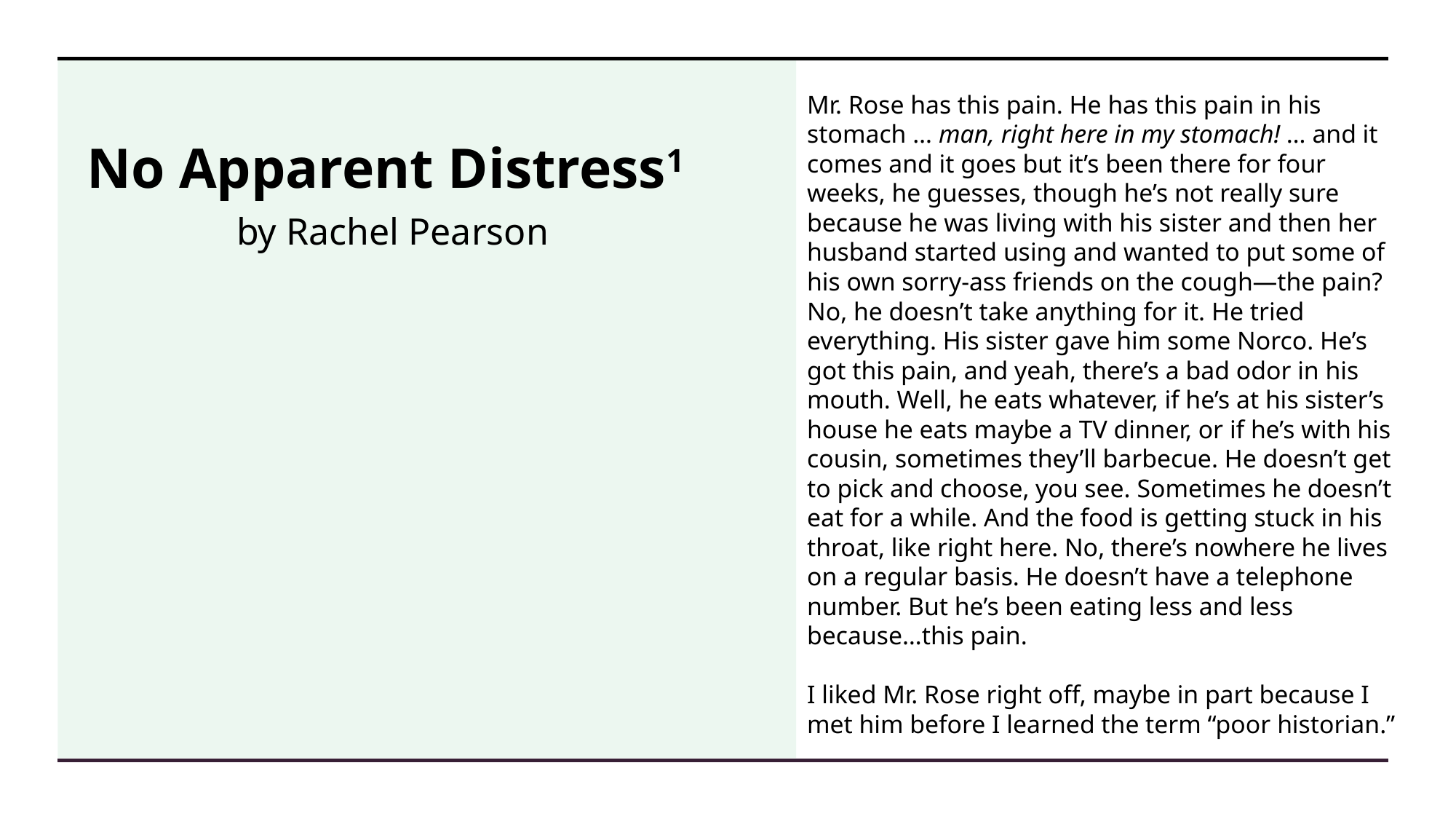

Mr. Rose has this pain. He has this pain in his stomach … man, right here in my stomach! … and it comes and it goes but it’s been there for four weeks, he guesses, though he’s not really sure because he was living with his sister and then her husband started using and wanted to put some of his own sorry-ass friends on the cough—the pain? No, he doesn’t take anything for it. He tried everything. His sister gave him some Norco. He’s got this pain, and yeah, there’s a bad odor in his mouth. Well, he eats whatever, if he’s at his sister’s house he eats maybe a TV dinner, or if he’s with his cousin, sometimes they’ll barbecue. He doesn’t get to pick and choose, you see. Sometimes he doesn’t eat for a while. And the food is getting stuck in his throat, like right here. No, there’s nowhere he lives on a regular basis. He doesn’t have a telephone number. But he’s been eating less and less because…this pain.
I liked Mr. Rose right off, maybe in part because I met him before I learned the term “poor historian.”
No Apparent Distress1
by Rachel Pearson

## Slide 4
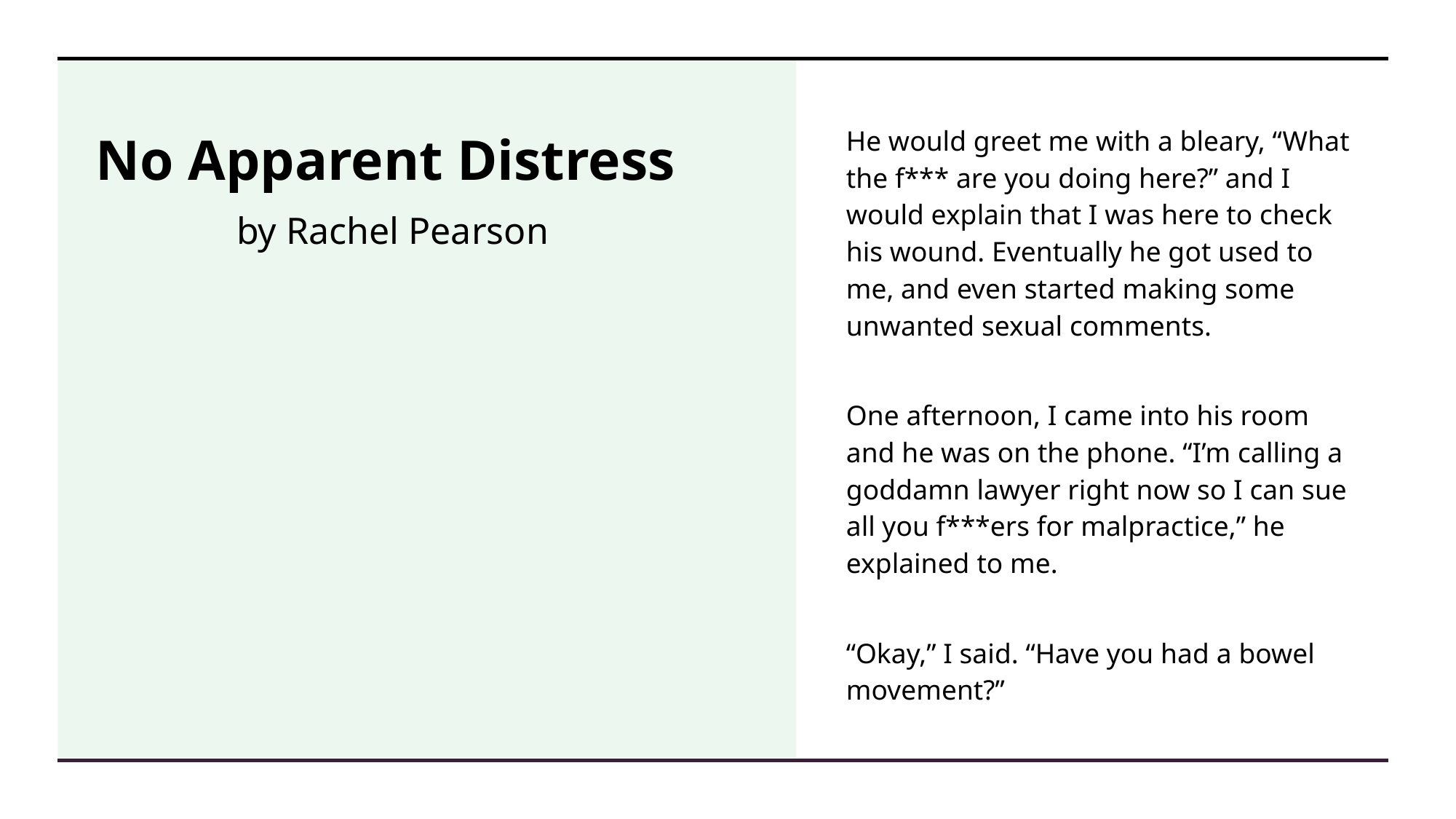

No Apparent Distress
by Rachel Pearson
He would greet me with a bleary, “What the f*** are you doing here?” and I would explain that I was here to check his wound. Eventually he got used to me, and even started making some unwanted sexual comments.
One afternoon, I came into his room and he was on the phone. “I’m calling a goddamn lawyer right now so I can sue all you f***ers for malpractice,” he explained to me.
“Okay,” I said. “Have you had a bowel movement?”

## Slide 5
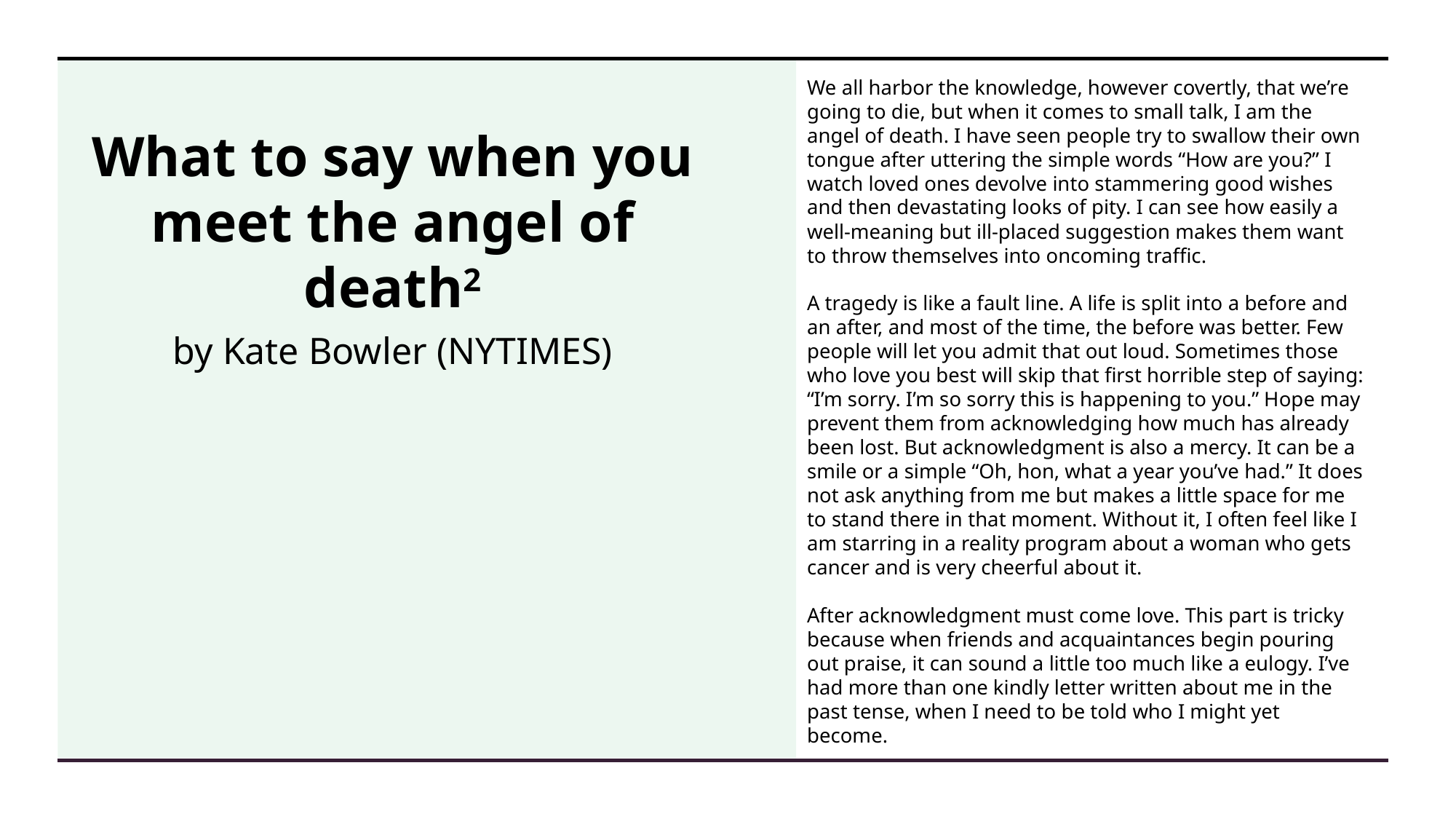

We all harbor the knowledge, however covertly, that we’re going to die, but when it comes to small talk, I am the angel of death. I have seen people try to swallow their own tongue after uttering the simple words “How are you?” I watch loved ones devolve into stammering good wishes and then devastating looks of pity. I can see how easily a well-meaning but ill-placed suggestion makes them want to throw themselves into oncoming traffic.
A tragedy is like a fault line. A life is split into a before and an after, and most of the time, the before was better. Few people will let you admit that out loud. Sometimes those who love you best will skip that first horrible step of saying: “I’m sorry. I’m so sorry this is happening to you.” Hope may prevent them from acknowledging how much has already been lost. But acknowledgment is also a mercy. It can be a smile or a simple “Oh, hon, what a year you’ve had.” It does not ask anything from me but makes a little space for me to stand there in that moment. Without it, I often feel like I am starring in a reality program about a woman who gets cancer and is very cheerful about it.
After acknowledgment must come love. This part is tricky because when friends and acquaintances begin pouring out praise, it can sound a little too much like a eulogy. I’ve had more than one kindly letter written about me in the past tense, when I need to be told who I might yet become.
What to say when you meet the angel of death2
by Kate Bowler (NYTIMES)

## Slide 6
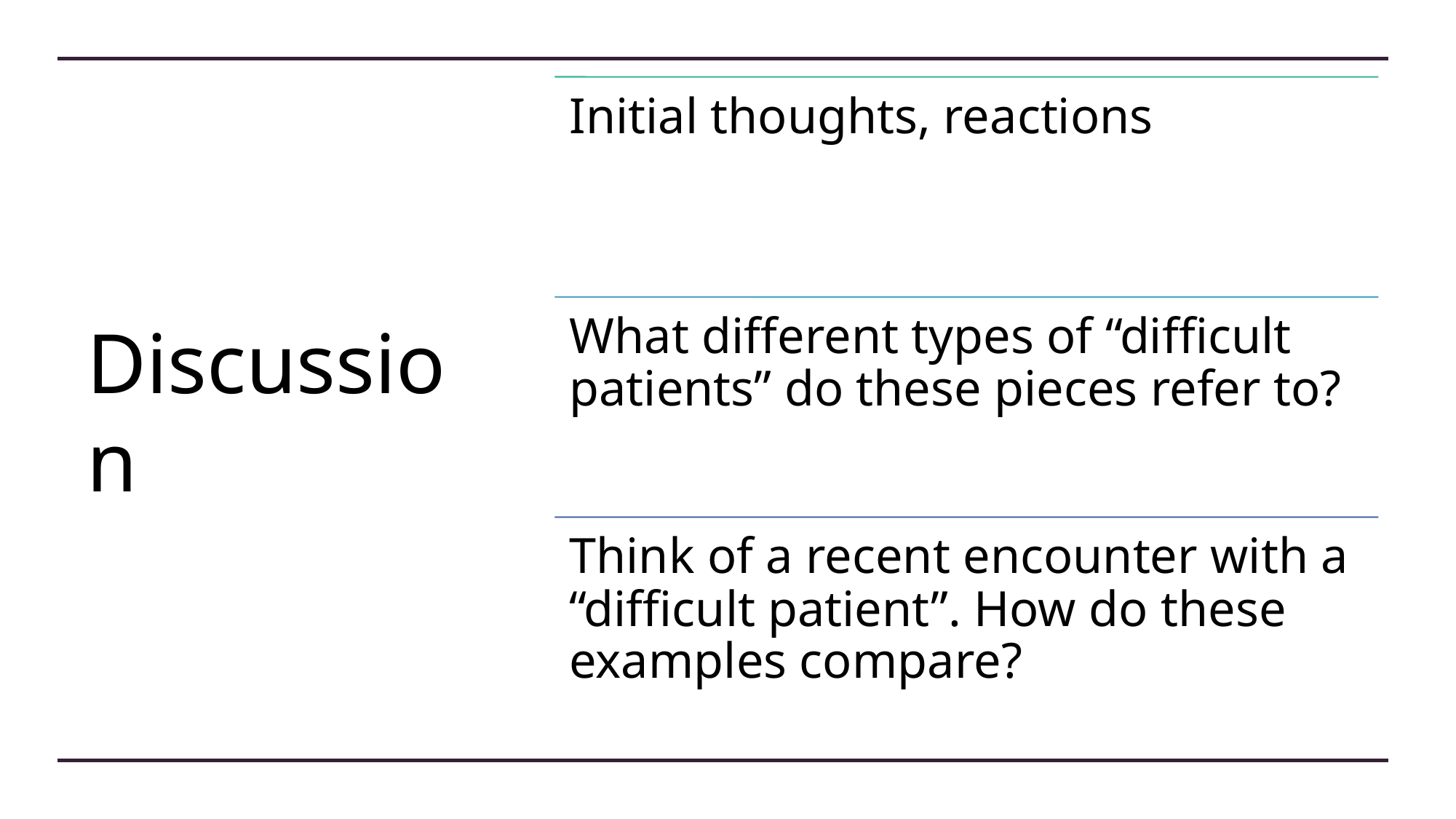

# Discussion

## Slide 7
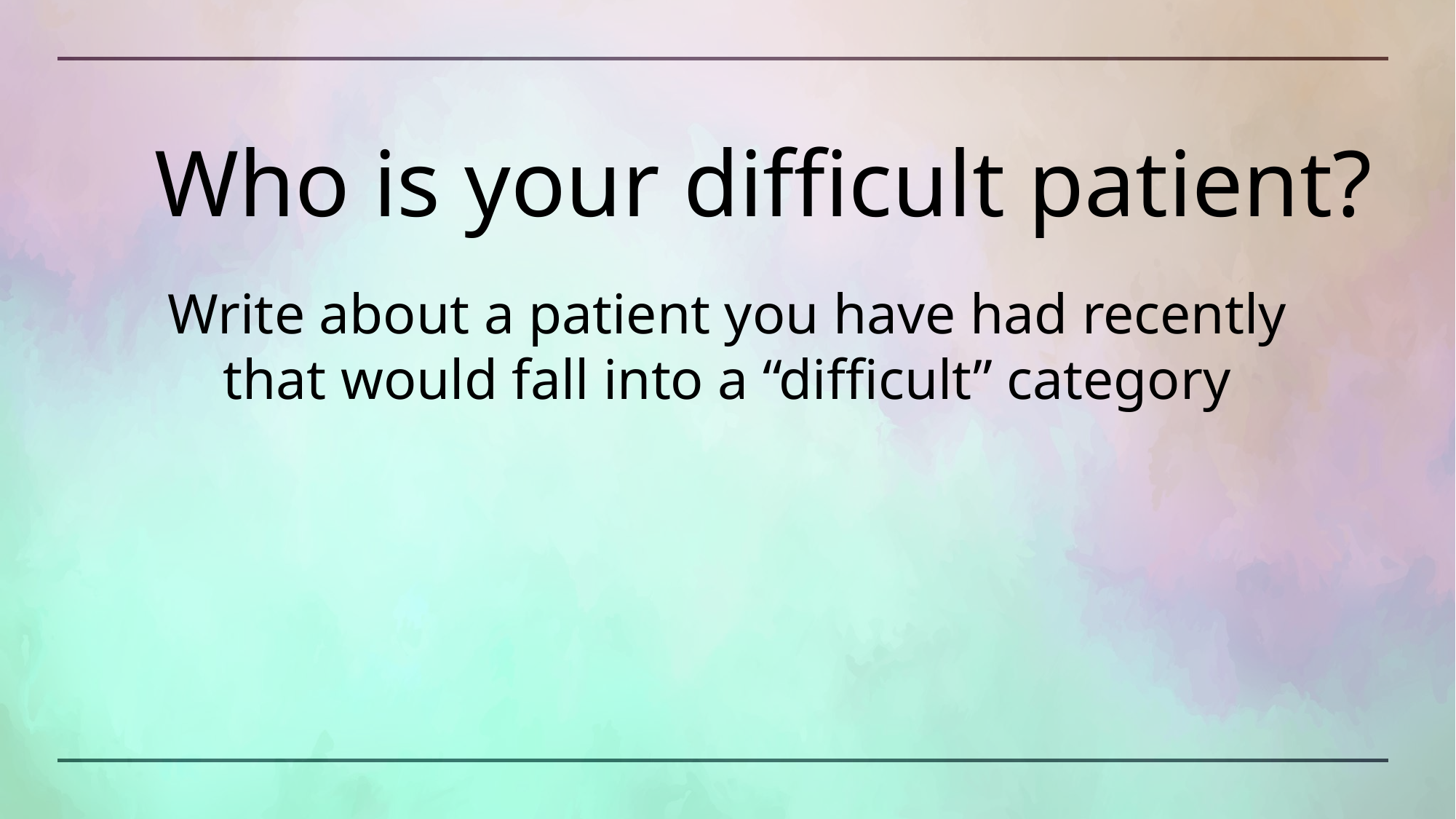

Who is your difficult patient?
Write about a patient you have had recently that would fall into a “difficult” category

## Slide 8
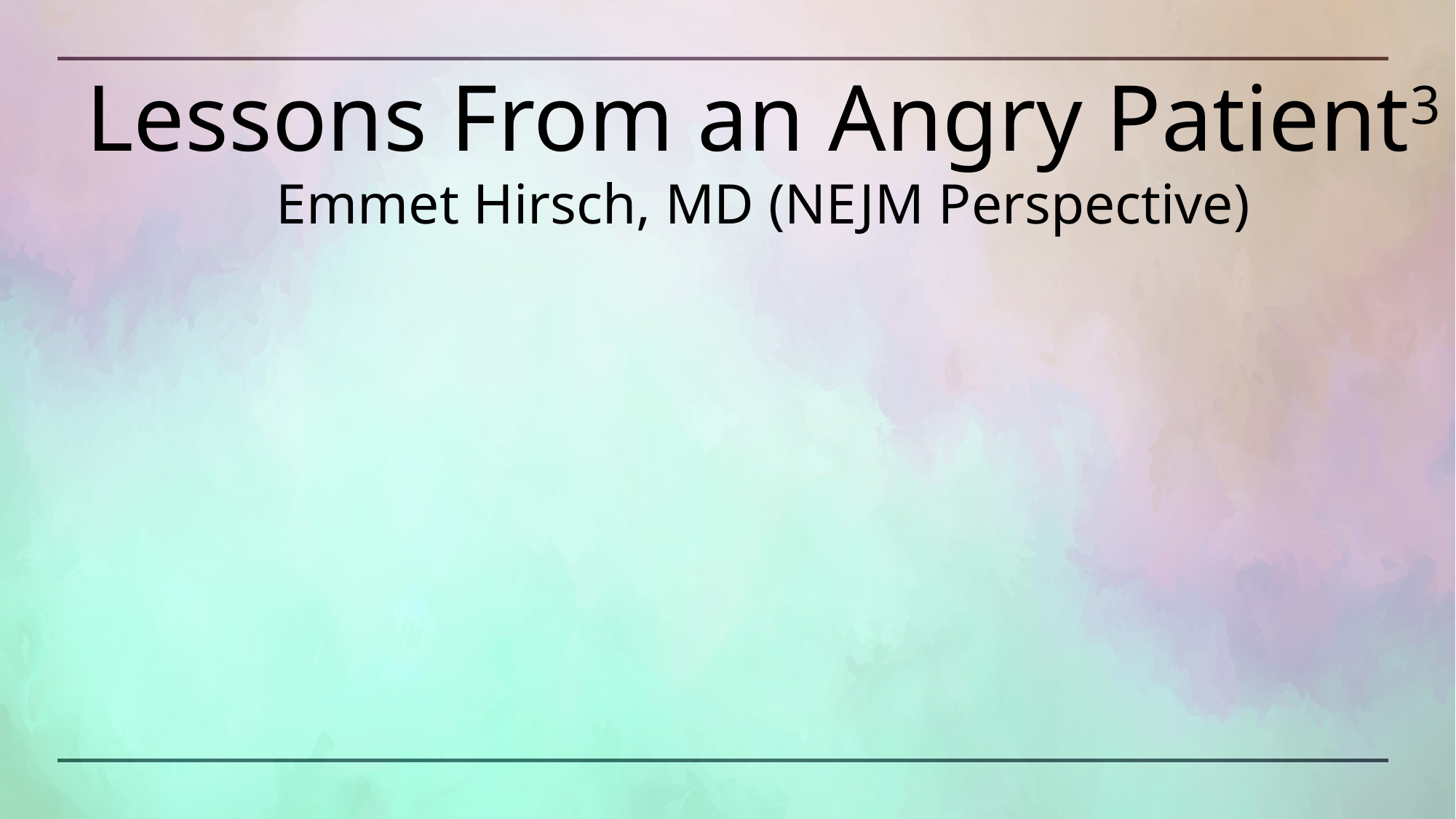

Lessons From an Angry Patient3
Emmet Hirsch, MD (NEJM Perspective)

## Slide 9
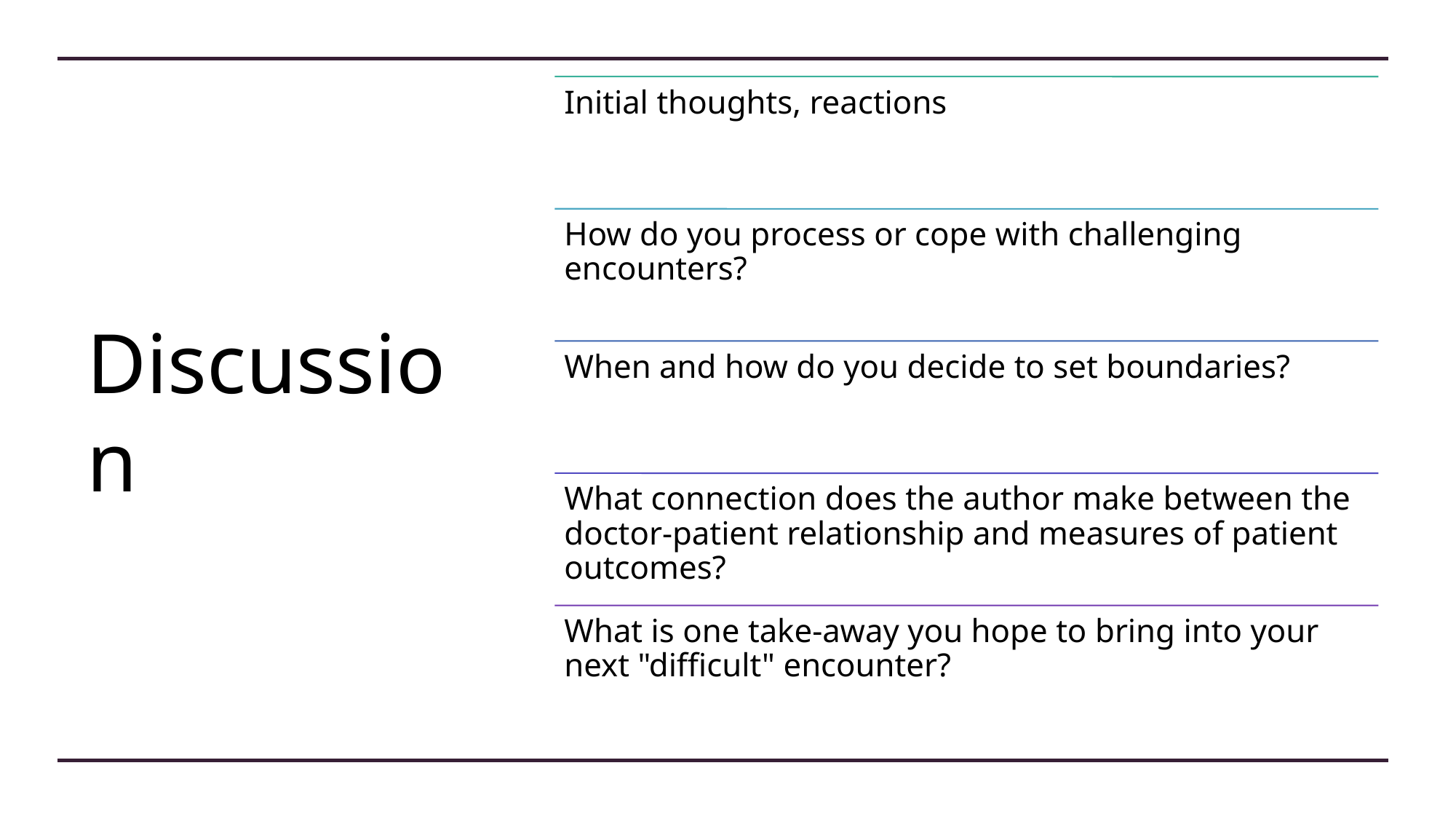

# Discussion

## Slide 10
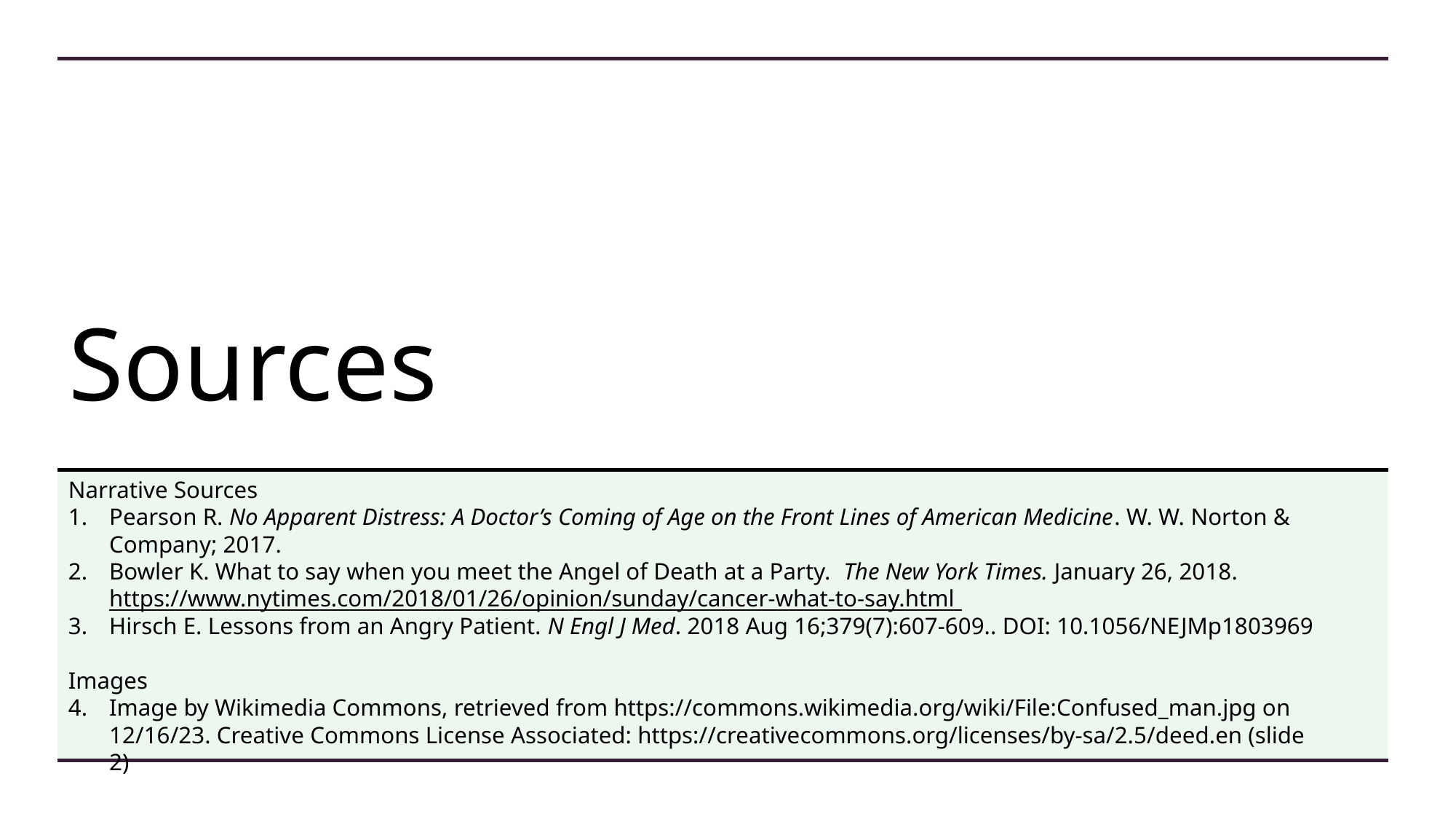

# Sources
Narrative Sources
Pearson R. No Apparent Distress: A Doctor’s Coming of Age on the Front Lines of American Medicine. W. W. Norton & Company; 2017.
Bowler K. What to say when you meet the Angel of Death at a Party. The New York Times. January 26, 2018. https://www.nytimes.com/2018/01/26/opinion/sunday/cancer-what-to-say.html
Hirsch E. Lessons from an Angry Patient. N Engl J Med. 2018 Aug 16;379(7):607-609.. DOI: 10.1056/NEJMp1803969
Images
Image by Wikimedia Commons, retrieved from https://commons.wikimedia.org/wiki/File:Confused_man.jpg on 12/16/23. Creative Commons License Associated: https://creativecommons.org/licenses/by-sa/2.5/deed.en (slide 2)

## Slide 11
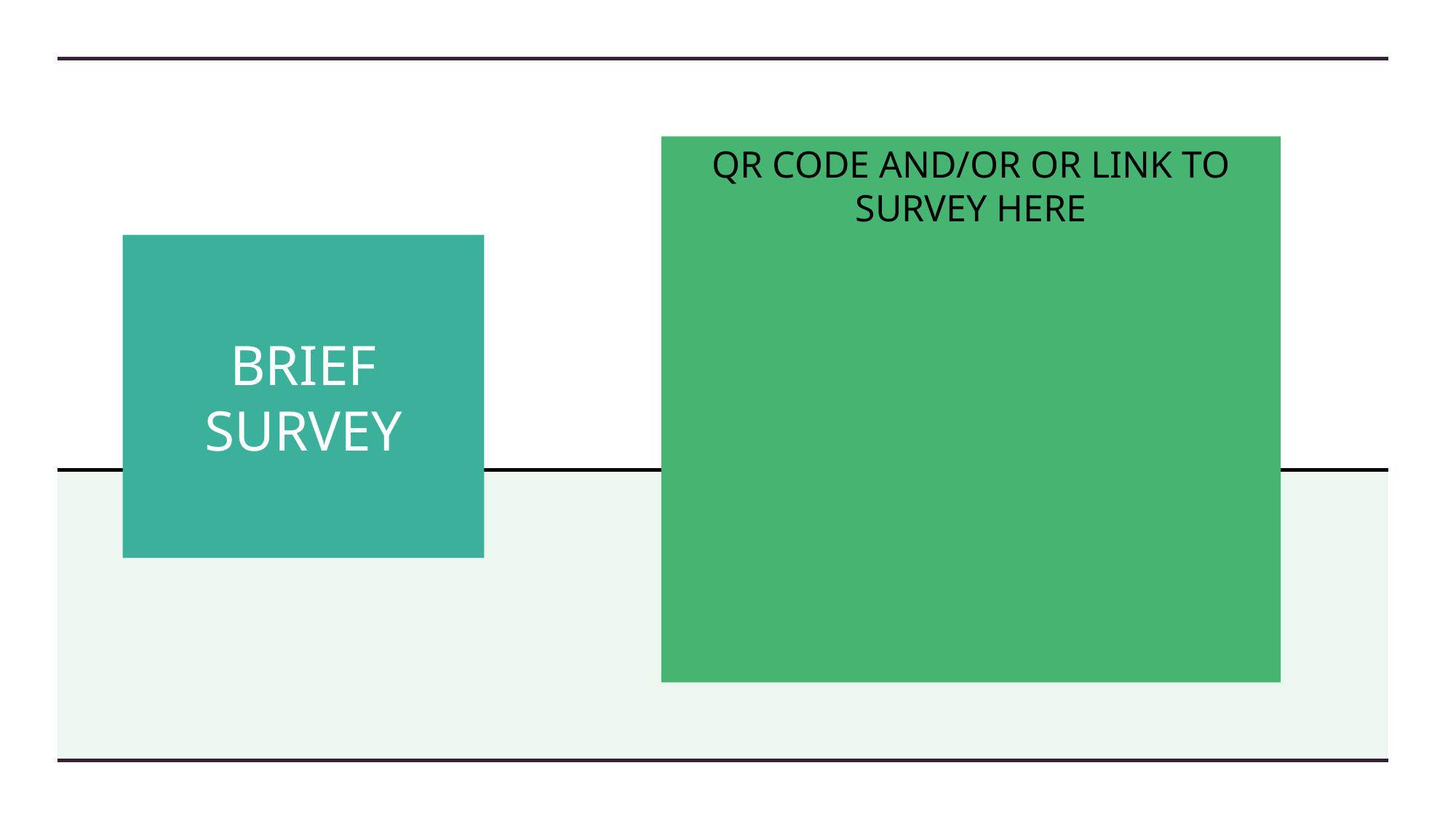

QR CODE AND/OR OR LINK TO SURVEY HERE
# BRIEF SURVEY
