# Supplementary material for: A Resident Narrative Medicine Curriculum to Promote Professional Identity Development: Story-Based Sessions Grounded in Narrative Learning Theory
Source: MedEdPORTAL. 2024 Oct 22;20:11446. doi: 10.15766/mep_2374-8265.11446 (PMC11493853; doi:10.15766/mep_2374-8265.11446)
Supplement: Supplementary file 1 — Facilitator Guide.docxBurnout and Moral Injury.pptxCompassion Fatigue.pptxWorking Through a Pandemic.pptxDifficult Patient.pptxThe New Normal.pptxFinding Meaning.pptxUnpublished Narratives.docxSurvey.docx [file mep_2374-8265.11446-s001.zip › F. The New Normal.pptx]

## Slide 1
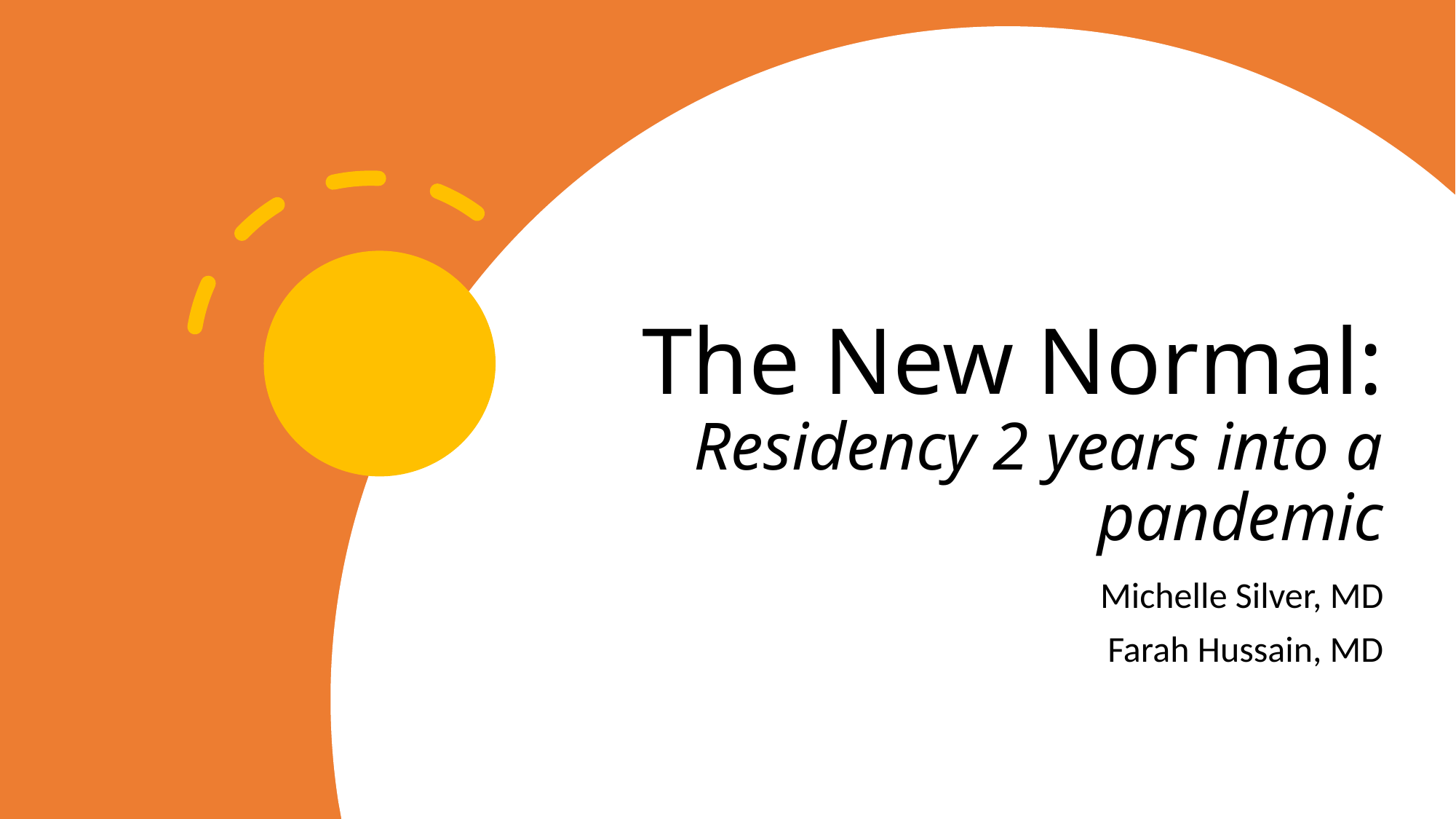

# The New Normal: Residency 2 years into a pandemic
Michelle Silver, MD
Farah Hussain, MD

## Slide 2
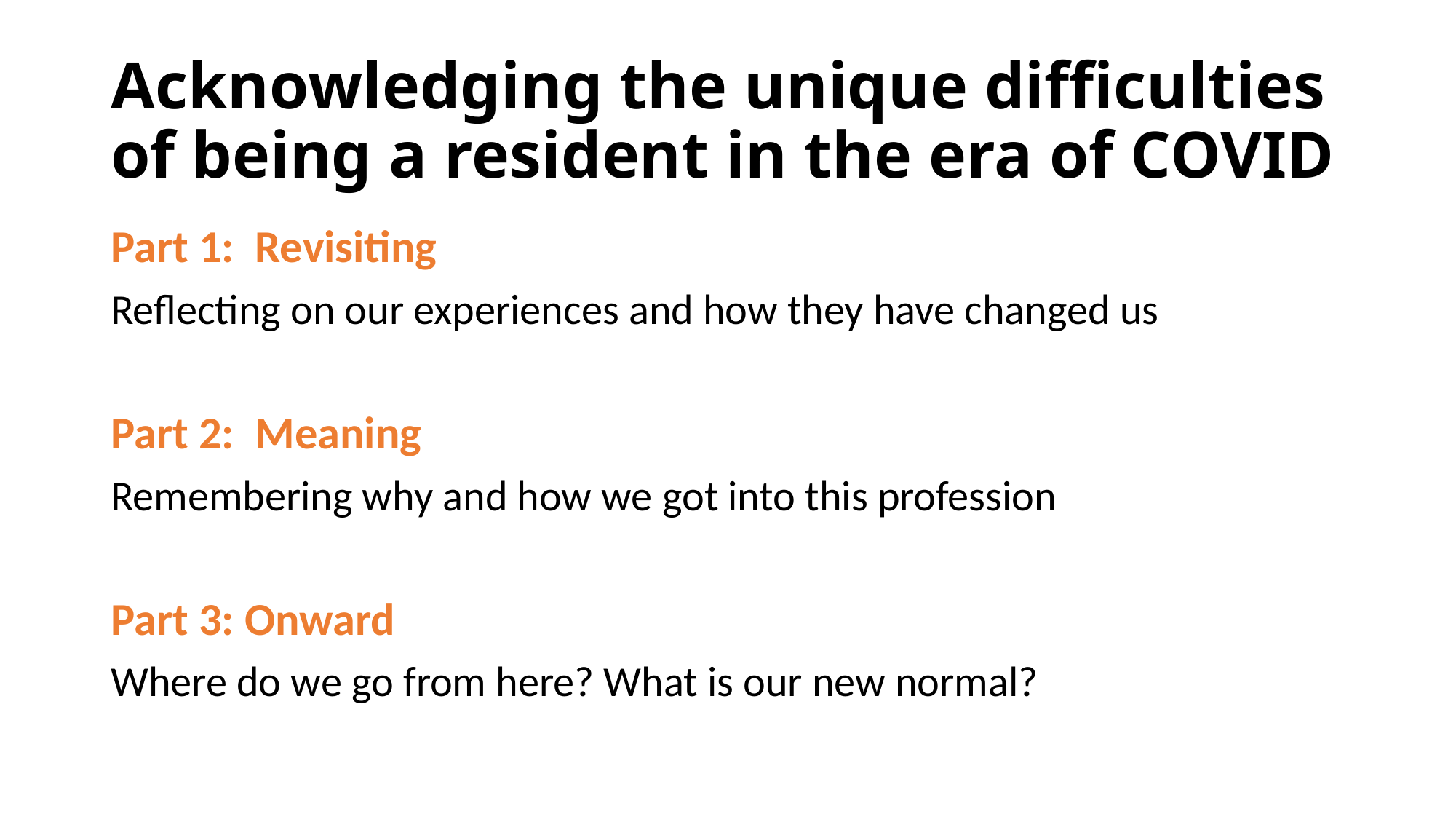

# Acknowledging the unique difficulties of being a resident in the era of COVID
Part 1: Revisiting
Reflecting on our experiences and how they have changed us
Part 2: Meaning
Remembering why and how we got into this profession
Part 3: Onward
Where do we go from here? What is our new normal?

## Slide 3
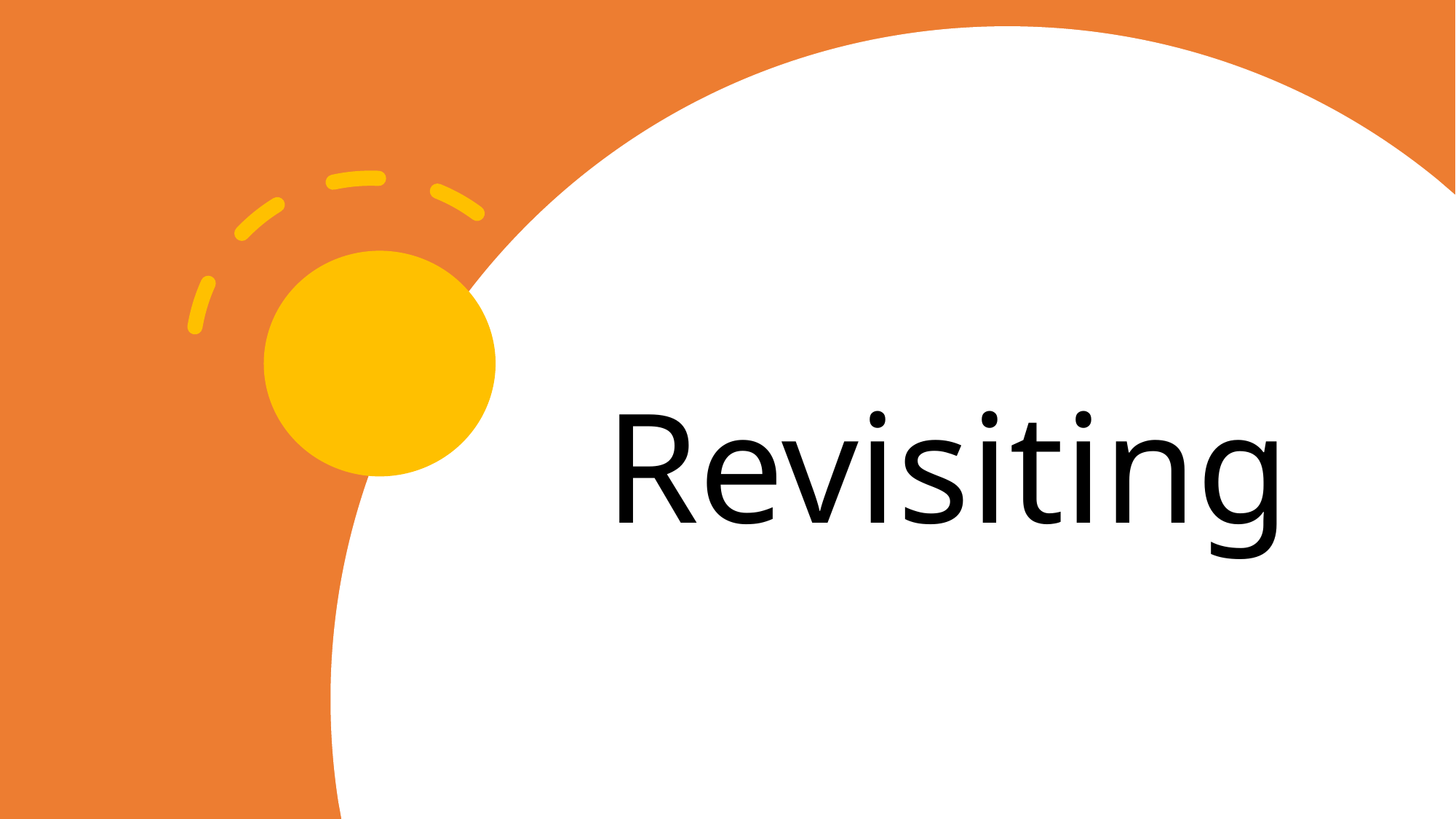

# Revisiting

## Slide 4
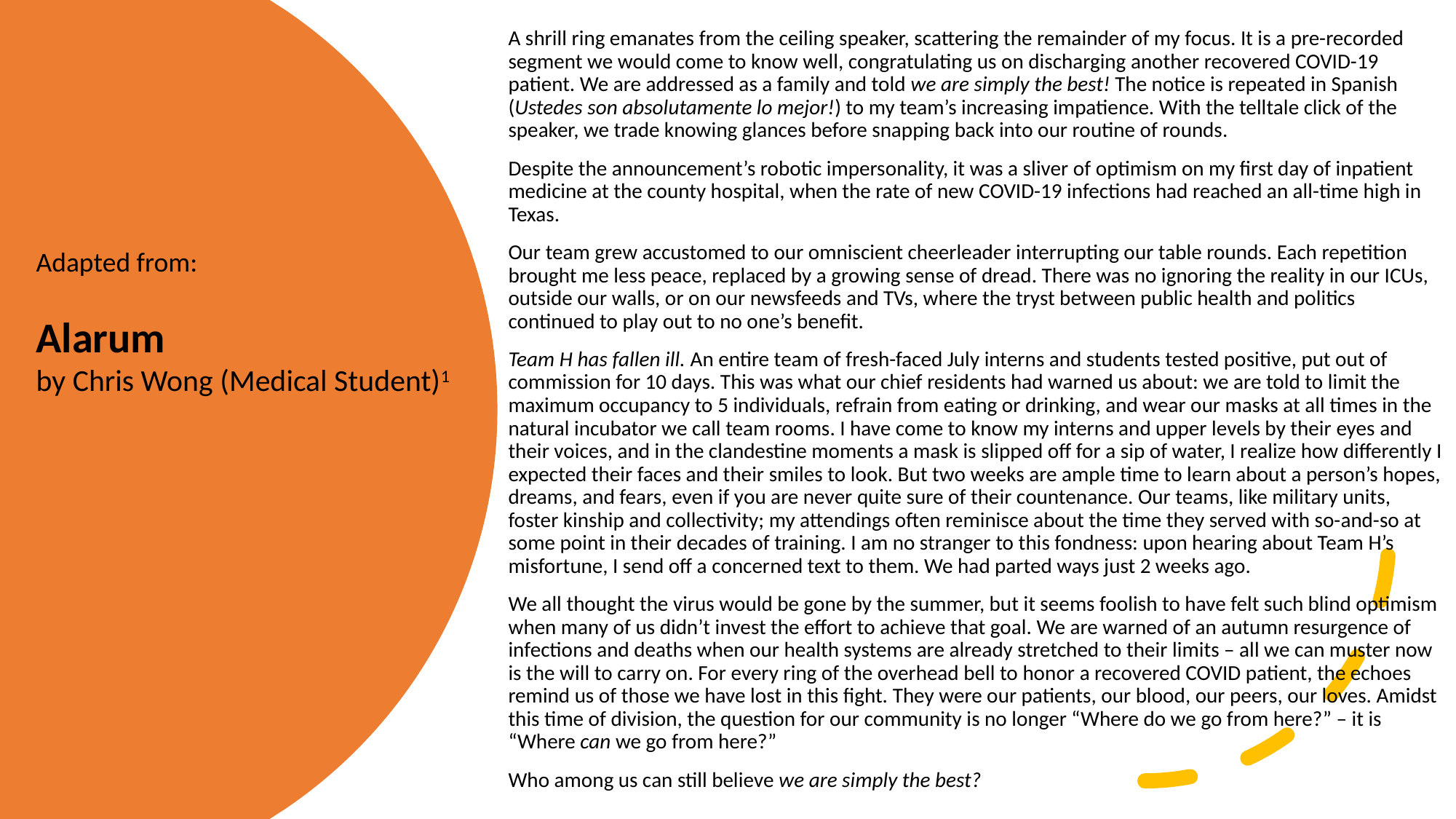

A shrill ring emanates from the ceiling speaker, scattering the remainder of my focus. It is a pre-recorded segment we would come to know well, congratulating us on discharging another recovered COVID-19 patient. We are addressed as a family and told we are simply the best! The notice is repeated in Spanish (Ustedes son absolutamente lo mejor!) to my team’s increasing impatience. With the telltale click of the speaker, we trade knowing glances before snapping back into our routine of rounds.
Despite the announcement’s robotic impersonality, it was a sliver of optimism on my first day of inpatient medicine at the county hospital, when the rate of new COVID-19 infections had reached an all-time high in Texas.
Our team grew accustomed to our omniscient cheerleader interrupting our table rounds. Each repetition brought me less peace, replaced by a growing sense of dread. There was no ignoring the reality in our ICUs, outside our walls, or on our newsfeeds and TVs, where the tryst between public health and politics continued to play out to no one’s benefit.
Team H has fallen ill. An entire team of fresh-faced July interns and students tested positive, put out of commission for 10 days. This was what our chief residents had warned us about: we are told to limit the maximum occupancy to 5 individuals, refrain from eating or drinking, and wear our masks at all times in the natural incubator we call team rooms. I have come to know my interns and upper levels by their eyes and their voices, and in the clandestine moments a mask is slipped off for a sip of water, I realize how differently I expected their faces and their smiles to look. But two weeks are ample time to learn about a person’s hopes, dreams, and fears, even if you are never quite sure of their countenance. Our teams, like military units, foster kinship and collectivity; my attendings often reminisce about the time they served with so-and-so at some point in their decades of training. I am no stranger to this fondness: upon hearing about Team H’s misfortune, I send off a concerned text to them. We had parted ways just 2 weeks ago.
We all thought the virus would be gone by the summer, but it seems foolish to have felt such blind optimism when many of us didn’t invest the effort to achieve that goal. We are warned of an autumn resurgence of infections and deaths when our health systems are already stretched to their limits – all we can muster now is the will to carry on. For every ring of the overhead bell to honor a recovered COVID patient, the echoes remind us of those we have lost in this fight. They were our patients, our blood, our peers, our loves. Amidst this time of division, the question for our community is no longer “Where do we go from here?” – it is “Where can we go from here?”
Who among us can still believe we are simply the best?
Adapted from:
Alarum
by Chris Wong (Medical Student)1

## Slide 5
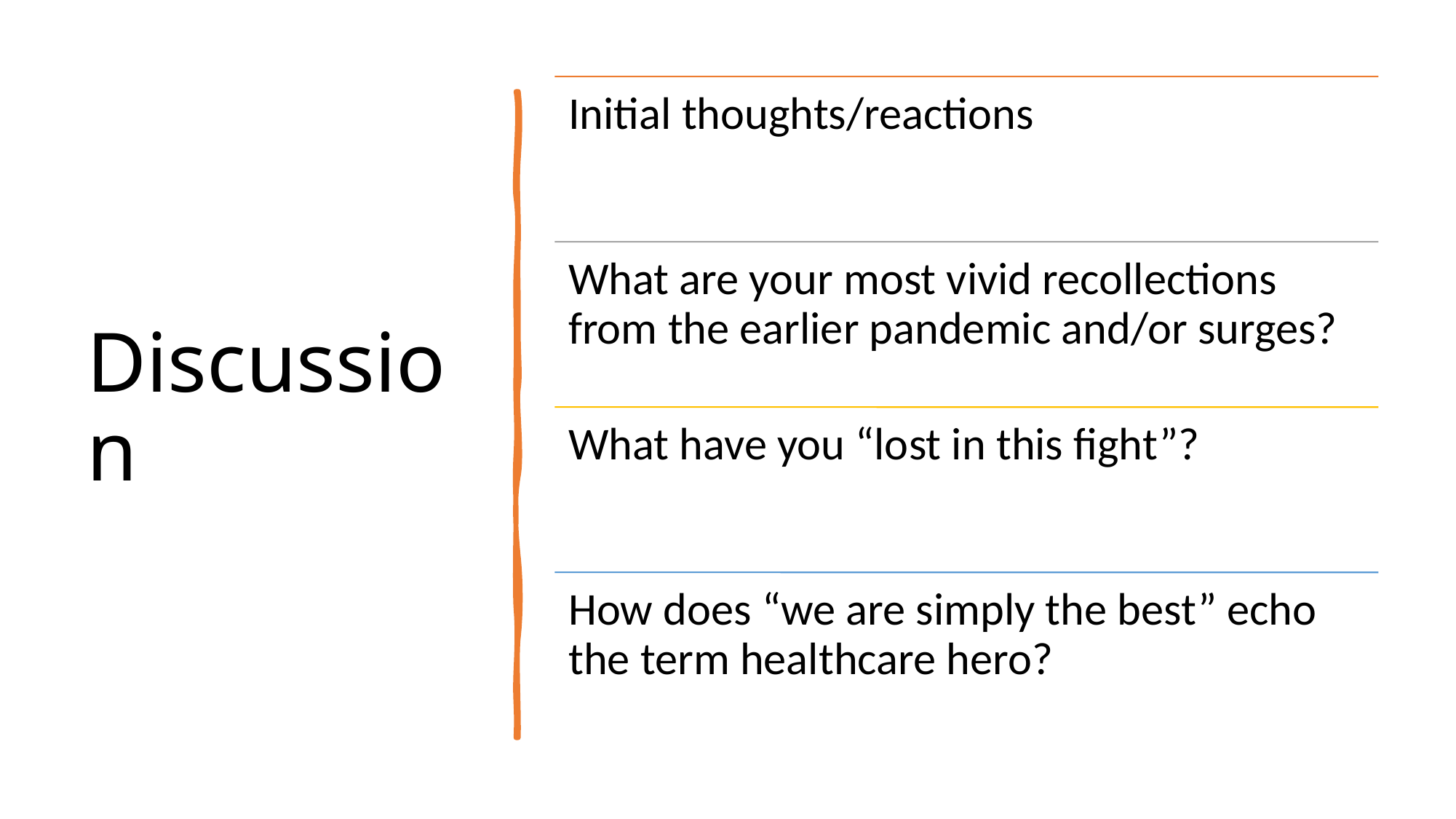

# Discussion

## Slide 6
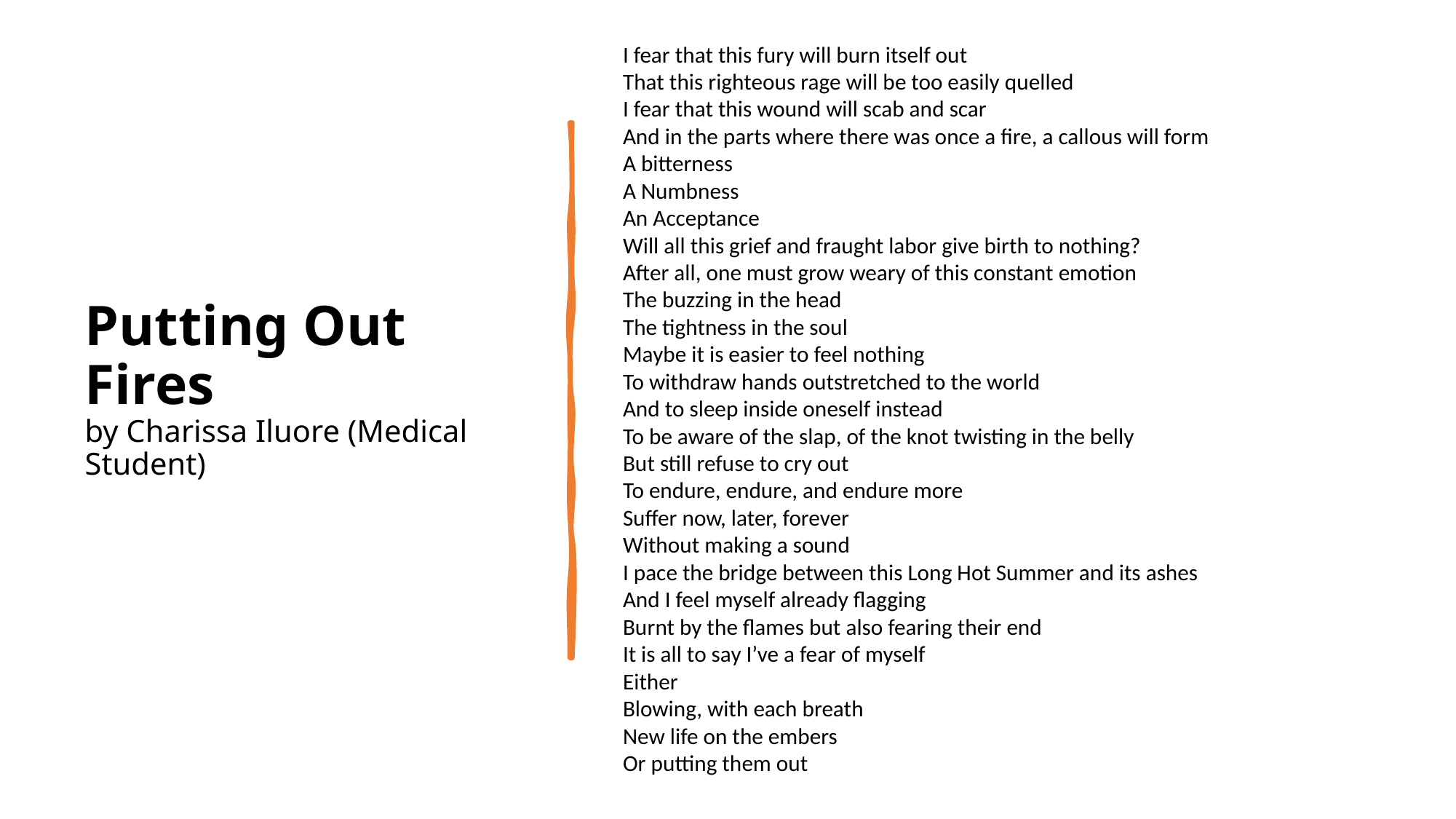

# Putting Out Fires by Charissa Iluore (Medical Student)
I fear that this fury will burn itself out
That this righteous rage will be too easily quelled
I fear that this wound will scab and scar
And in the parts where there was once a fire, a callous will form
A bitterness
A Numbness
An Acceptance
Will all this grief and fraught labor give birth to nothing?
After all, one must grow weary of this constant emotion
The buzzing in the head
The tightness in the soul
Maybe it is easier to feel nothing
To withdraw hands outstretched to the world
And to sleep inside oneself instead
To be aware of the slap, of the knot twisting in the belly
But still refuse to cry out
To endure, endure, and endure more
Suffer now, later, forever
Without making a sound
I pace the bridge between this Long Hot Summer and its ashes
And I feel myself already flagging
Burnt by the flames but also fearing their end
It is all to say I’ve a fear of myself
Either
Blowing, with each breath
New life on the embers
Or putting them out

## Slide 7
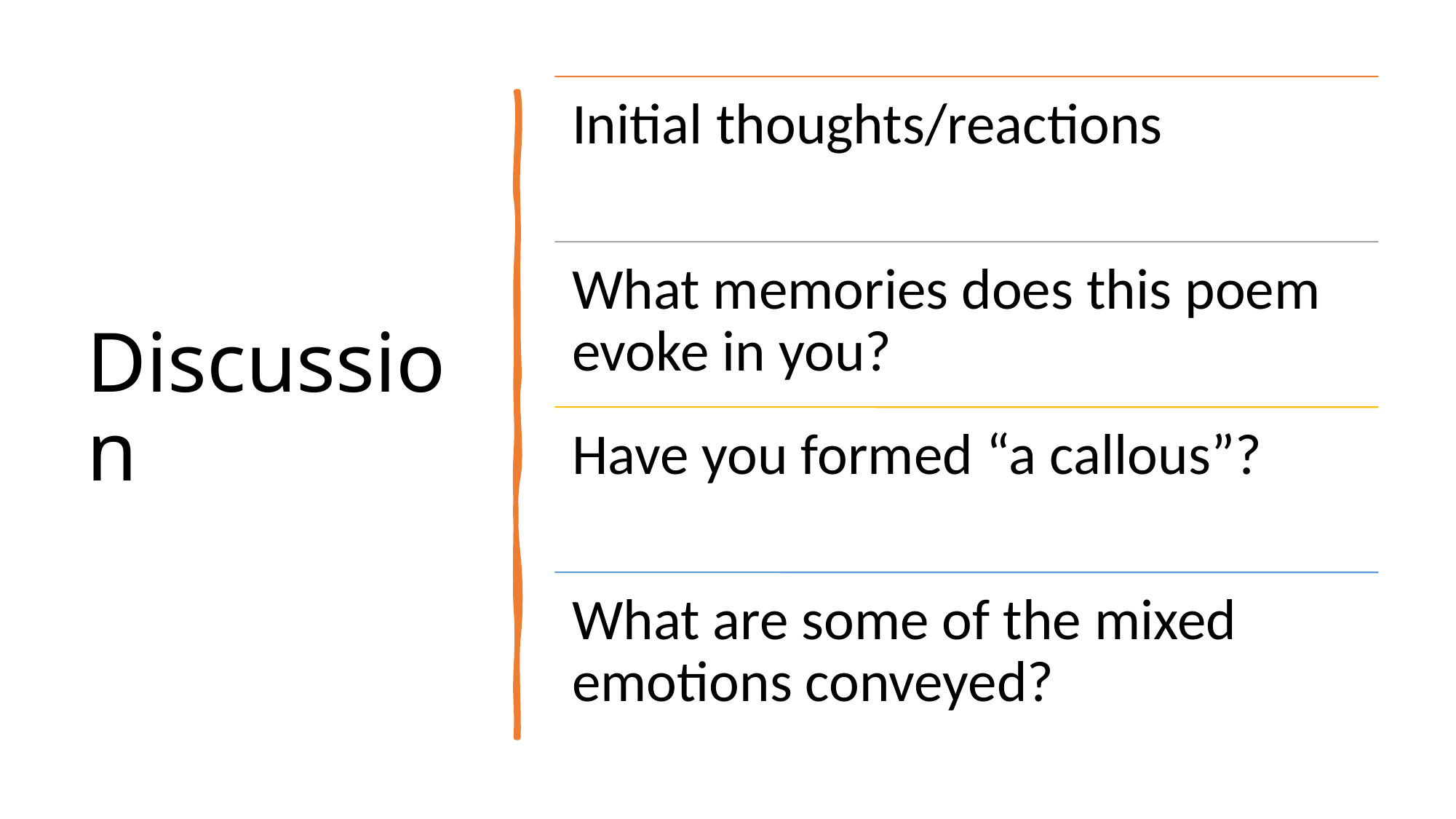

# Discussion

## Slide 8
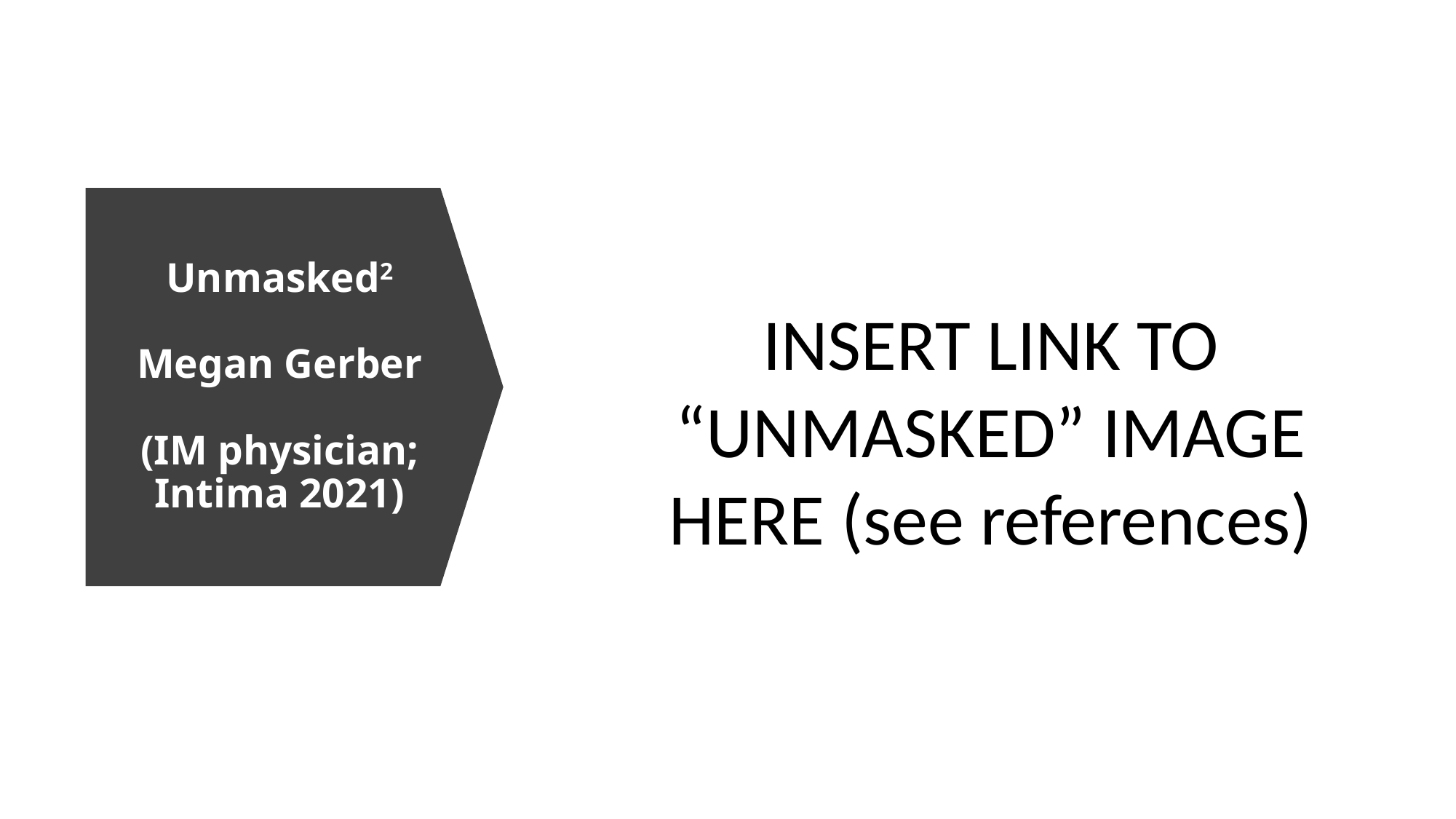

INSERT LINK TO “UNMASKED” IMAGE HERE (see references)
# Unmasked2 Megan Gerber (IM physician; Intima 2021)

## Slide 9
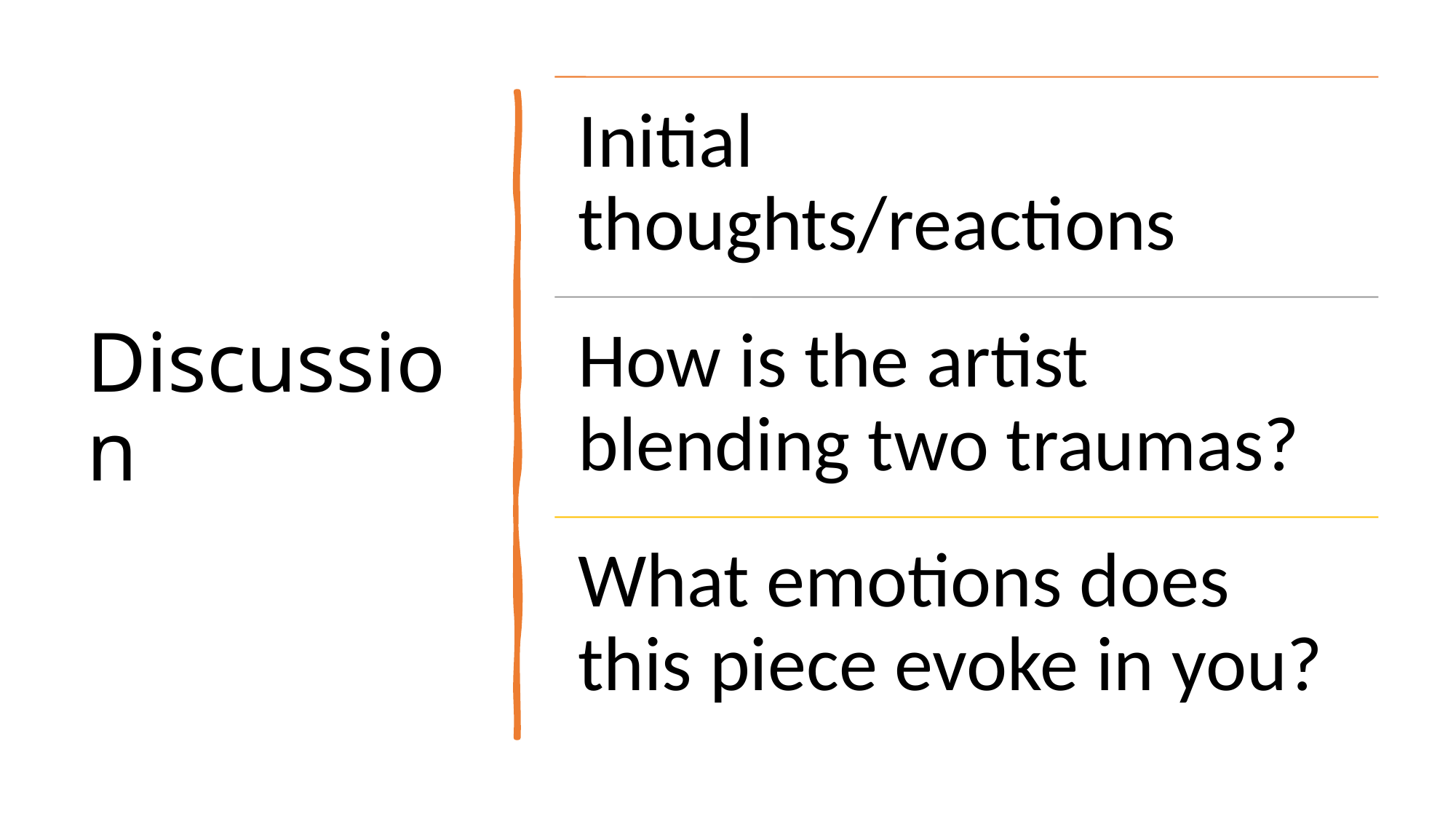

# Discussion

## Slide 10
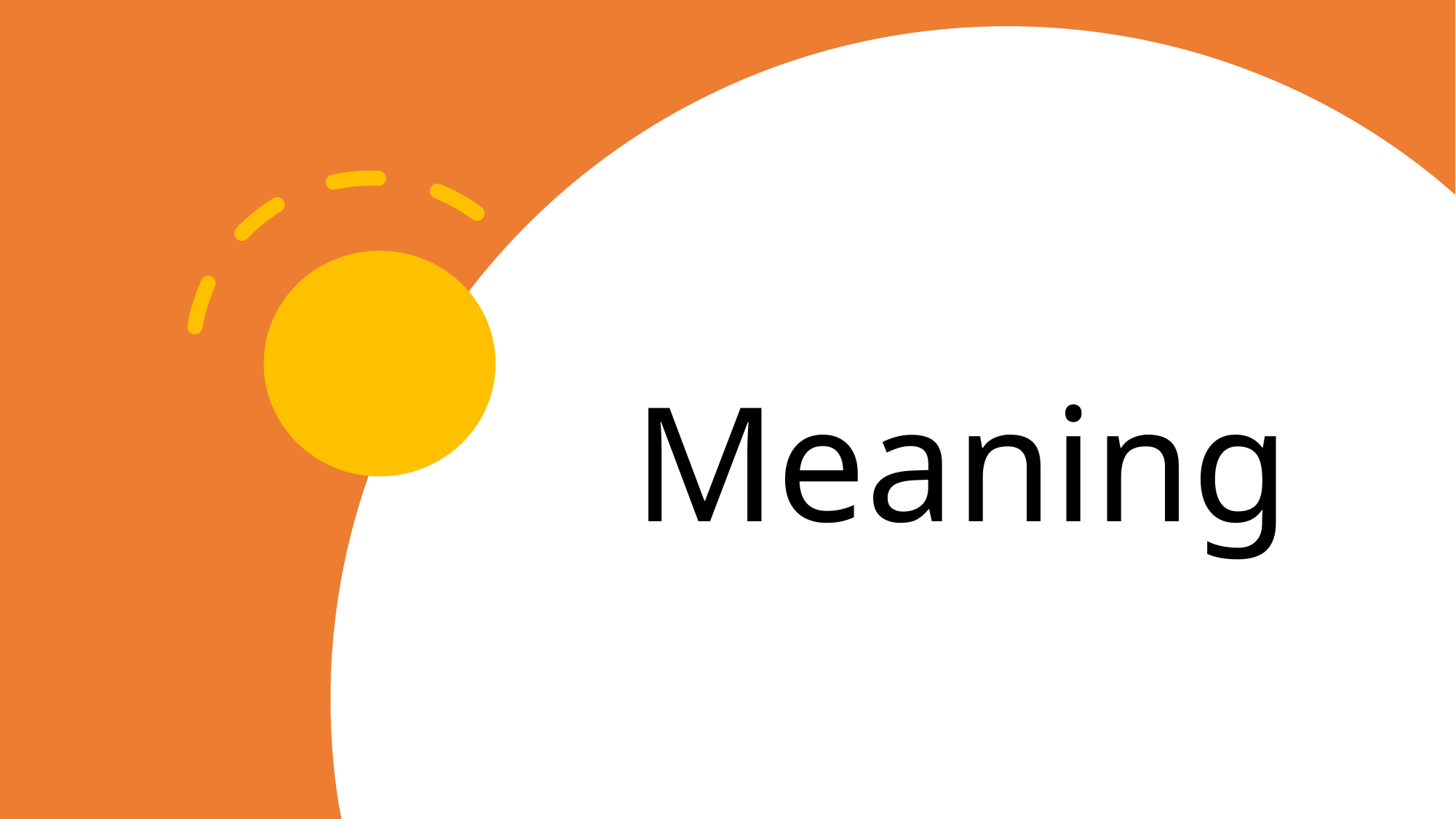

# Meaning

## Slide 11
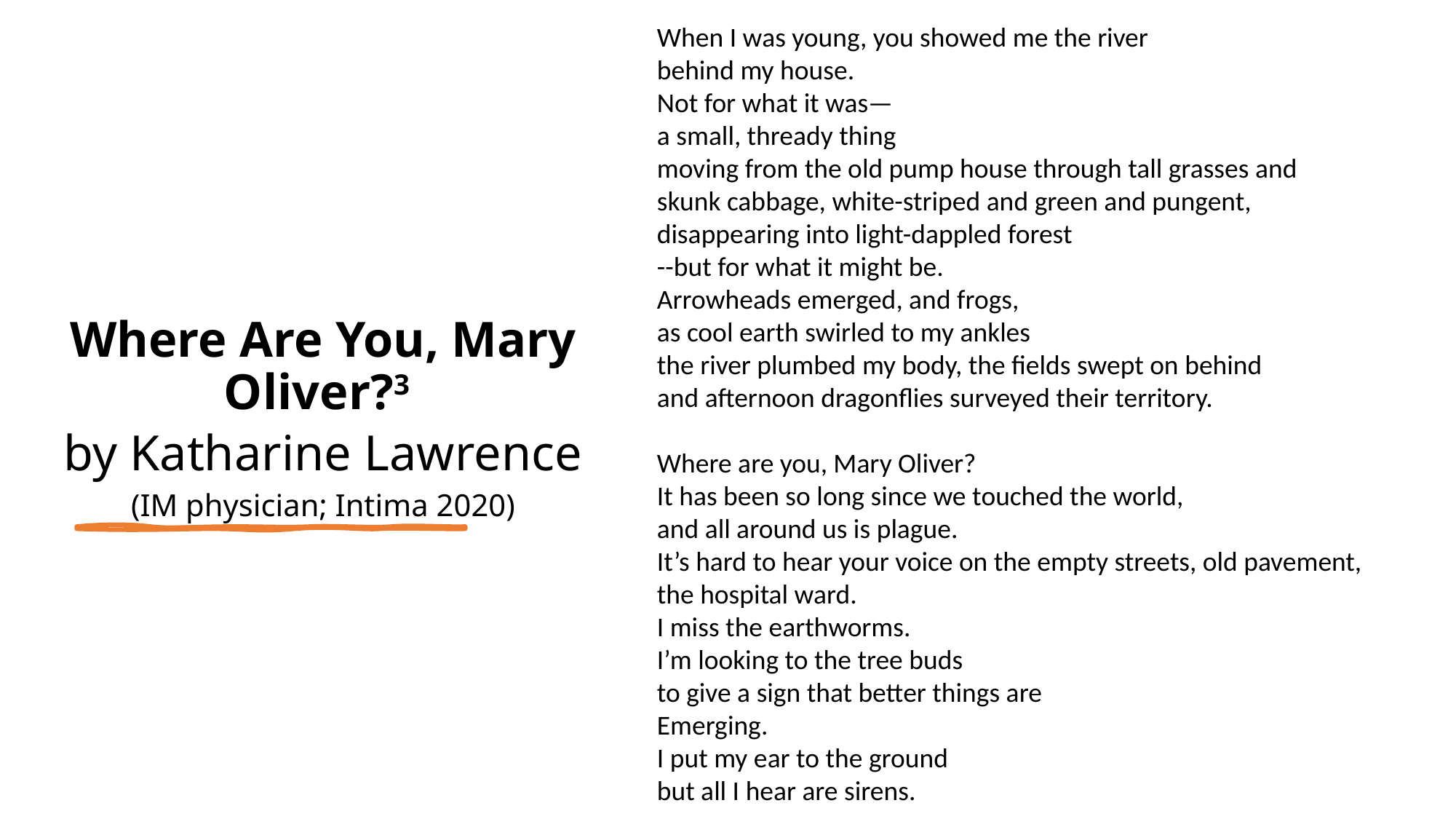

When I was young, you showed me the river
behind my house.
Not for what it was—
a small, thready thing
moving from the old pump house through tall grasses and
skunk cabbage, white-striped and green and pungent,
disappearing into light-dappled forest
--but for what it might be.
Arrowheads emerged, and frogs,
as cool earth swirled to my ankles
the river plumbed my body, the fields swept on behind
and afternoon dragonflies surveyed their territory.
Where are you, Mary Oliver?
It has been so long since we touched the world,
and all around us is plague.
It’s hard to hear your voice on the empty streets, old pavement,
the hospital ward.
I miss the earthworms.
I’m looking to the tree buds
to give a sign that better things are
Emerging.
I put my ear to the ground
but all I hear are sirens.
Where Are You, Mary Oliver?3
by Katharine Lawrence
(IM physician; Intima 2020)

## Slide 12
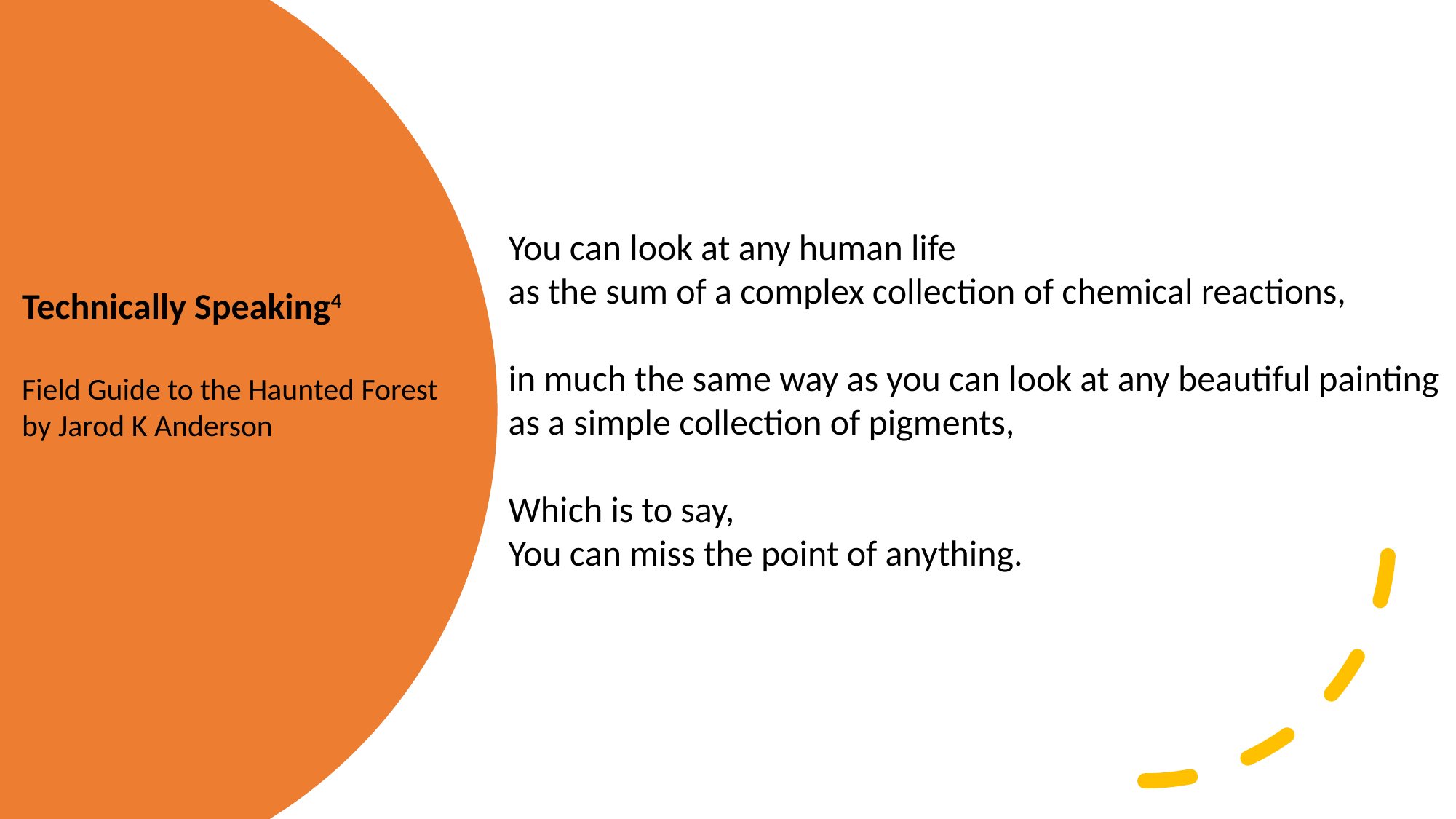

You can look at any human life
as the sum of a complex collection of chemical reactions,
in much the same way as you can look at any beautiful painting
as a simple collection of pigments,
Which is to say,
You can miss the point of anything.
Technically Speaking4
Field Guide to the Haunted Forest
by Jarod K Anderson

## Slide 13
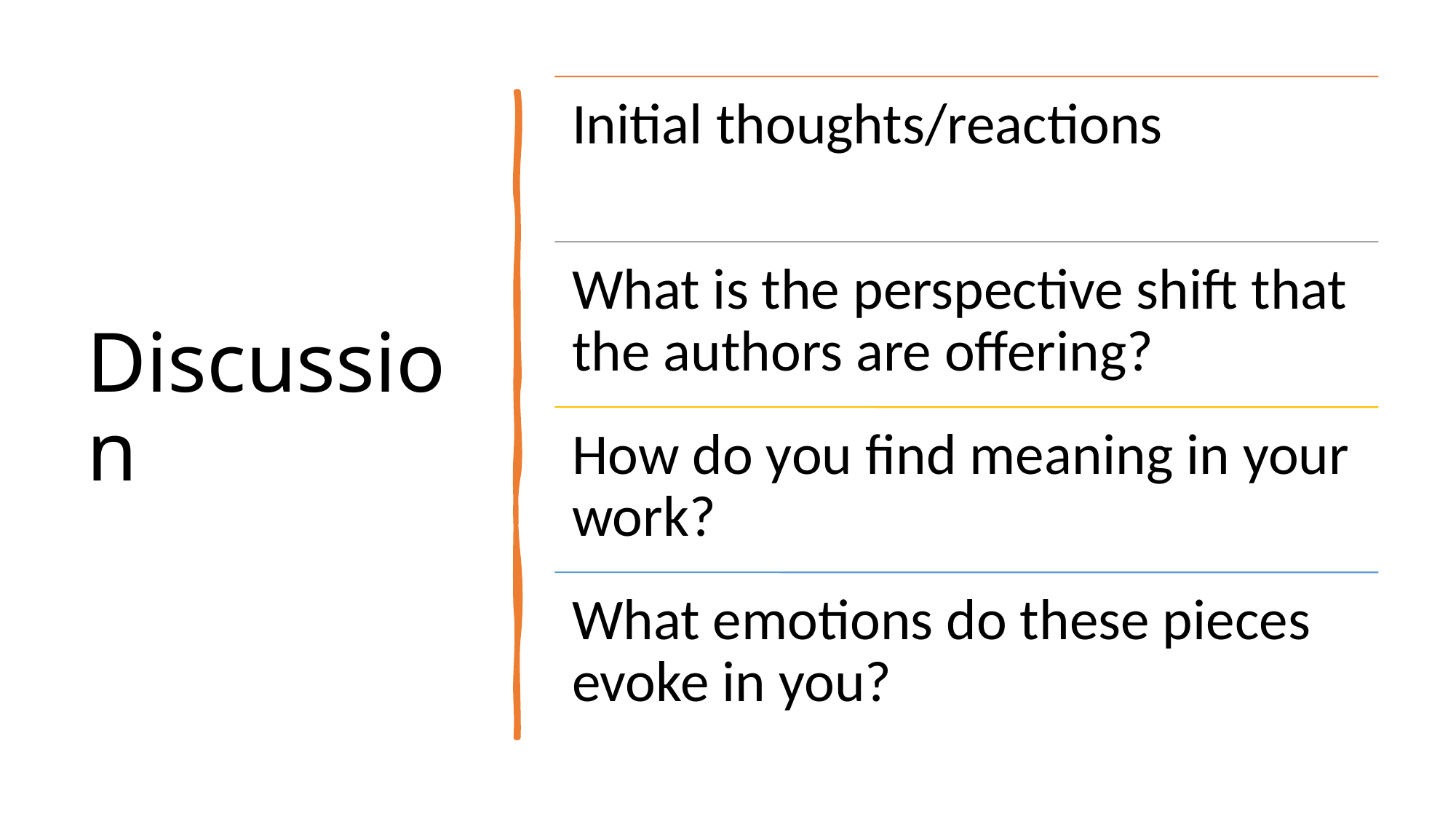

# Discussion

## Slide 14
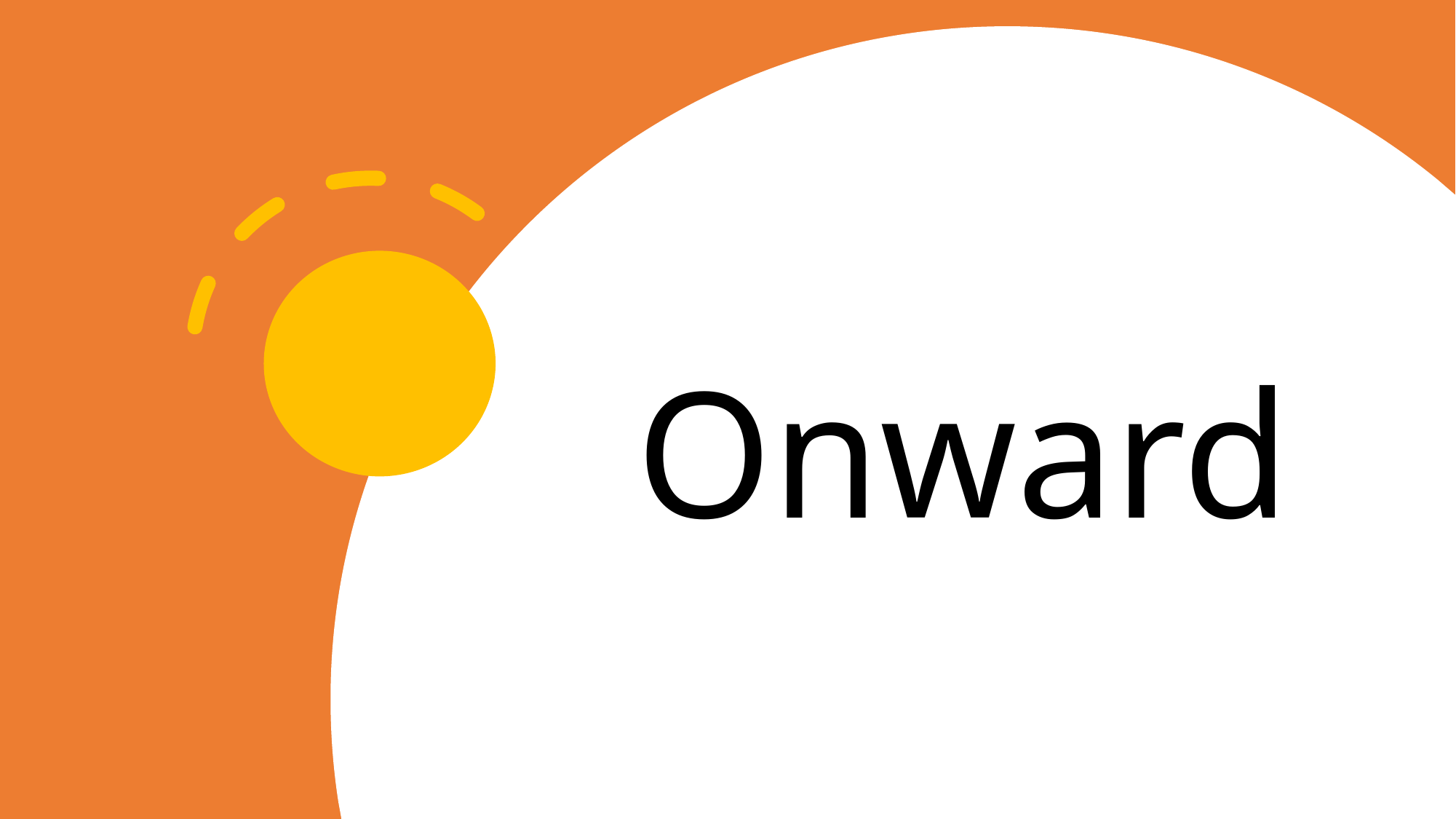

# Onward

## Slide 15
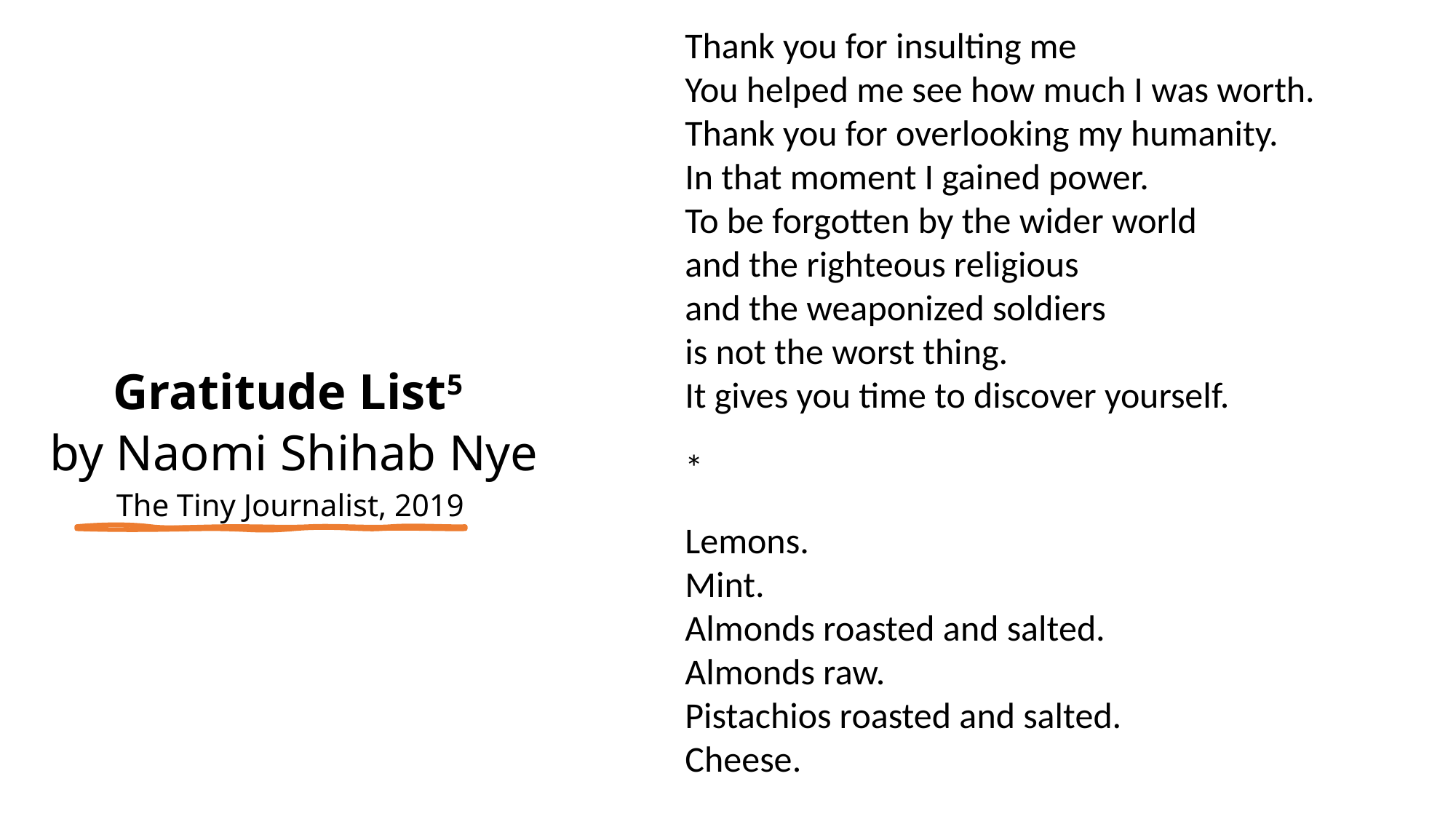

Thank you for insulting me
You helped me see how much I was worth.
Thank you for overlooking my humanity.
In that moment I gained power.
To be forgotten by the wider world
and the righteous religious
and the weaponized soldiers
is not the worst thing.
It gives you time to discover yourself.
*
Lemons.
Mint.
Almonds roasted and salted.
Almonds raw.
Pistachios roasted and salted.
Cheese.
Gratitude List5
by Naomi Shihab Nye
The Tiny Journalist, 2019

## Slide 16
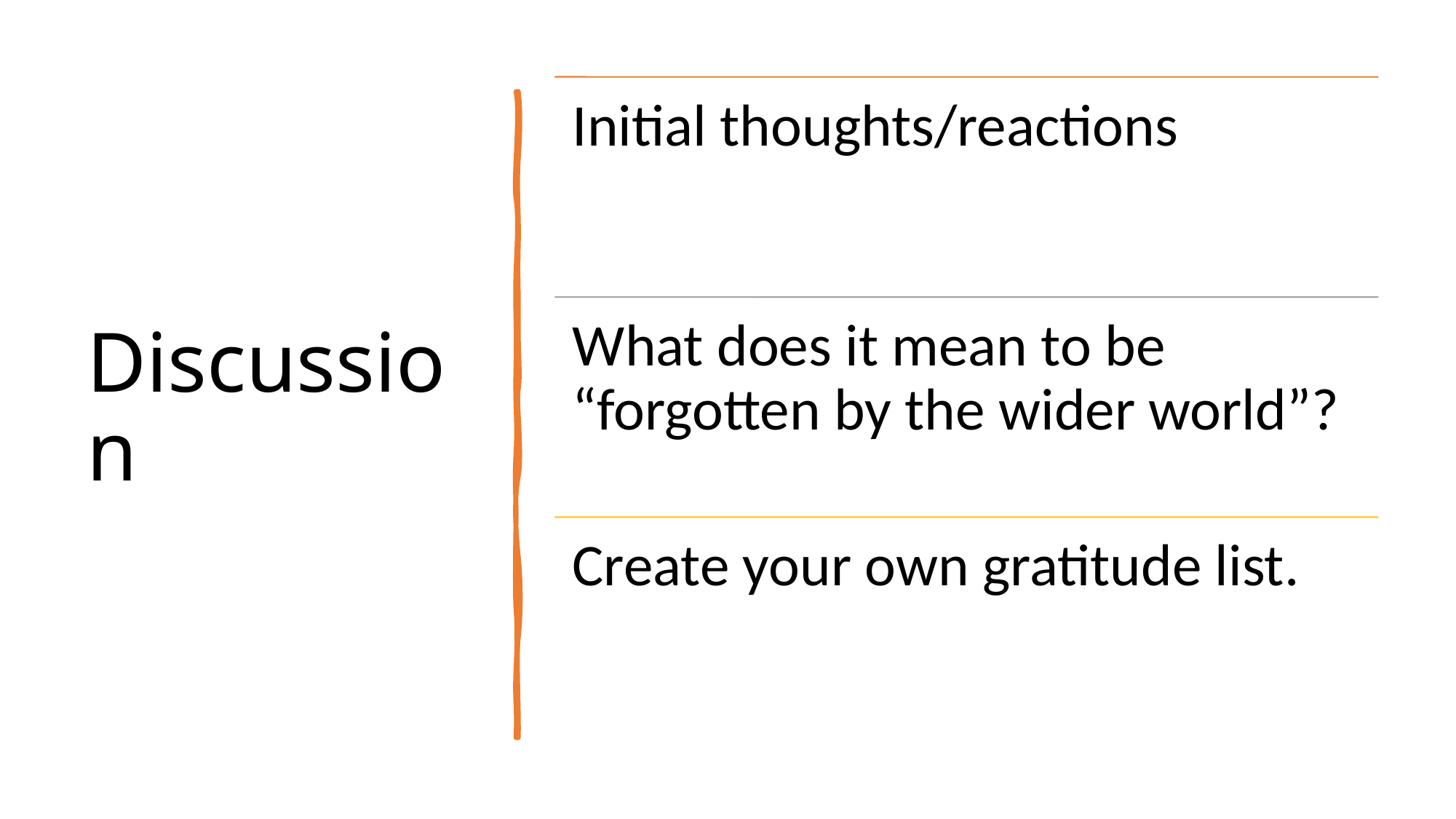

# Discussion

## Slide 17
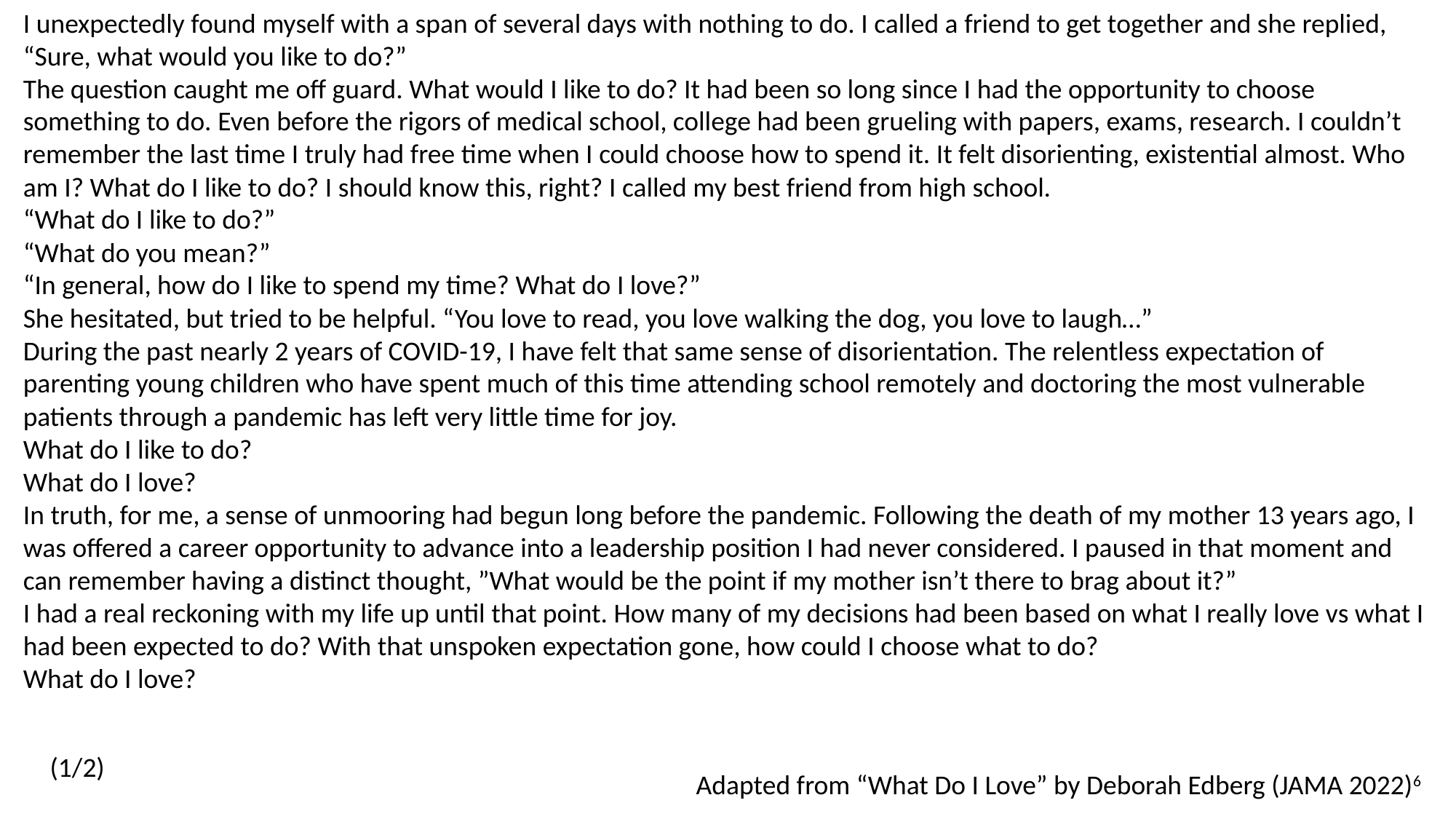

I unexpectedly found myself with a span of several days with nothing to do. I called a friend to get together and she replied, “Sure, what would you like to do?”
The question caught me off guard. What would I like to do? It had been so long since I had the opportunity to choose something to do. Even before the rigors of medical school, college had been grueling with papers, exams, research. I couldn’t remember the last time I truly had free time when I could choose how to spend it. It felt disorienting, existential almost. Who am I? What do I like to do? I should know this, right? I called my best friend from high school.
“What do I like to do?”
“What do you mean?”
“In general, how do I like to spend my time? What do I love?”
She hesitated, but tried to be helpful. “You love to read, you love walking the dog, you love to laugh…”
During the past nearly 2 years of COVID-19, I have felt that same sense of disorientation. The relentless expectation of parenting young children who have spent much of this time attending school remotely and doctoring the most vulnerable patients through a pandemic has left very little time for joy.
What do I like to do?
What do I love?
In truth, for me, a sense of unmooring had begun long before the pandemic. Following the death of my mother 13 years ago, I was offered a career opportunity to advance into a leadership position I had never considered. I paused in that moment and can remember having a distinct thought, ”What would be the point if my mother isn’t there to brag about it?”
I had a real reckoning with my life up until that point. How many of my decisions had been based on what I really love vs what I had been expected to do? With that unspoken expectation gone, how could I choose what to do?
What do I love?
(1/2)
Adapted from “What Do I Love” by Deborah Edberg (JAMA 2022)6

## Slide 18
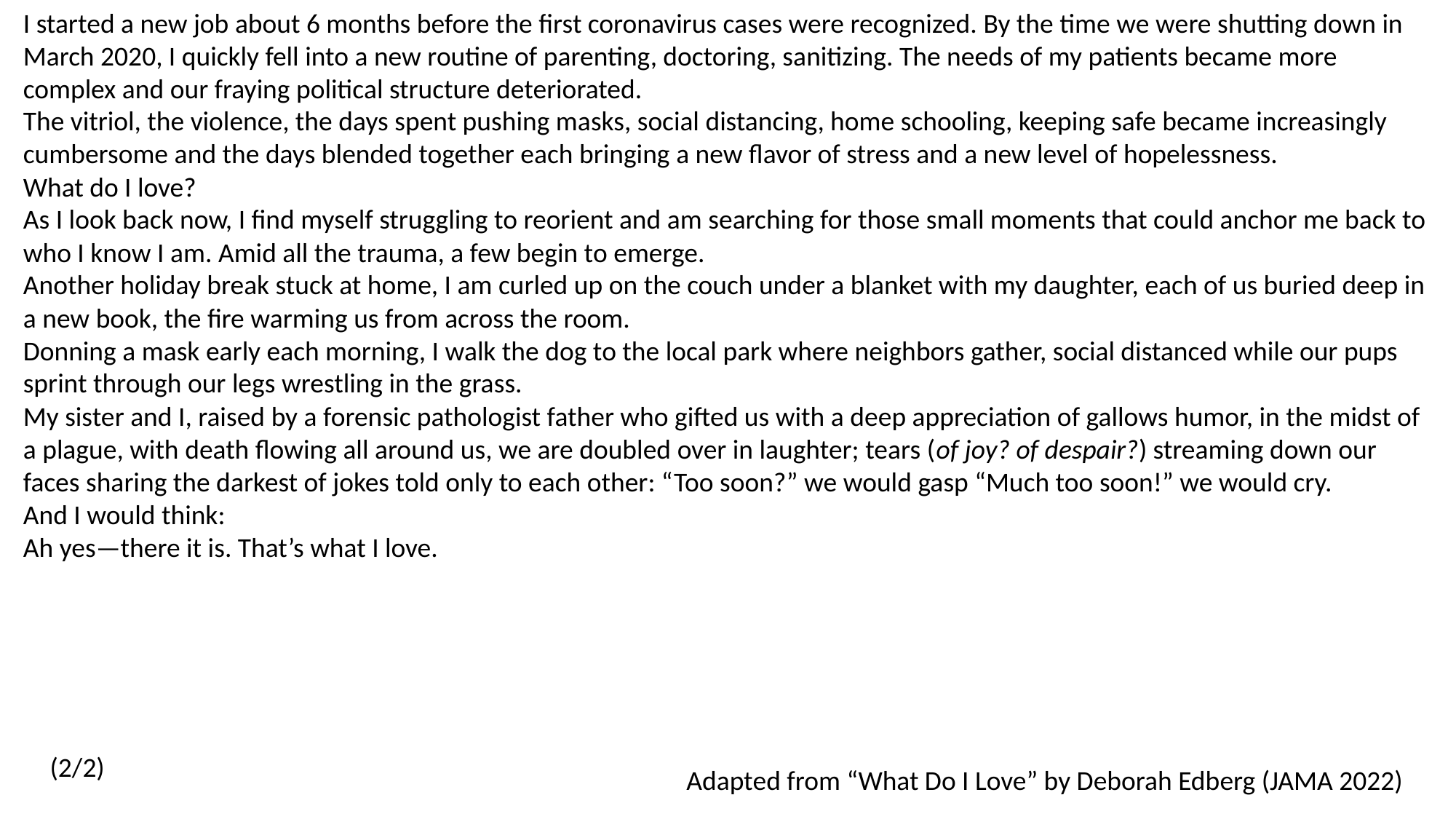

I started a new job about 6 months before the first coronavirus cases were recognized. By the time we were shutting down in March 2020, I quickly fell into a new routine of parenting, doctoring, sanitizing. The needs of my patients became more complex and our fraying political structure deteriorated.
The vitriol, the violence, the days spent pushing masks, social distancing, home schooling, keeping safe became increasingly cumbersome and the days blended together each bringing a new flavor of stress and a new level of hopelessness.
What do I love?
As I look back now, I find myself struggling to reorient and am searching for those small moments that could anchor me back to who I know I am. Amid all the trauma, a few begin to emerge.
Another holiday break stuck at home, I am curled up on the couch under a blanket with my daughter, each of us buried deep in a new book, the fire warming us from across the room.
Donning a mask early each morning, I walk the dog to the local park where neighbors gather, social distanced while our pups sprint through our legs wrestling in the grass.
My sister and I, raised by a forensic pathologist father who gifted us with a deep appreciation of gallows humor, in the midst of a plague, with death flowing all around us, we are doubled over in laughter; tears (of joy? of despair?) streaming down our faces sharing the darkest of jokes told only to each other: “Too soon?” we would gasp “Much too soon!” we would cry.
And I would think:
Ah yes—there it is. That’s what I love.
(2/2)
Adapted from “What Do I Love” by Deborah Edberg (JAMA 2022)

## Slide 19
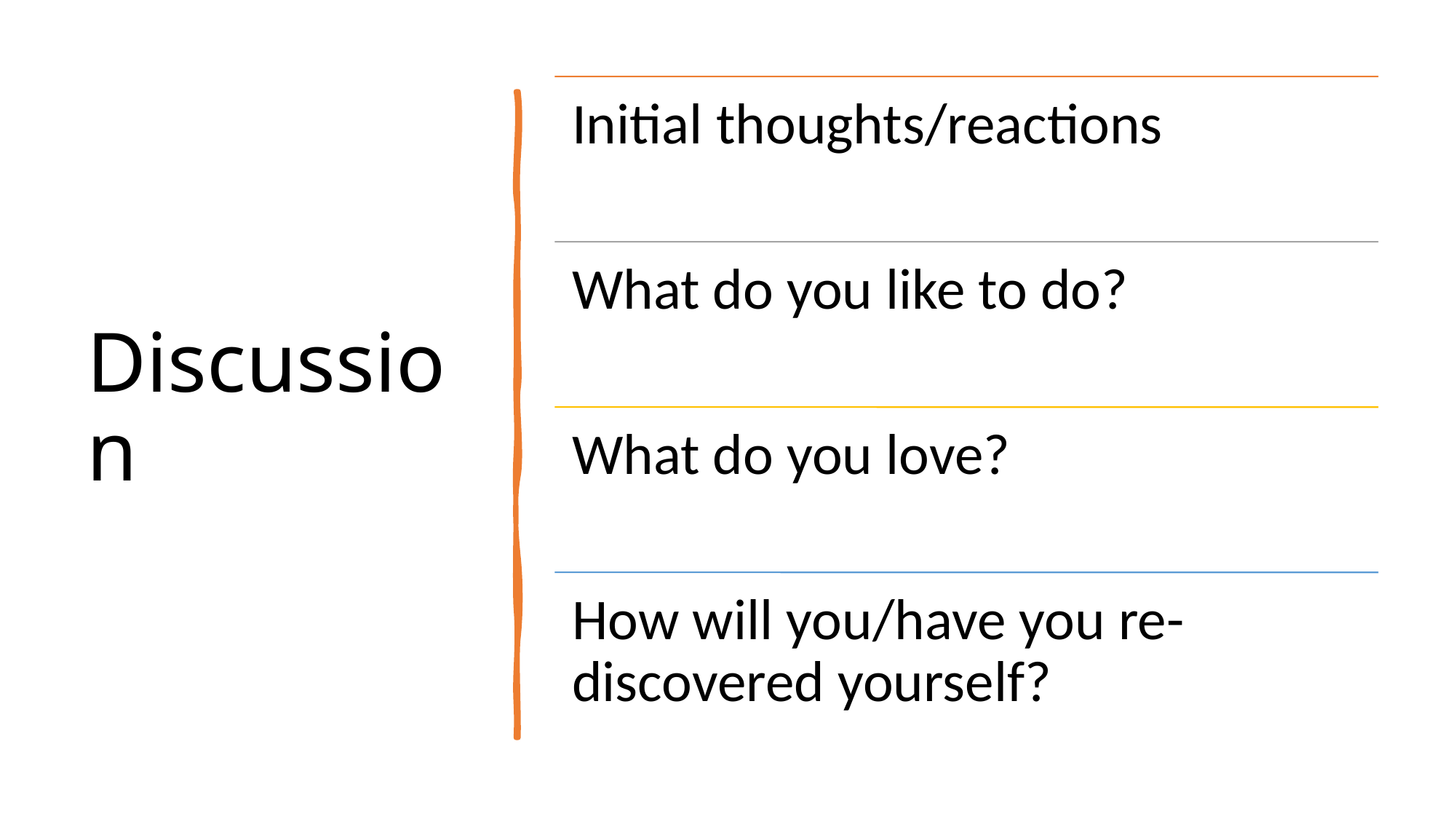

# Discussion

## Slide 20
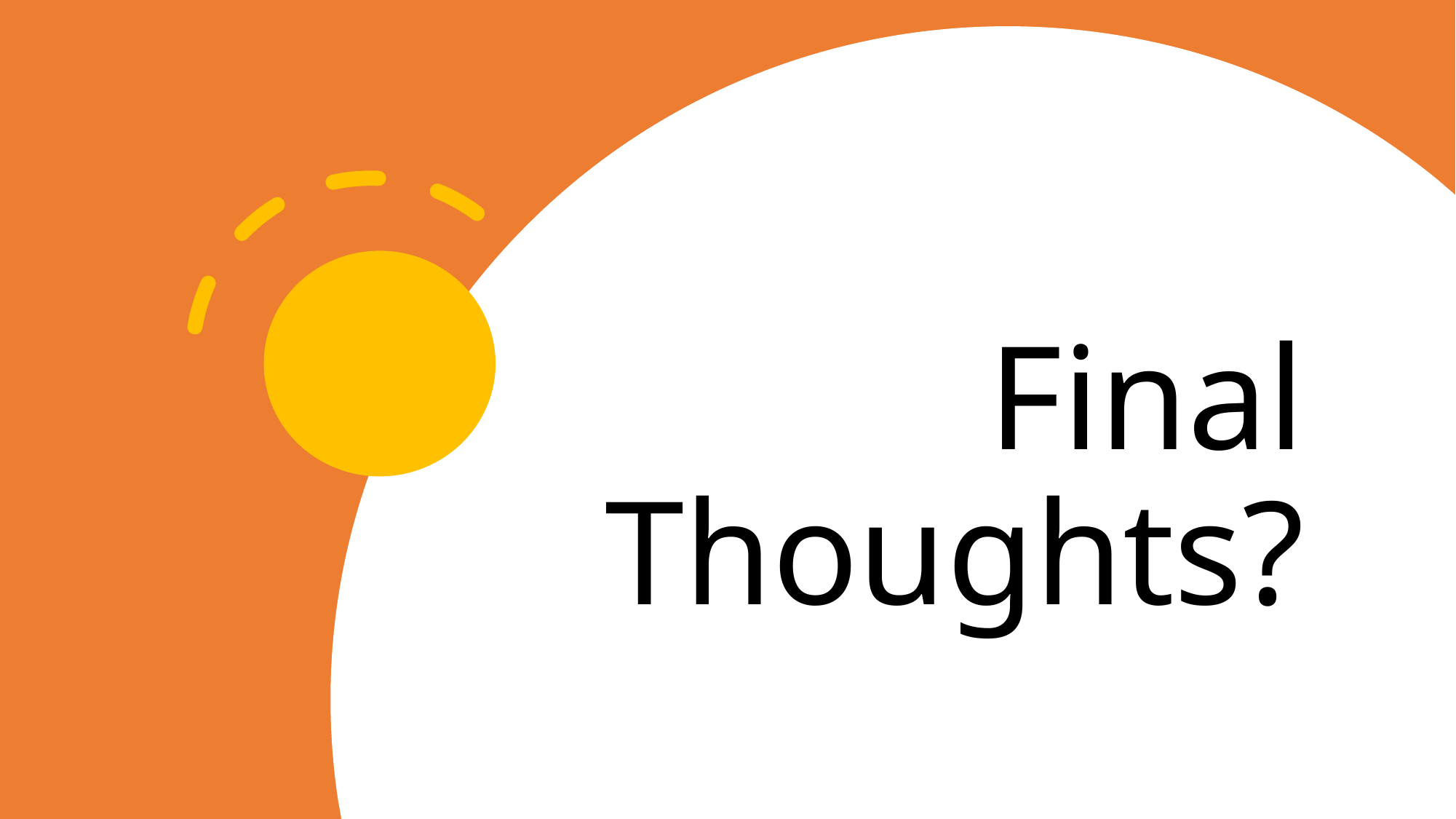

# Final Thoughts?

## Slide 21
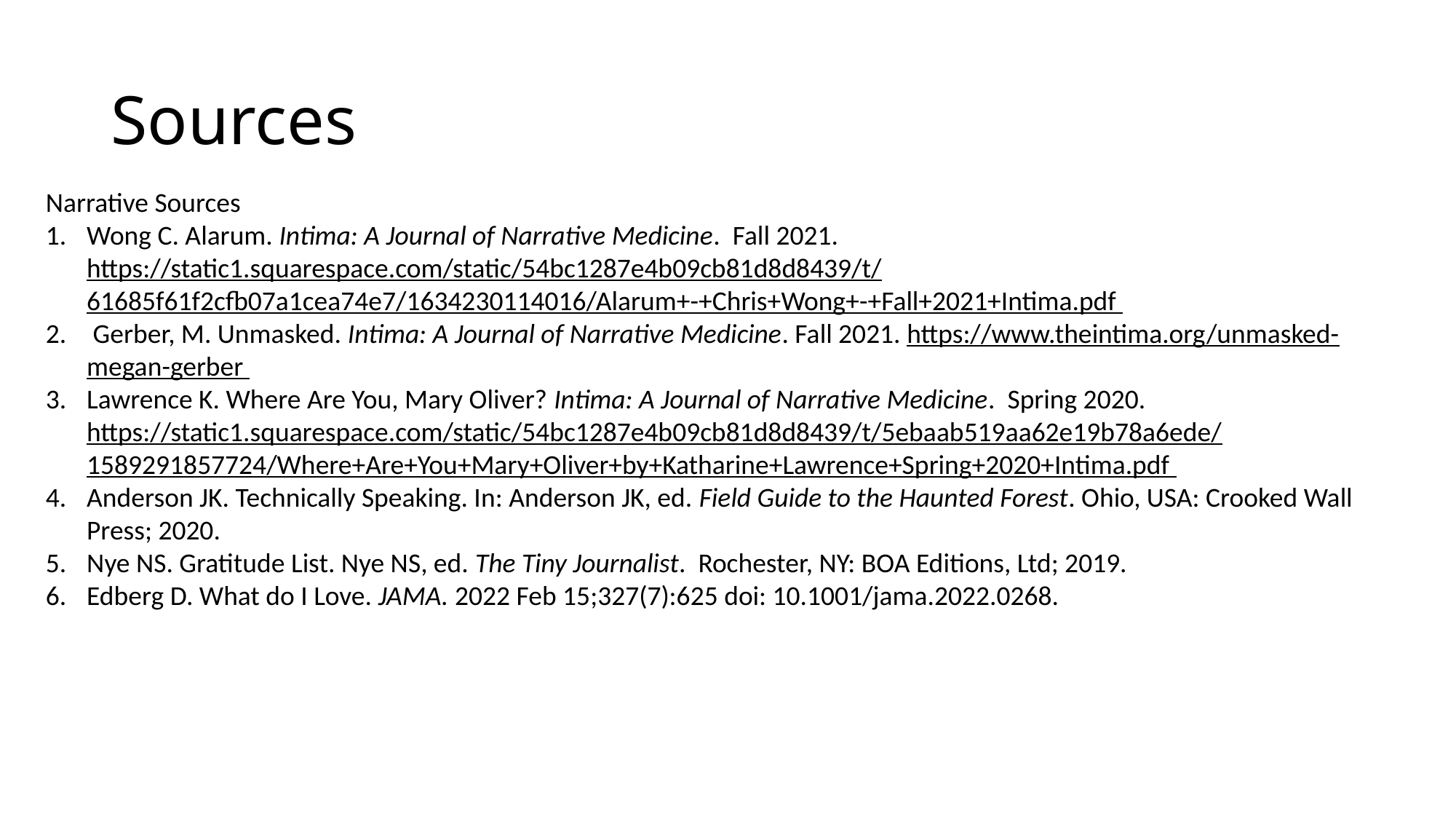

# Sources
Narrative Sources
Wong C. Alarum. Intima: A Journal of Narrative Medicine. Fall 2021. https://static1.squarespace.com/static/54bc1287e4b09cb81d8d8439/t/61685f61f2cfb07a1cea74e7/1634230114016/Alarum+-+Chris+Wong+-+Fall+2021+Intima.pdf
 Gerber, M. Unmasked. Intima: A Journal of Narrative Medicine. Fall 2021. https://www.theintima.org/unmasked-megan-gerber
Lawrence K. Where Are You, Mary Oliver? Intima: A Journal of Narrative Medicine. Spring 2020. https://static1.squarespace.com/static/54bc1287e4b09cb81d8d8439/t/5ebaab519aa62e19b78a6ede/1589291857724/Where+Are+You+Mary+Oliver+by+Katharine+Lawrence+Spring+2020+Intima.pdf
Anderson JK. Technically Speaking. In: Anderson JK, ed. Field Guide to the Haunted Forest. Ohio, USA: Crooked Wall Press; 2020.
Nye NS. Gratitude List. Nye NS, ed. The Tiny Journalist. Rochester, NY: BOA Editions, Ltd; 2019.
Edberg D. What do I Love. JAMA. 2022 Feb 15;327(7):625 doi: 10.1001/jama.2022.0268.

## Slide 22
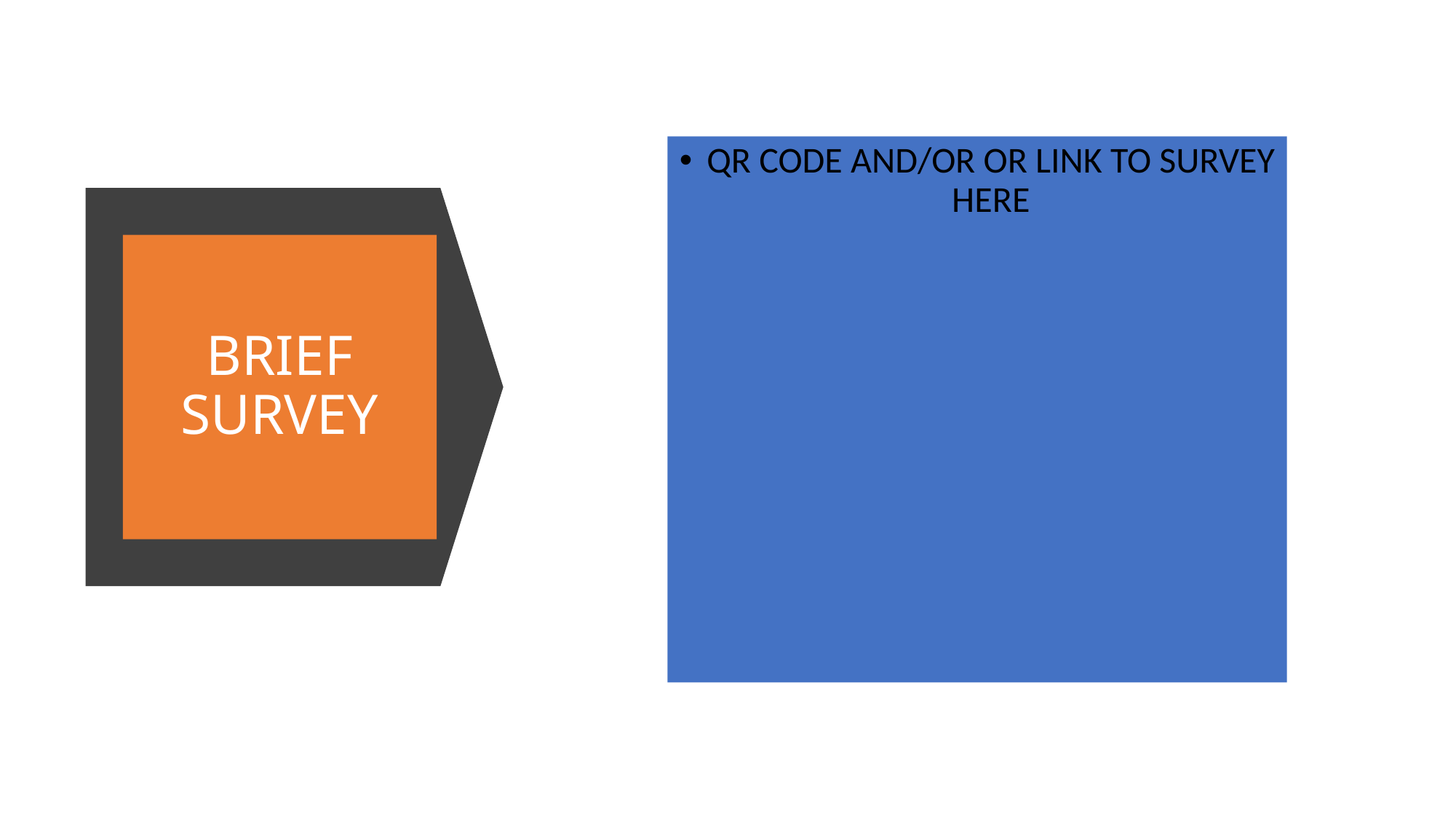

QR CODE AND/OR OR LINK TO SURVEY HERE
# BRIEF SURVEY
