# Supplementary material for: A Resident Narrative Medicine Curriculum to Promote Professional Identity Development: Story-Based Sessions Grounded in Narrative Learning Theory
Source: MedEdPORTAL. 2024 Oct 22;20:11446. doi: 10.15766/mep_2374-8265.11446 (PMC11493853; doi:10.15766/mep_2374-8265.11446)
Supplement: Supplementary file 1 — Facilitator Guide.docxBurnout and Moral Injury.pptxCompassion Fatigue.pptxWorking Through a Pandemic.pptxDifficult Patient.pptxThe New Normal.pptxFinding Meaning.pptxUnpublished Narratives.docxSurvey.docx [file mep_2374-8265.11446-s001.zip › G. Finding Meaning.pptx]

## Slide 1
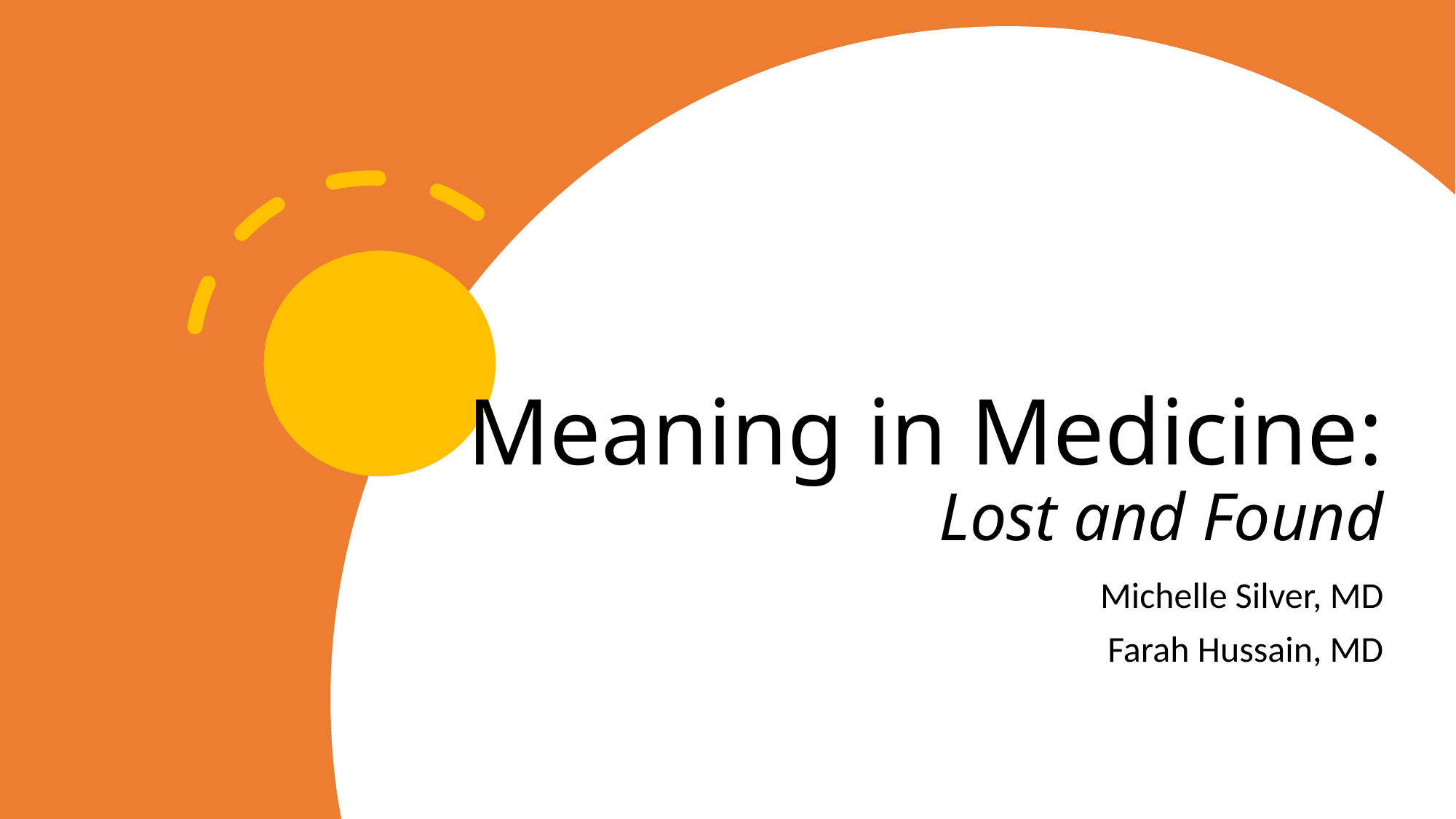

# Meaning in Medicine: Lost and Found
Michelle Silver, MD
Farah Hussain, MD

## Slide 2
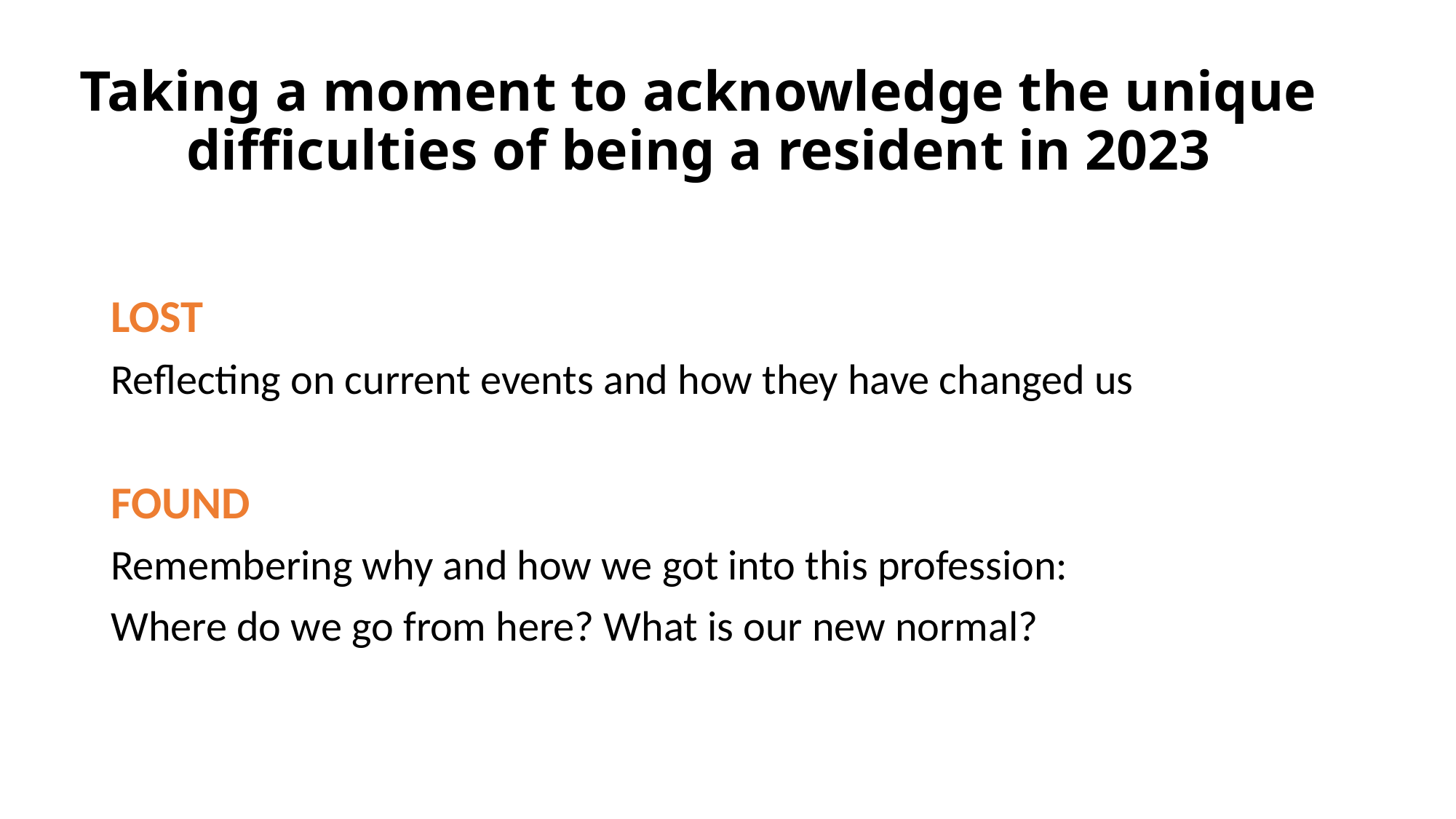

# Taking a moment to acknowledge the unique difficulties of being a resident in 2023
LOST
Reflecting on current events and how they have changed us
FOUND
Remembering why and how we got into this profession:
Where do we go from here? What is our new normal?

## Slide 3
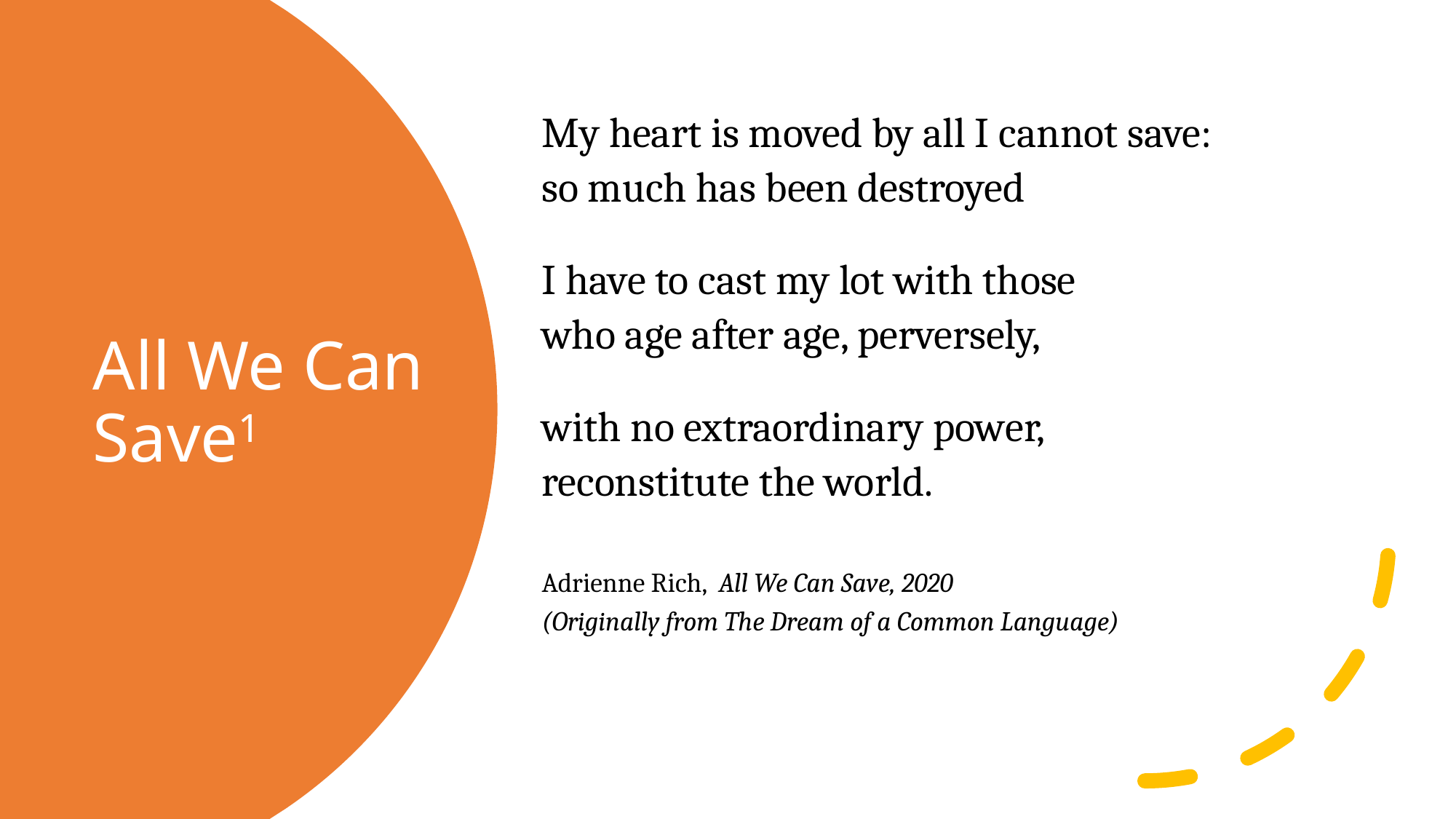

My heart is moved by all I cannot save:
so much has been destroyed
I have to cast my lot with those
who age after age, perversely,
with no extraordinary power,
reconstitute the world.
Adrienne Rich, All We Can Save, 2020
(Originally from The Dream of a Common Language)
# All We Can Save1

## Slide 4
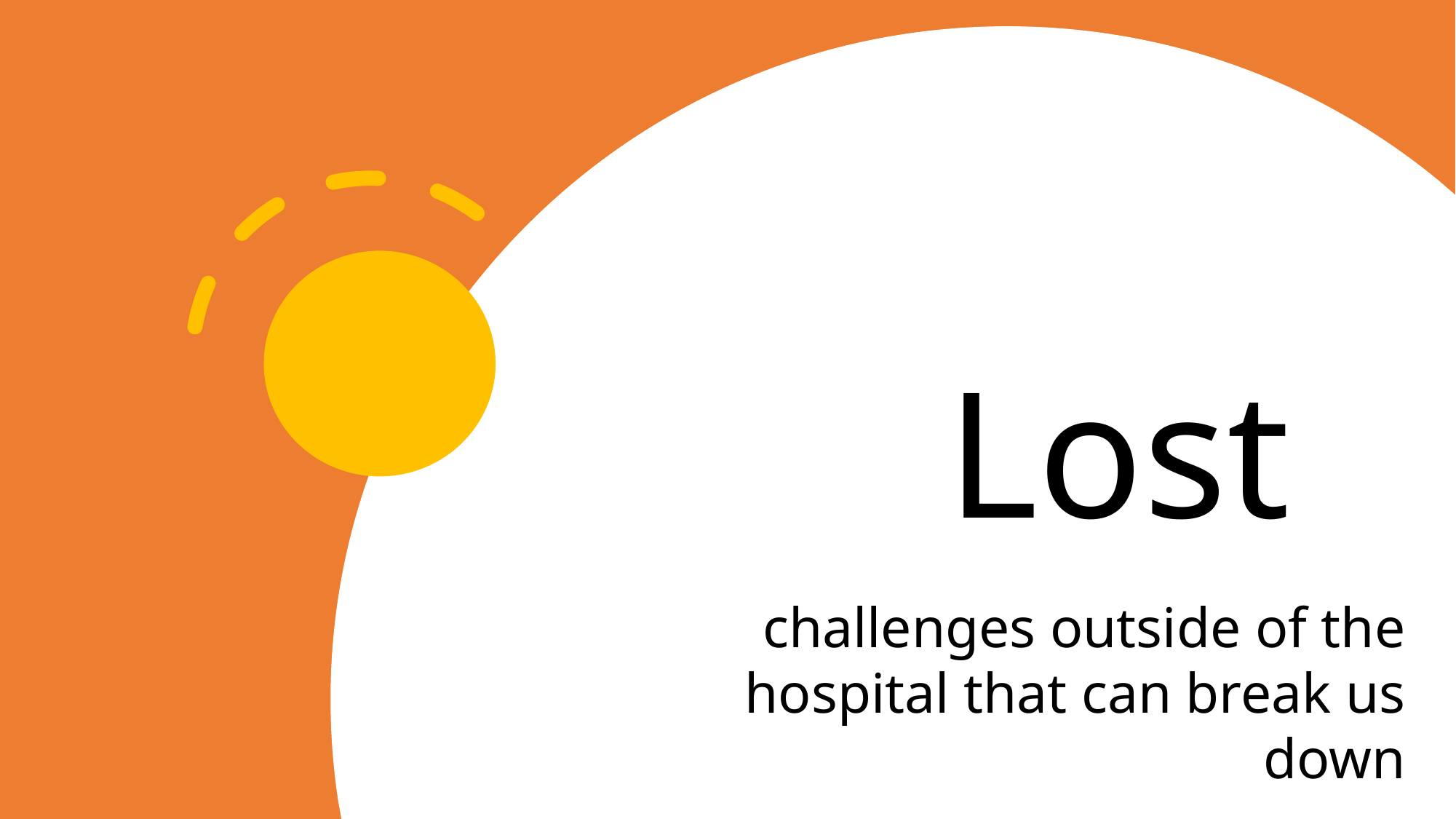

# Lost
challenges outside of the hospital that can break us down

## Slide 5
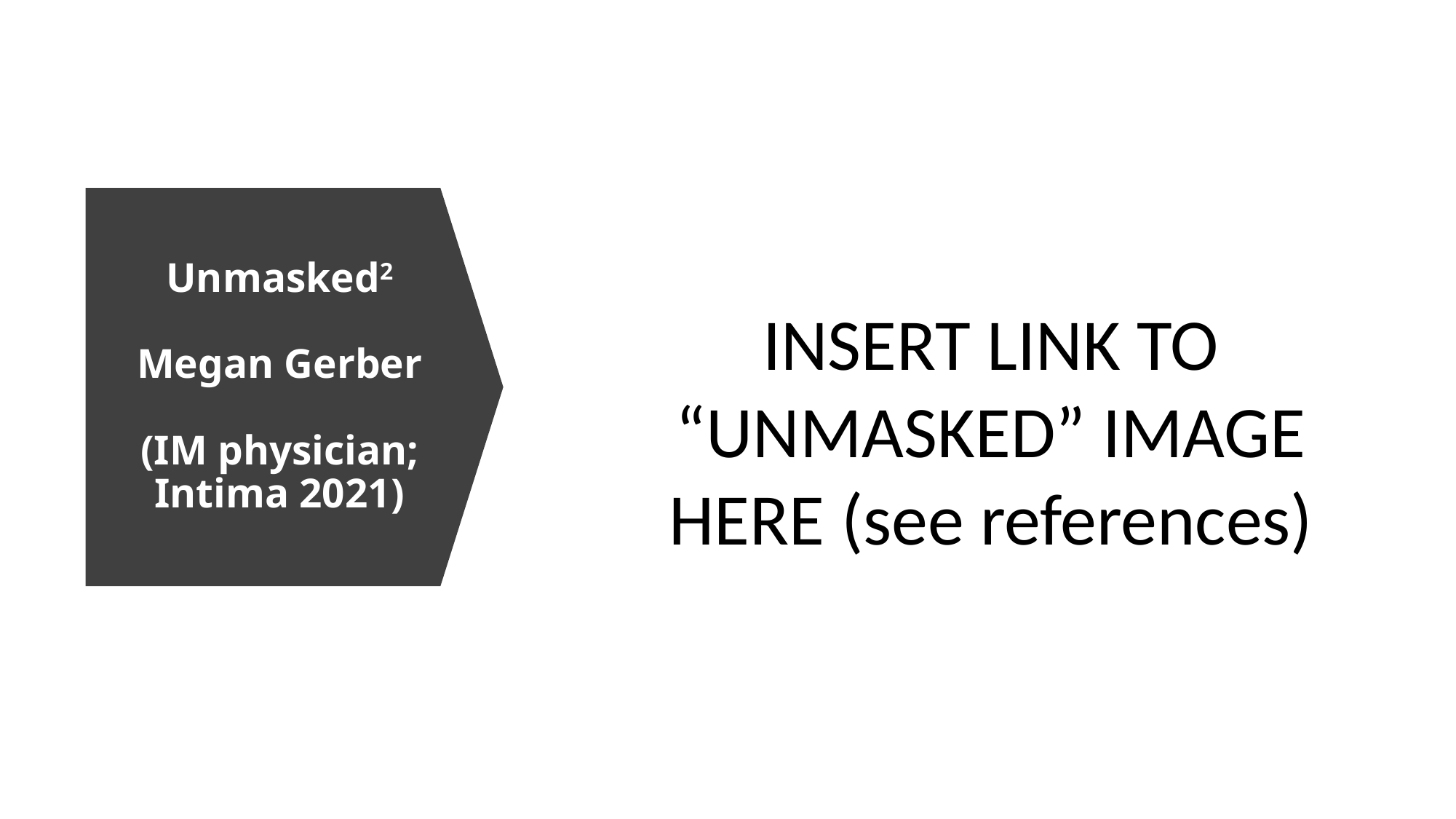

INSERT LINK TO “UNMASKED” IMAGE HERE (see references)
# Unmasked2 Megan Gerber (IM physician; Intima 2021)

## Slide 6
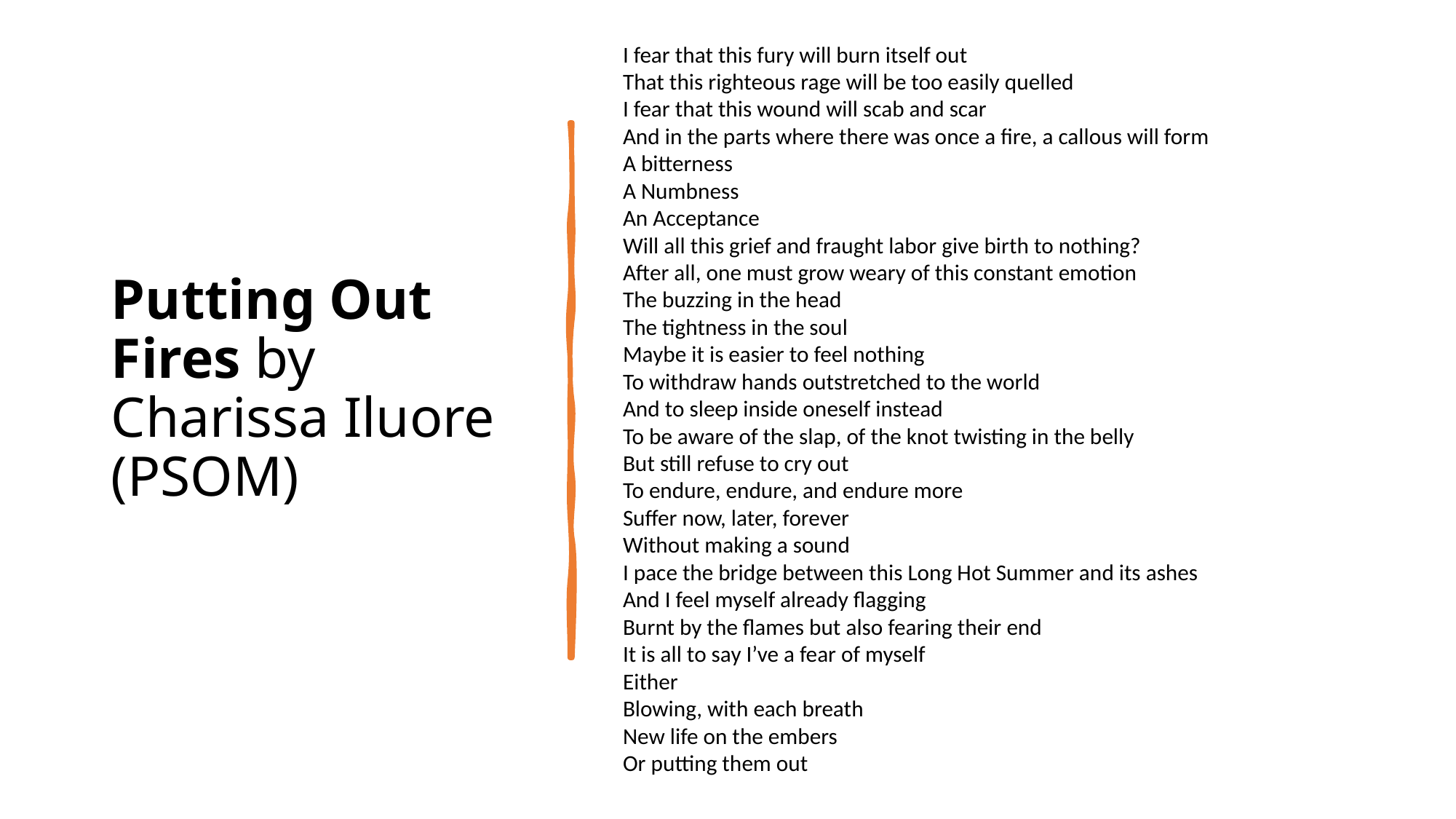

# Putting Out Fires by Charissa Iluore (PSOM)
I fear that this fury will burn itself out
That this righteous rage will be too easily quelled
I fear that this wound will scab and scar
And in the parts where there was once a fire, a callous will form
A bitterness
A Numbness
An Acceptance
Will all this grief and fraught labor give birth to nothing?
After all, one must grow weary of this constant emotion
The buzzing in the head
The tightness in the soul
Maybe it is easier to feel nothing
To withdraw hands outstretched to the world
And to sleep inside oneself instead
To be aware of the slap, of the knot twisting in the belly
But still refuse to cry out
To endure, endure, and endure more
Suffer now, later, forever
Without making a sound
I pace the bridge between this Long Hot Summer and its ashes
And I feel myself already flagging
Burnt by the flames but also fearing their end
It is all to say I’ve a fear of myself
Either
Blowing, with each breath
New life on the embers
Or putting them out

## Slide 7
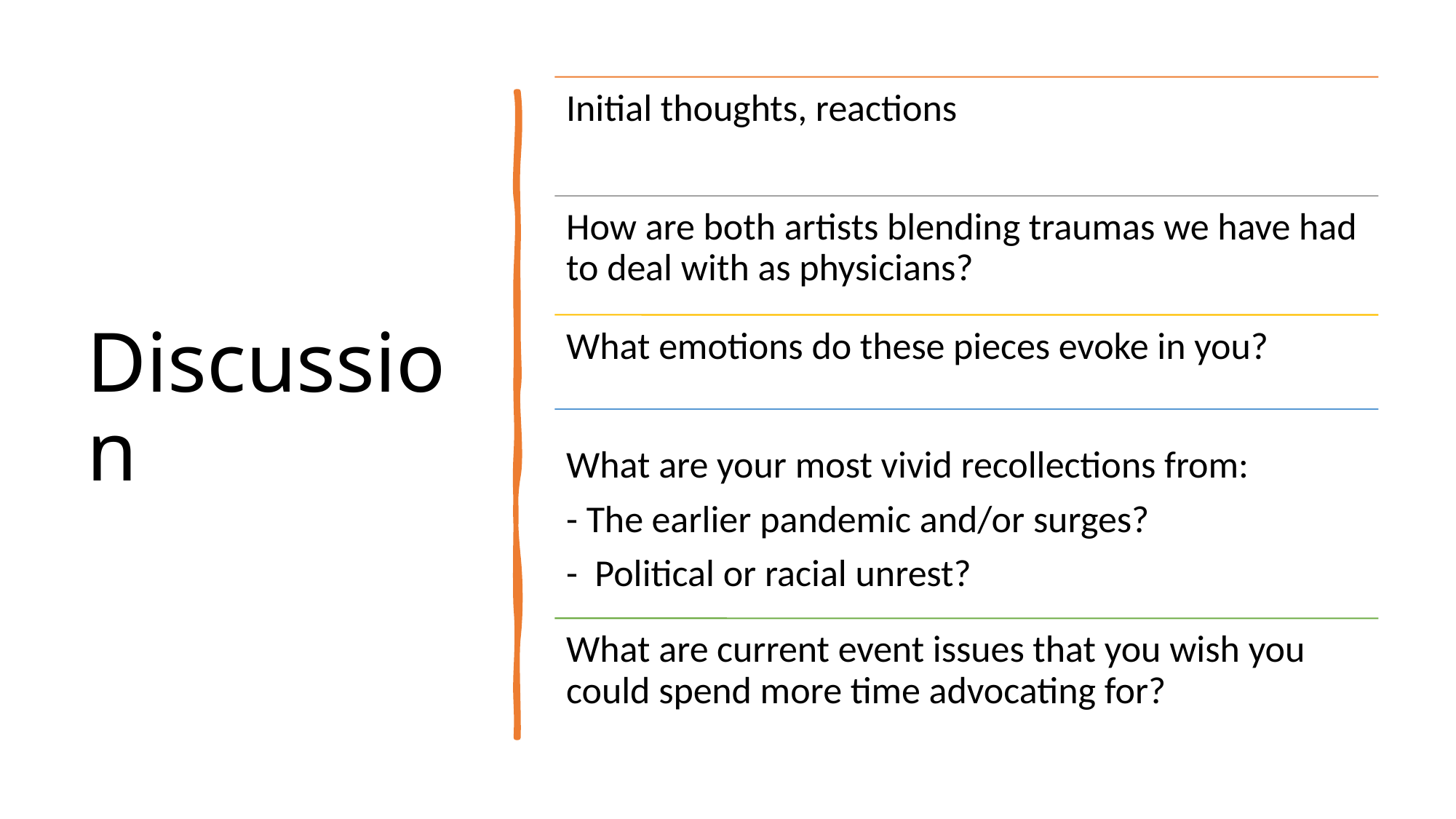

# Discussion

## Slide 8
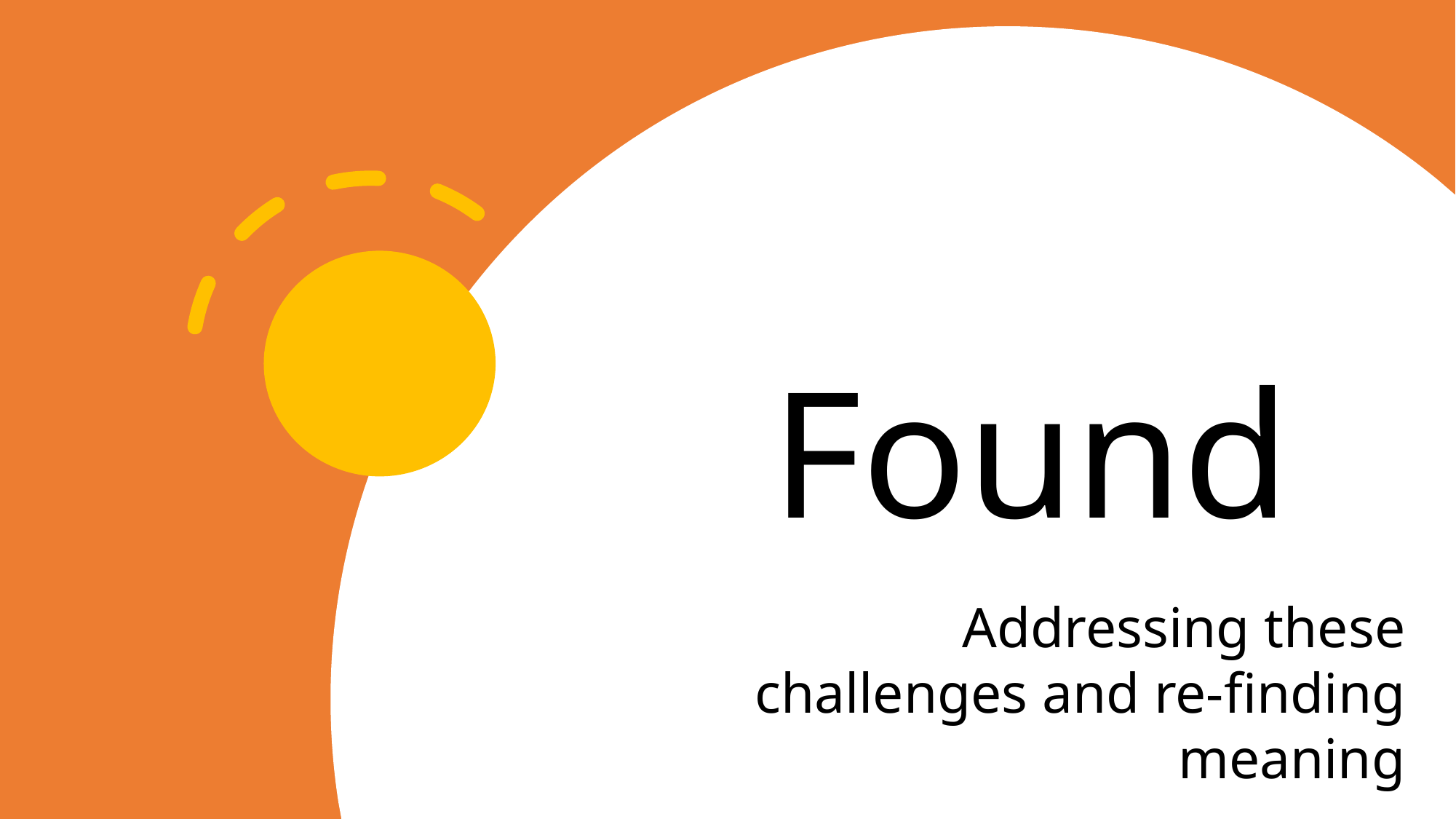

# Found
Addressing these challenges and re-finding meaning

## Slide 9
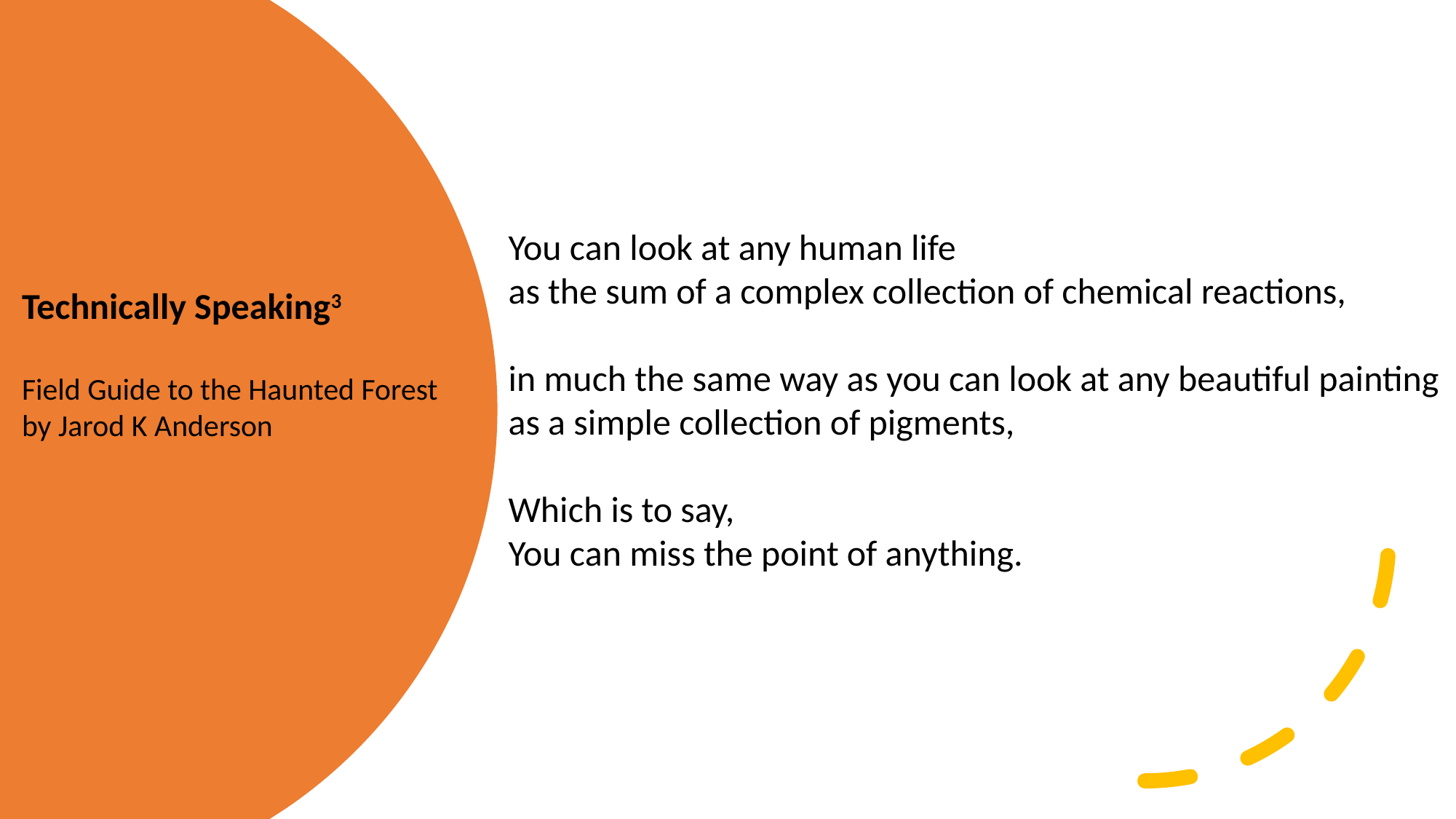

You can look at any human life
as the sum of a complex collection of chemical reactions,
in much the same way as you can look at any beautiful painting
as a simple collection of pigments,
Which is to say,
You can miss the point of anything.
Technically Speaking3
Field Guide to the Haunted Forest
by Jarod K Anderson

## Slide 10
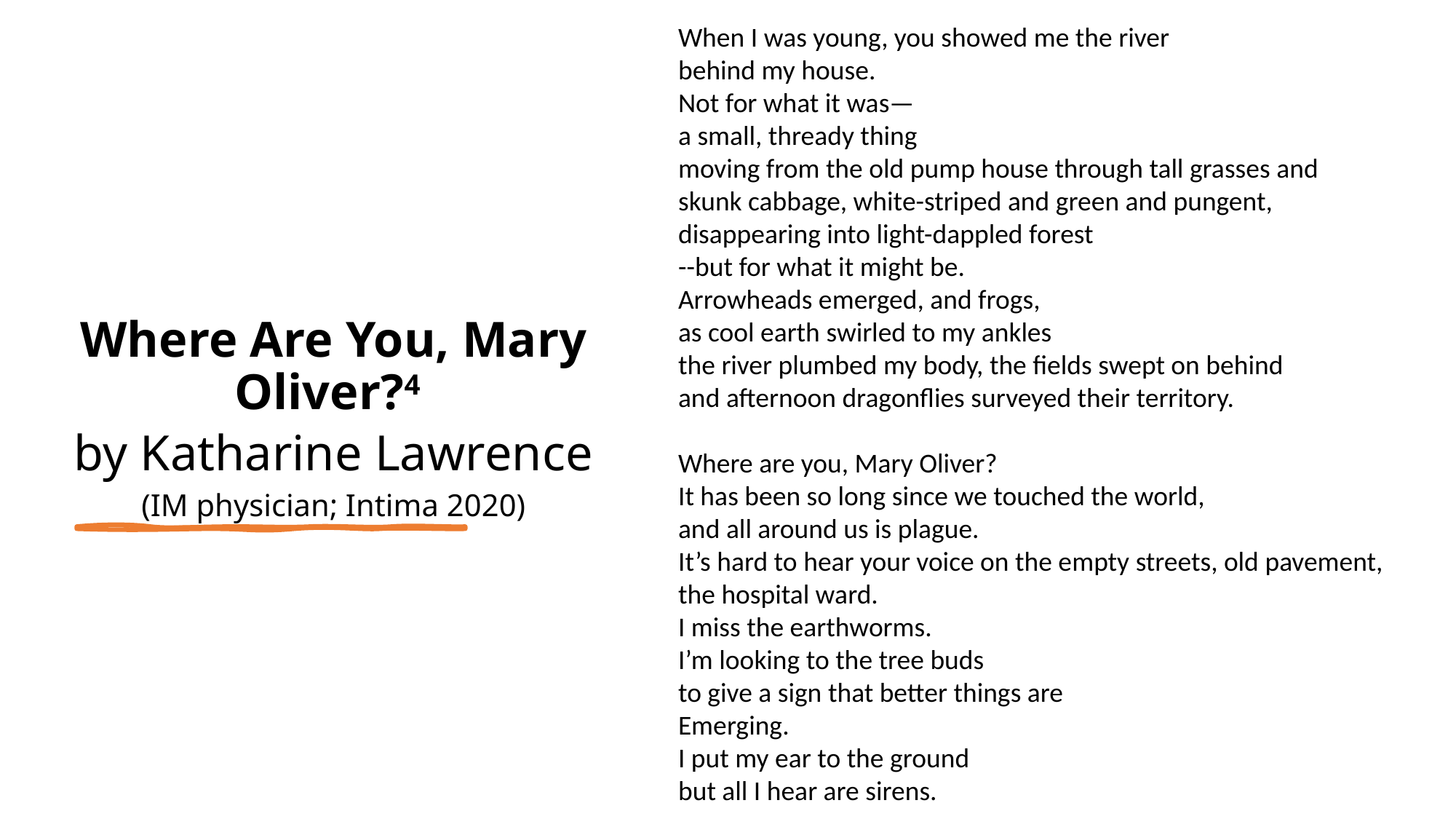

When I was young, you showed me the river
behind my house.
Not for what it was—
a small, thready thing
moving from the old pump house through tall grasses and
skunk cabbage, white-striped and green and pungent,
disappearing into light-dappled forest
--but for what it might be.
Arrowheads emerged, and frogs,
as cool earth swirled to my ankles
the river plumbed my body, the fields swept on behind
and afternoon dragonflies surveyed their territory.
Where are you, Mary Oliver?
It has been so long since we touched the world,
and all around us is plague.
It’s hard to hear your voice on the empty streets, old pavement,
the hospital ward.
I miss the earthworms.
I’m looking to the tree buds
to give a sign that better things are
Emerging.
I put my ear to the ground
but all I hear are sirens.
Where Are You, Mary Oliver?4
by Katharine Lawrence
(IM physician; Intima 2020)

## Slide 11
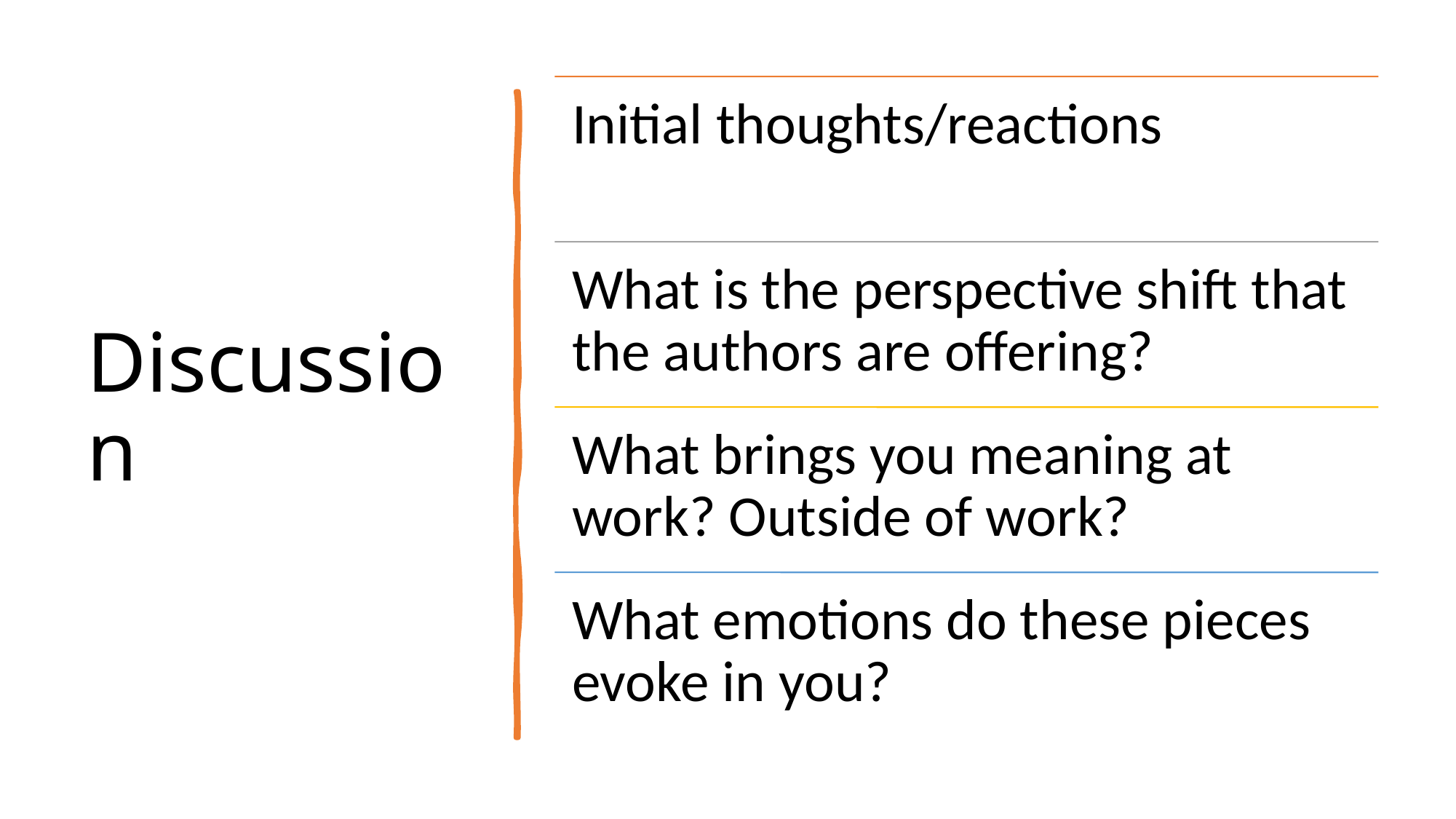

# Discussion

## Slide 12
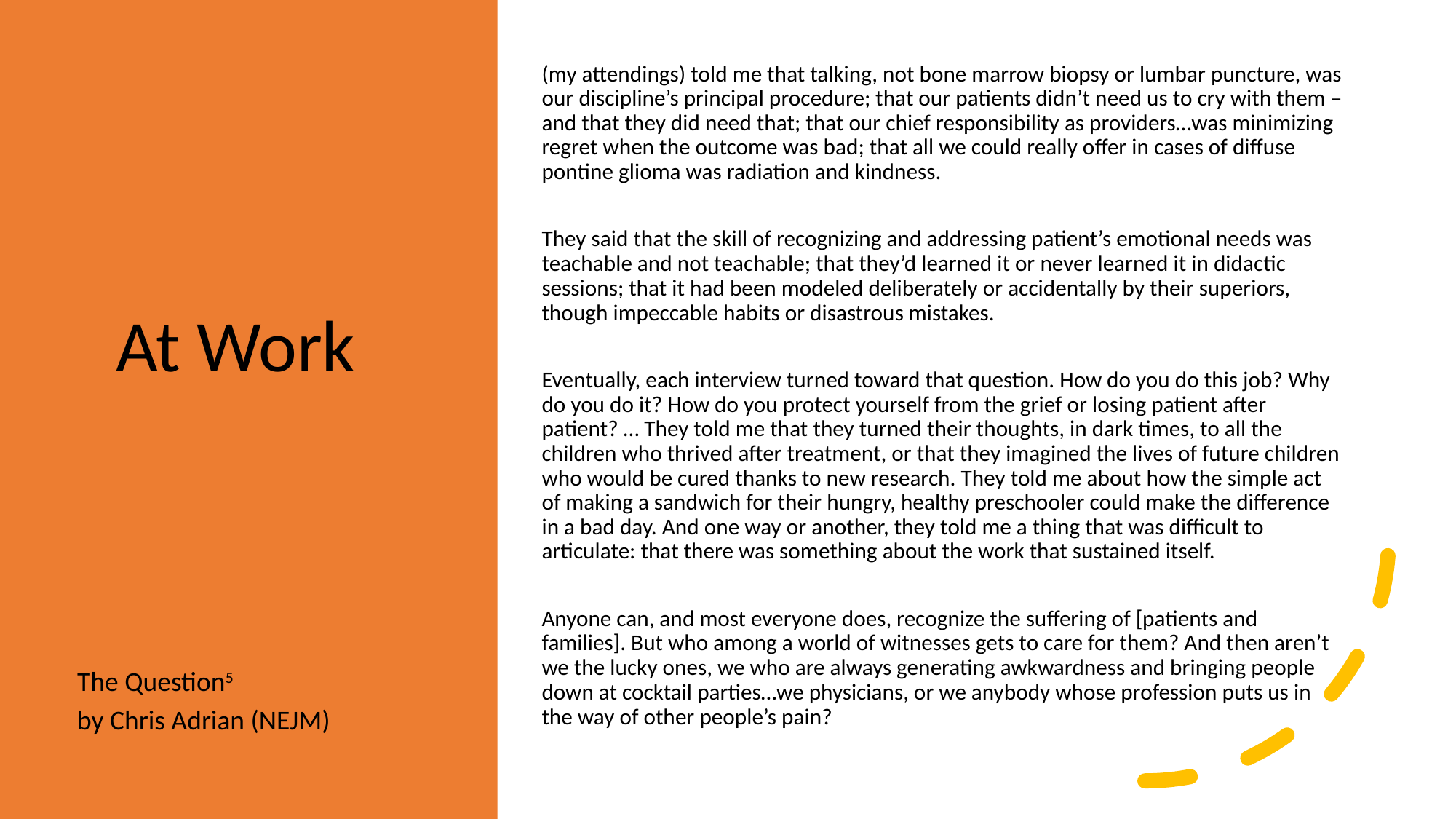

(my attendings) told me that talking, not bone marrow biopsy or lumbar puncture, was our discipline’s principal procedure; that our patients didn’t need us to cry with them – and that they did need that; that our chief responsibility as providers…was minimizing regret when the outcome was bad; that all we could really offer in cases of diffuse pontine glioma was radiation and kindness.
They said that the skill of recognizing and addressing patient’s emotional needs was teachable and not teachable; that they’d learned it or never learned it in didactic sessions; that it had been modeled deliberately or accidentally by their superiors, though impeccable habits or disastrous mistakes.
Eventually, each interview turned toward that question. How do you do this job? Why do you do it? How do you protect yourself from the grief or losing patient after patient? … They told me that they turned their thoughts, in dark times, to all the children who thrived after treatment, or that they imagined the lives of future children who would be cured thanks to new research. They told me about how the simple act of making a sandwich for their hungry, healthy preschooler could make the difference in a bad day. And one way or another, they told me a thing that was difficult to articulate: that there was something about the work that sustained itself.
Anyone can, and most everyone does, recognize the suffering of [patients and families]. But who among a world of witnesses gets to care for them? And then aren’t we the lucky ones, we who are always generating awkwardness and bringing people down at cocktail parties…we physicians, or we anybody whose profession puts us in the way of other people’s pain?
At Work
The Question5
by Chris Adrian (NEJM)

## Slide 13
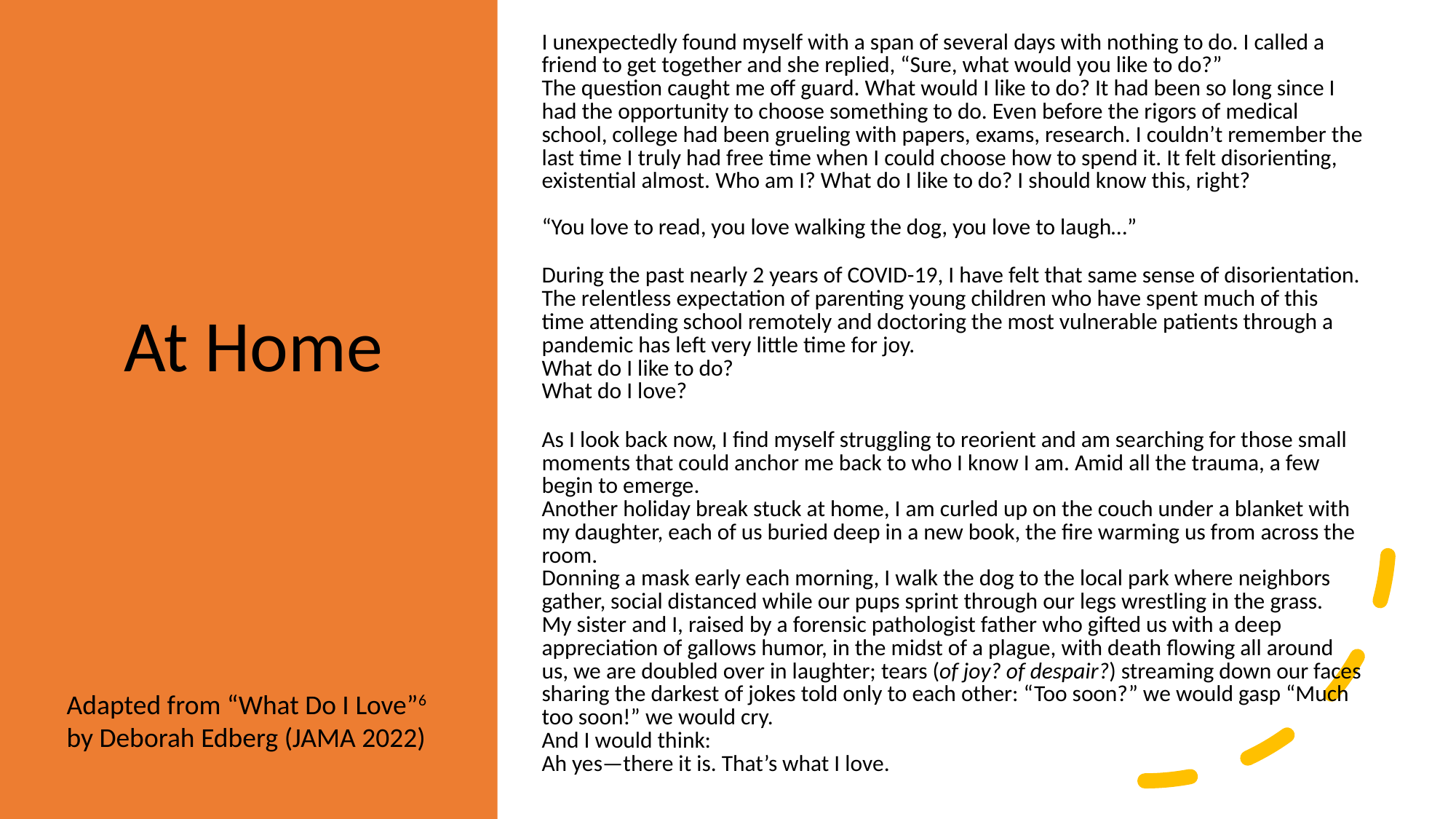

I unexpectedly found myself with a span of several days with nothing to do. I called a friend to get together and she replied, “Sure, what would you like to do?”
The question caught me off guard. What would I like to do? It had been so long since I had the opportunity to choose something to do. Even before the rigors of medical school, college had been grueling with papers, exams, research. I couldn’t remember the last time I truly had free time when I could choose how to spend it. It felt disorienting, existential almost. Who am I? What do I like to do? I should know this, right?
“You love to read, you love walking the dog, you love to laugh…”
During the past nearly 2 years of COVID-19, I have felt that same sense of disorientation. The relentless expectation of parenting young children who have spent much of this time attending school remotely and doctoring the most vulnerable patients through a pandemic has left very little time for joy.
What do I like to do?
What do I love?
As I look back now, I find myself struggling to reorient and am searching for those small moments that could anchor me back to who I know I am. Amid all the trauma, a few begin to emerge.
Another holiday break stuck at home, I am curled up on the couch under a blanket with my daughter, each of us buried deep in a new book, the fire warming us from across the room.
Donning a mask early each morning, I walk the dog to the local park where neighbors gather, social distanced while our pups sprint through our legs wrestling in the grass.
My sister and I, raised by a forensic pathologist father who gifted us with a deep appreciation of gallows humor, in the midst of a plague, with death flowing all around us, we are doubled over in laughter; tears (of joy? of despair?) streaming down our faces sharing the darkest of jokes told only to each other: “Too soon?” we would gasp “Much too soon!” we would cry.
And I would think:
Ah yes—there it is. That’s what I love.
At Home
Adapted from “What Do I Love”6 by Deborah Edberg (JAMA 2022)

## Slide 14
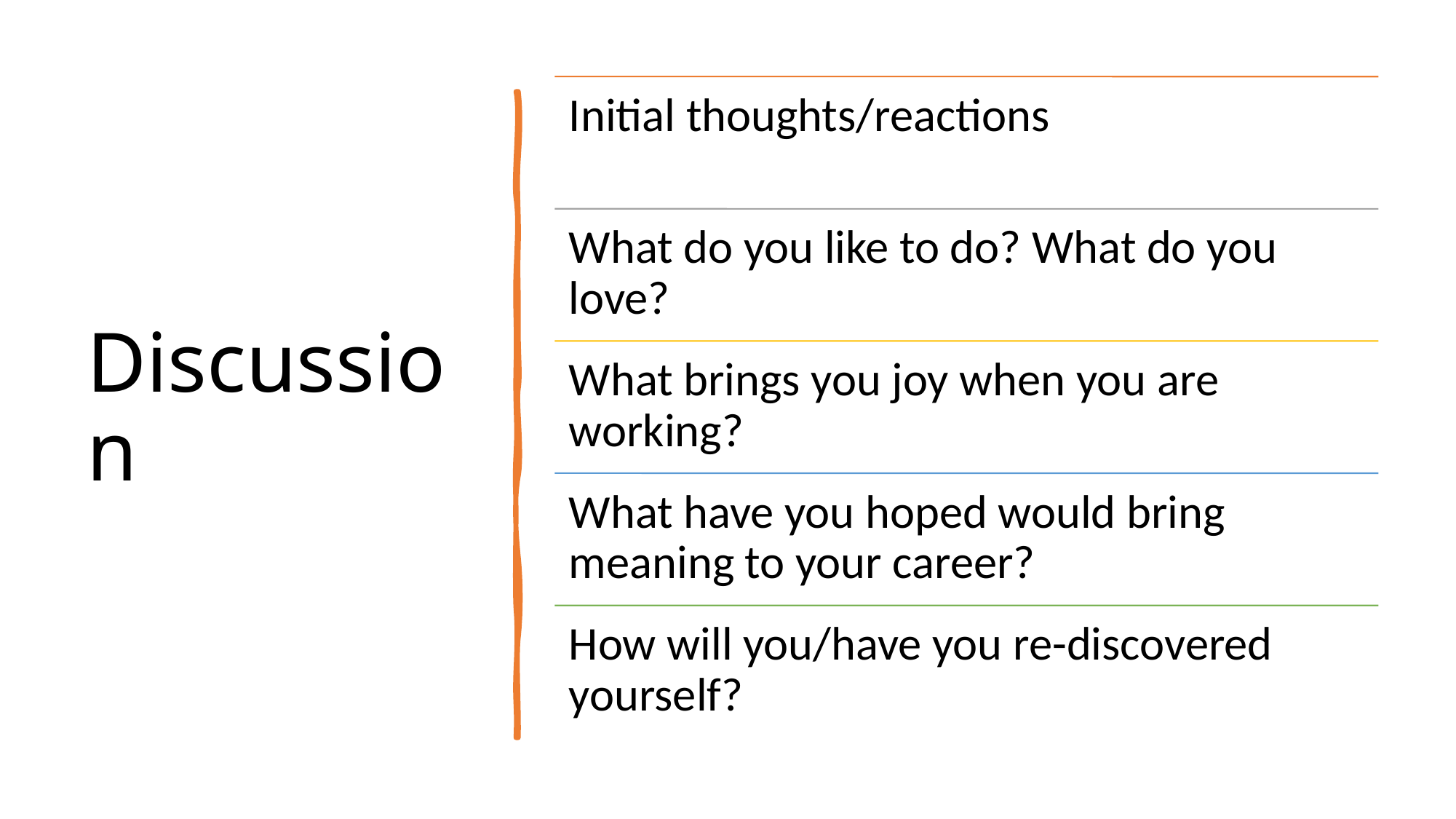

# Discussion

## Slide 15
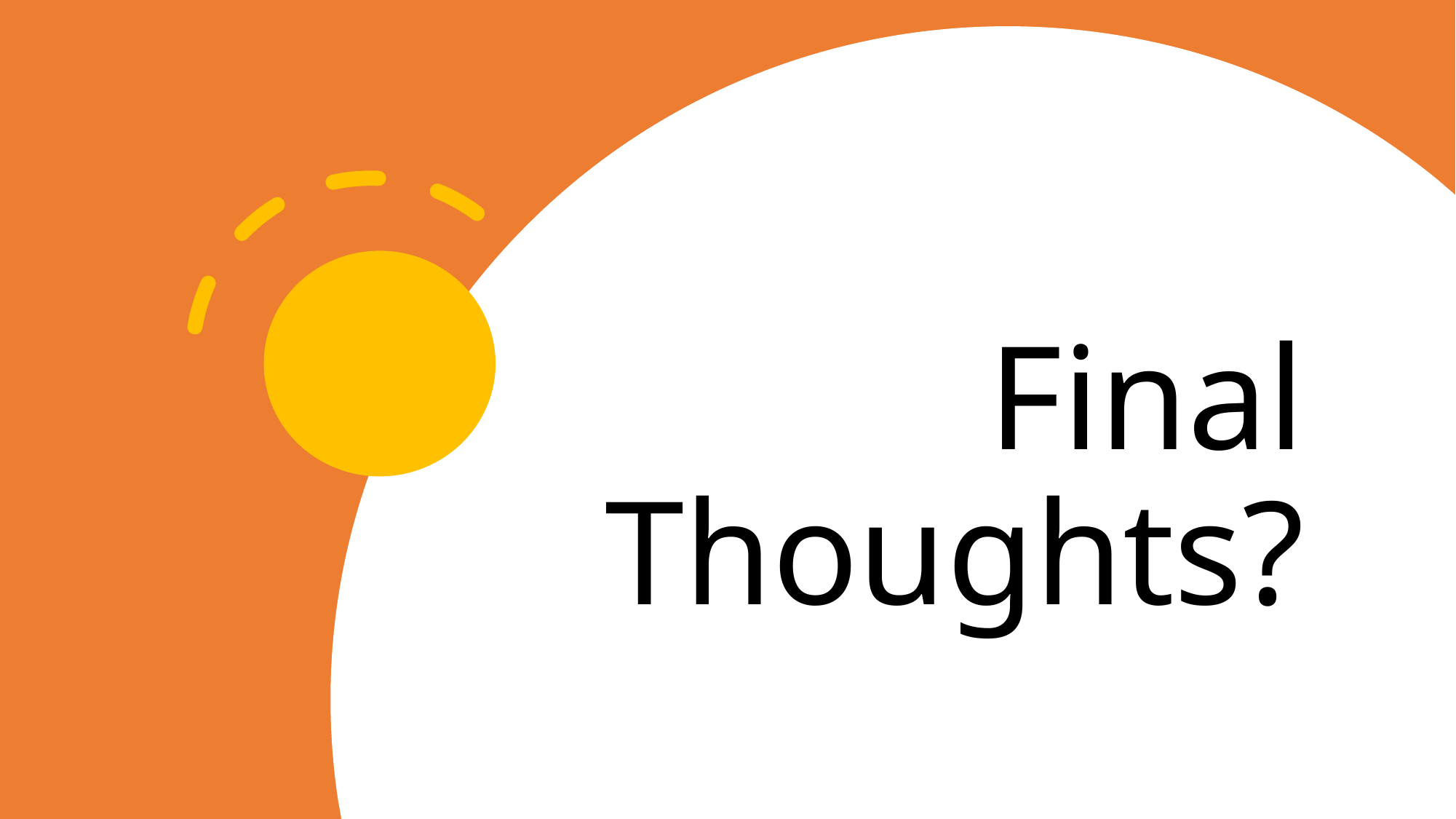

# Final Thoughts?

## Slide 16
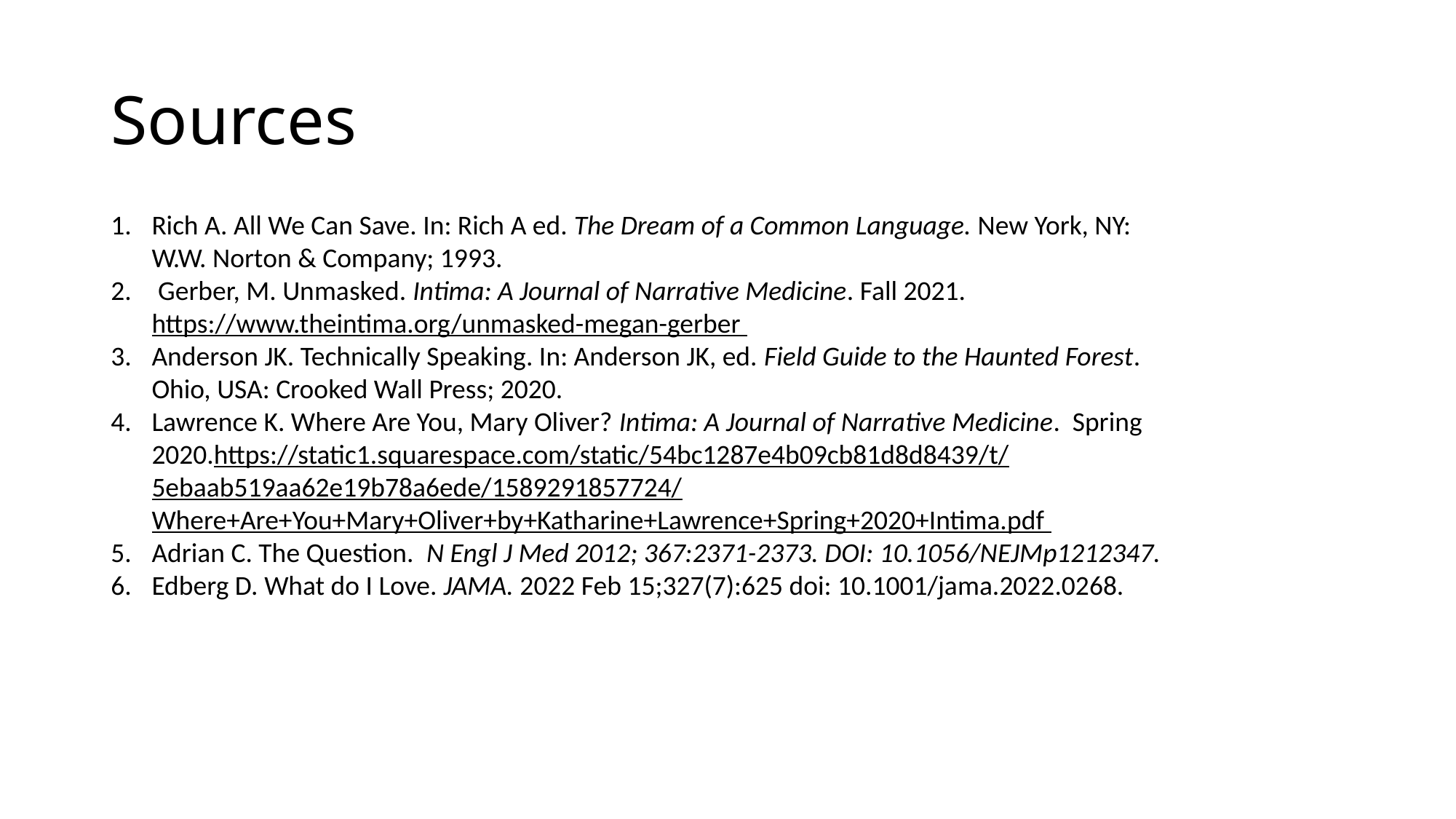

# Sources
Rich A. All We Can Save. In: Rich A ed. The Dream of a Common Language. New York, NY: W.W. Norton & Company; 1993.
 Gerber, M. Unmasked. Intima: A Journal of Narrative Medicine. Fall 2021. https://www.theintima.org/unmasked-megan-gerber
Anderson JK. Technically Speaking. In: Anderson JK, ed. Field Guide to the Haunted Forest. Ohio, USA: Crooked Wall Press; 2020.
Lawrence K. Where Are You, Mary Oliver? Intima: A Journal of Narrative Medicine. Spring 2020.https://static1.squarespace.com/static/54bc1287e4b09cb81d8d8439/t/5ebaab519aa62e19b78a6ede/1589291857724/Where+Are+You+Mary+Oliver+by+Katharine+Lawrence+Spring+2020+Intima.pdf
Adrian C. The Question.  N Engl J Med 2012; 367:2371-2373. DOI: 10.1056/NEJMp1212347.
Edberg D. What do I Love. JAMA. 2022 Feb 15;327(7):625 doi: 10.1001/jama.2022.0268.

## Slide 17
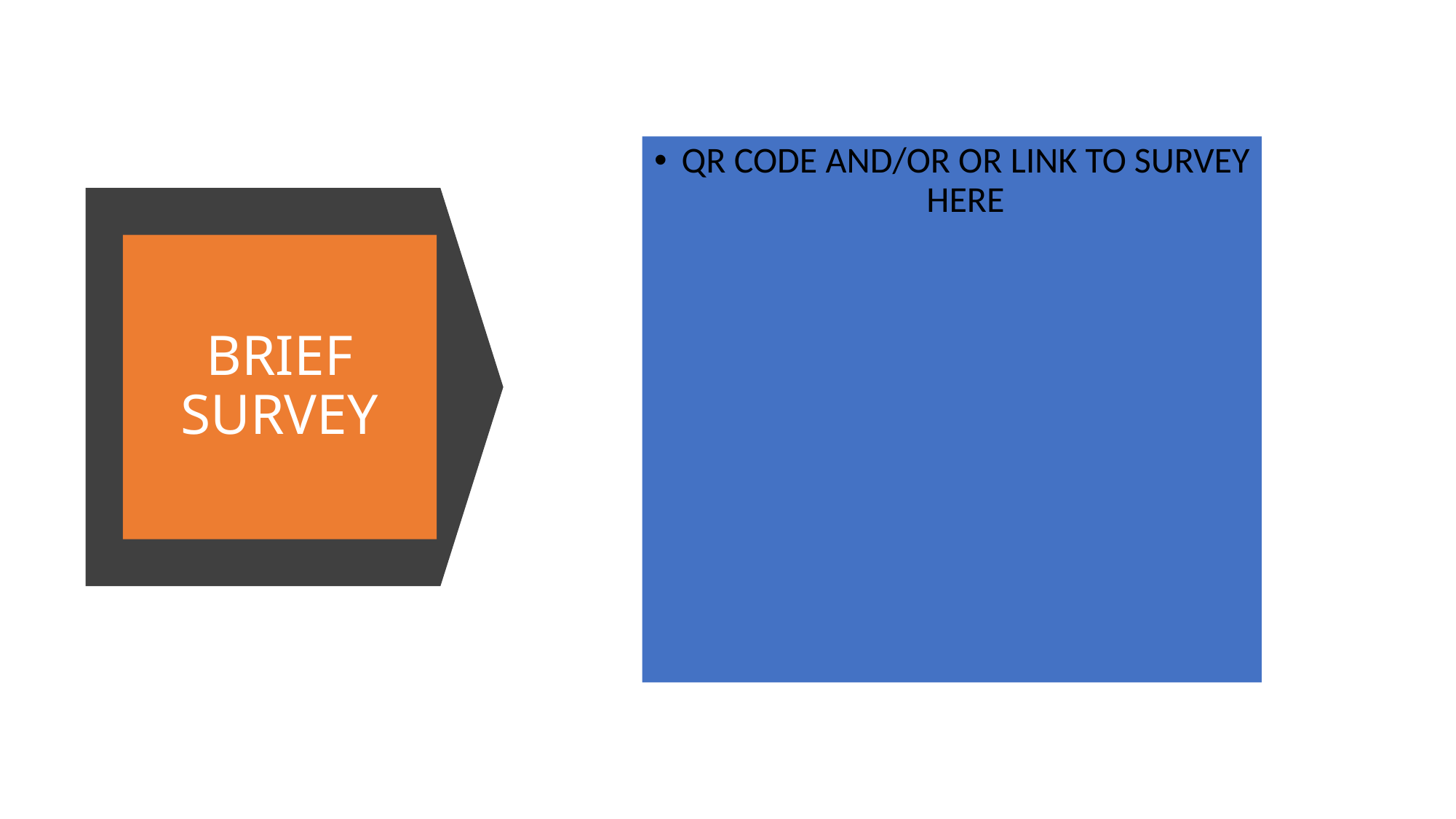

QR CODE AND/OR OR LINK TO SURVEY HERE
# BRIEF SURVEY
