# Supplementary material for: A Resident Narrative Medicine Curriculum to Promote Professional Identity Development: Story-Based Sessions Grounded in Narrative Learning Theory
Source: MedEdPORTAL. 2024 Oct 22;20:11446. doi: 10.15766/mep_2374-8265.11446 (PMC11493853; doi:10.15766/mep_2374-8265.11446)
Supplement: Supplementary file 1 — Facilitator Guide.docxBurnout and Moral Injury.pptxCompassion Fatigue.pptxWorking Through a Pandemic.pptxDifficult Patient.pptxThe New Normal.pptxFinding Meaning.pptxUnpublished Narratives.docxSurvey.docx [file mep_2374-8265.11446-s001.zip › H. Unpublished Narratives.docx]

**Appendix H: Unpublished Narratives**

Session: Burnout and Moral Injury

Title**:** Red Hands

By Michelle Silver, MD

*Written 2019 as an IM Resident, PGY1, Hospital of the University of Pennsylvania*

The room loses focus and monitors become white noise as I fixate on my red hands. I can finally see them after a struggle to peel off the latex gloves which are sticking tightly to my perspiration, as if to shield me from what’s underneath. I am taken aback by their rugged appearance. Raging with heat, trembling, and so slippery that I seem to have lost all dexterity—which becomes apparent as I fumble to secure my pager back to the waistline on my scrubs. My hands have never looked like this before. Or maybe I have never noticed. They are pulsating from the surplus of blood that arrived to support the vigorous activity they just endured. An eerie juxtaposition to the lifeless man in front of me; to my desperate and failed attempt to restore blood flow to his hands. His hands are cold and blue. His hands will never be warm again.

The last thing I remember is falling backward off the stepping stool at this blue man’s bedside, my near fall cushioned by the nurse behind me. The code had just been called to end and my hands release from this man’s chest. My brain signals the rest of my body to do the same, but the adrenaline lags just enough for me to lose my balance while trying to step down. The nurse’s hands on my back ground me, reorient me to place and time: Place – University of Pennsylvania Hospital. Time of death—03:41AM.

I look at the cold, dead, naked man before me. I see the tube that was shoved down his throat moments before in a desperate attempt to oxygenate. I see the drill in his arm from where we were trying to get access to his marrow for faster resuscitation. I see the bright red blood perfectly canvased over his bare, lifeless chest. And I am pissed at the movies and medical dramas for providing hope in a situation like this; for their glorification of this barbaric process that is anything but dignified.

Earlier this evening, I had the hands of a newly minted intern—perfectly manicured and naïve to true exertion. But not anymore. I can taste the heavy breaths that come after repeatedly exerting your weight onto a dying man’s chest. I can hear the acceleration of my heartbeat as the code is finally called to an end and the room goes quiet. I can feel my hands throb, and I can see them in their animalistic form, red and swollen with life.

Session: Compassion Fatigue

Title**:** In the name of patient care?

By Michelle Silver, MD

*Written 2020 as an IM Resident, PGY2, Hospital of the University of Pennsylvania*

Consistent with our morning routine, we move on to the next patient. The senior resident rolls her computer in front of his room. The attending stands next to her in his long white coat. Both look in my direction—my cue to start presenting.

“*Mr. K is a 55 year old with a medical history of vascular and kidney disease, who presented to the ED with difficulty speaking, found to have a large stroke and course complicated by…”* The attending and resident glaze over as I reiterate the same facts that have been repeated for the last two weeks that we’ve been on service; the same facts that have been repeated for the last 20 days since Mr. K arrived, each day slightly less hopeful than the last.

We breeze through his known problems, discussing how he is maxed out on therapy with no changes to the plan. That is, until, at last! Something new. *“This morning labs are notable for a rise in his white blood cell count from 5 to 16, concerning for an emerging infection.”* Suddenly the glazed look disappears, and my attending is in full academic mode. We delve into a debate about where this infection could be. This leads to a lengthy discussion about what part of his body we haven’t tested yet, and we settle on his abdomen. We discuss getting a CT scan to look for a possible abscess to drain.

In the midst of this debate, I pause to look past their shoulders at the emaciated man in the bed behind them. His cheek bones so prominent they could be mistaken for razor blades. He is unresponsive to external stimuli, as I am reminded each morning when I walk into his room to scream “Mr. K!” while forcefully rubbing his sternum, only to be met with the occasional opening of his eyes, on a good day. And his mouth is permanently gaped open as a haunting reminder of the impending rigor mortis. What was not mentioned in the presentation is that Mr. K suffered a stroke so severe, and with so many complications, that he lost the ability to speak; to breath on his own; to acknowledge the people or things in the world around him.

I usually love the academic discussions on rounds, as this dialogue challenges me to apply my medical knowledge to the sick person in front of me. Except this was different. Mr. K had no mental status, and no expectation of recovery. The thought leaves a pit in my stomach.

I think how easy it is to default to this type of reasoning; to find refuge in logic when the emotional weight of this patient’s existence is both taxing and, frankly, illogical, or hard to conceptualize. By discussing an abscess we are caring for this patient in a way we know how and that allows us to move on to the next patient lined up with an equally charged story. We are trying to protect ourselves from further empathic distress, which, for me, paradoxically fuels this distress as I can’t help but wonder –is this really in the name of patient care?

Session: Working through a pandemic

Title**:** The Guilt of Testing Positive

By Jennifer Sun, MD

*Written 2019 as a Med-Peds Resident, PGY1, Hospital of the University of Pennsylvania*

I questioned if I needed to be tested in the first place. I had been working on the night float service admitting patients from the ED– only one who was confirmed COVID positive. Then I developed a mild cough and felt just a little more tired than usual. Out of an abundance of caution, I called my chief, who arranged for testing.

The call came a few days later. She asks me how I’m feeling. “Good, thanks for asking” I reply in my brightest voice. But as the words come out, I hear the tiniest bit of trepidation. She continues, “I’m glad you’re feeling well. I’m calling, however, to let you know that your send-out COVID testing came back positive…”

The facts of what happened made sense, but I start to question myself, wondering if my body betrayed me. I feel fine, right? Sure, I still have a lingering cough and a little tickle in my throat, but allergy season is starting, and I haven’t started my cetirizine yet. And I’m always more tired on night shift rotations. Then again, I did feel short of breath running up those stairs for the code last night. Don’t be ridiculous – I remind myself – I would be short of breath after running upstairs even on a good day. I make a mental note to hit the gym on my next day off. My husband is awake now and listening quietly next to me.

I stay on the phone as we talk about next steps. I start making a mental checklist, the way I always do when I’m overwhelmed. I will need to stay home and call my chiefs to arrange for coverage. I hate calling in my colleagues from jeopardy and the guilt is already starting to creep in – but yes, I can do that. I will need to quarantine at home, including from my husband who is also a physician and scheduled to work this week. One of us will move into the spare bedroom. I will need to list anyone I was within 6 feet of for more than 10 minutes last week so they can also be quarantined and monitored for symptoms.

With every new name the feelings of guilt, horror, and disbelief multiply. I can accept the consequences of my own actions, but the effects extend well beyond myself: The minutes I stood next to my co-resident as we attempted to place an ultrasound-guided IV in a woman with poor access. The well-intentioned hand on the shoulder when I tried to calm a patient writhing in pain during a sickle cell pain crisis. The computer I may have contaminated while trying to place orders during a code. Each of these moments, inconsequential at the time, come back to haunt me.

How ironic it is that a few weeks ago I wanted nothing more than time off to recuperate after a long stretch of inpatient medicine. Now that my wish is granted, all I want is to go back to work and feel useful in the face of this pandemic. Each night, my brain refuses to shut down despite my attempts to numb it with mindless Netflix shows about tiger kings or distract it with YouTube videos of my favorite celebrity puppies. I tell myself it’s the build-up of weeks of sleep deprivation, but my husband and I both know the truth – it’s the beginnings of anxiety and depression rearing its ugly head, feeding hungrily on my thoughts of self-blame and pity.

I slowly start to tell my close friends and family about testing positive for COVID, and I’m overwhelmed by the response. Texts and calls flow in with offers to pick up groceries. Care packages full of soups and easy-to-make meals appear on my doorstop. Gift cards for take-out pop up in my inbox. I feel simultaneously grateful and completely undeserving. At a time when others are lining up at the unemployment office, I continue to get a paycheck and now have a fully stocked pantry.

As I lay in bed, preparing for another sleepless night, I hear my husband’s voice echo from the spare bedroom below: “What is the best thing about Switzerland?” “Chocolate?” I answer. “I don’t know, but the flag is a big plus.” I chuckle and hug my pillow a little tighter.

Session: Working through a pandemic

Title**:** Cheers

By Anne Song, MD

*Written 2020 as an IM Resident, PGY3, Hospital of the University of Pennsylvania*

| You’ve seen the videos  Nurses, doctors lined up  Waiting in the hallway for  The ICU patient  To part the seas  Lights, camera, cheers, applause  Praise  For strength, perseverance, fortitude  For a battle won  For a conqueror, fighter, survivor  But your mom was, too  Your dad  Your brother, your sister  Your child  The strongest person you knew  You were told  They did their best  It wasn’t their fault  The virus was too  Relentless  But the cheers make you wonder  This other person  Was he stronger  Or tougher  Did he love his family more?  Enough to fight  To live  To breathe again  The silence for your family  Was that respect  Or shame? | For weakness, irresolution  For a battle lost  For the defeated  But you were told  It was time  To let go  Was it you?  Did you give up  Too early  If only you had held on  One more day  One more hour  One more breath  Maybe those cheers  Would be for you  Instead  You watch with tears  Envy longing pain grief doubt regret  Questions  Because if you had life  Why would you need  Cheers |
| --- | --- |

Session: Working through a pandemic

Title**:** Skin

By Farah Hussain, MD

*Assistant Professor, Hospital of the University of Pennsylvania*

This life has been rough and ruthless-

exhausted the reserves of our minds, bodies, spirits, souls

Made us fear each other: loved ones and strangers alike,

when we needed them the most.

That was a dirty trick.

A disproportionate viral war against black and brown bodies,

with their skin laid bare in flimsy gowns-

the largest, most beautiful organ

wrapped around knuckles and cheekbones and elbows-

a color inherited of their mothers and fathers and the sun.

We lined them up behind windows to peer through each day;

like a zoo, if you think about it

watched them eat and sleep and counted their breaths;

learned them by their numbers: 20L, 40%.

Not their names.

I am sorry I did not know you for you,

just your disease.

Your black and brown bodies deserved more than me.

Sessions: The New Normal: residency two years into a pandemic, and Finding Meaning in Medicine

Title**:** Putting out Fires

By Charissa Iluore

*Written 2021 as medical student, Hospital of the University of Pennsylvania, Perelman School of Medicine*

I fear that this fury will burn itself out

That this righteous rage will be too easily quelled

I fear that this wound will scab and scar

And in the parts where there was once a fire, a callous will form

A bitterness

A Numbness

An Acceptance

Will all this grief and fraught labor give birth to nothing?

After all, one must grow weary of this constant emotion

The buzzing in the head

The tightness in the soul

Maybe it is easier to feel nothing

To withdraw hands outstretched to the world

And to sleep inside oneself instead

To be aware of the slap, of the knot twisting in the belly

But still refuse to cry out

To endure, endure, and endure more

Suffer now, later, forever

Without making a sound

I pace the bridge between this Long Hot Summer and its ashes

And I feel myself already flagging

Burnt by the flames but also fearing their end

It is all to say I’ve a fear of myself

Either

Blowing, with each breath

New life on the embers

Or putting them out
