# Supplementary material for: A Resident Narrative Medicine Curriculum to Promote Professional Identity Development: Story-Based Sessions Grounded in Narrative Learning Theory
Source: MedEdPORTAL. 2024 Oct 22;20:11446. doi: 10.15766/mep_2374-8265.11446 (PMC11493853; doi:10.15766/mep_2374-8265.11446)
Supplement: Supplementary file 1 — Facilitator Guide.docxBurnout and Moral Injury.pptxCompassion Fatigue.pptxWorking Through a Pandemic.pptxDifficult Patient.pptxThe New Normal.pptxFinding Meaning.pptxUnpublished Narratives.docxSurvey.docx [file mep_2374-8265.11446-s001.zip › I. Survey.docx]

**Demographic Data**

What track are you in:

- Primary care, categorical, med-peds, research pathway

What PGY-year are you

- 1, 2, 3, 4, 5+

Please provide your last Initial and last 4 digits to your cellphone

**As a result of today’s narrative medicine session, ______** is**:**

[significantly worse, somewhat worsened, unchanged, somewhat improved, greatly improved]

1. My level of empathy toward patients:
2. The connection I feel with my co-residents:
3. My ability to articulate difficult patient experiences with colleagues or team members:
4. The level of satisfaction I have with my job:
5. My current wellness:
6. My understanding of personal values in the context of my job:

**On a scale of 1-5, I found today’s narrative medicine session:**

[ 1 (strongly disagree) 2, 3, 4, 5 (strongly agree) ]

1. Personally valuable
2. Enjoyable
3. Will positively impact my clinical interactions with patients

**How useful were the following parts of the session**

[1 not at all 🡪 5 extremely useful]

1. Reading/Media
2. Group Discussion
3. Writing
4. Sharing with partner
5. Overall

**Open-Ended Questions**

1. If someone asked you why these sessions are part of your residency training, what would you say (REQUIRED)
2. What is your overall impression of today’s narrative medicine session? (OPTIONAL)
3. Suggested areas for improvement (OPTIONAL)
