# Supplementary material for: A Resident Narrative Medicine Curriculum to Promote Professional Identity Development: Story-Based Sessions Grounded in Narrative Learning Theory
Source: MedEdPORTAL. 2024 Oct 22;20:11446. doi: 10.15766/mep_2374-8265.11446 (PMC11493853; doi:10.15766/mep_2374-8265.11446)
Supplement: Supplementary file 1 — Facilitator Guide.docxBurnout and Moral Injury.pptxCompassion Fatigue.pptxWorking Through a Pandemic.pptxDifficult Patient.pptxThe New Normal.pptxFinding Meaning.pptxUnpublished Narratives.docxSurvey.docx [file mep_2374-8265.11446-s001.zip › A. Facilitator Guide.docx]

**Appendix A: Facilitator Guide for Leading Narrative Medicine Sessions**

**Best Practices:**

1. No pre-assigned work for the sessions
2. Structure sessions within dedicated didactic time, so they are built-in to residents’ schedules
3. Identify the theme at the beginning of each session to orient residents and establish expectations, including the promotion of a safe, confidential space
4. Encourage participants to read literary works out loud to the group
5. Include clear discussion questions that will help participants relate the literary prompt to their own experiences in medical training
6. Sessions should be facilitated by a trusted core faculty member who the residents may work with clinically but serves in a non-evaluative position; consider including an additional, peer-facilitator more directly connected to the resident experience
7. Faculty leader is connected to the appropriate resources to refer participants who bring up concerning responses and/or may need extended time to debrief
8. Program leadership places emphasis on the importance of narrative medicine sessions

**Session Structure:**

We recommended 60-90 minutes for each Narrative Medicine Session. Within each session, when a literary or art source is presented allow a minimum of 3-5 minutes of self-reflection, with option to write down their thoughts, prior to opening up to group discussion. During this time, ensure the discussion questions are displayed to promote such reflection.
