# Supplementary material for: A Resident Narrative Medicine Curriculum to Promote Professional Identity Development: Story-Based Sessions Grounded in Narrative Learning Theory
Source: MedEdPORTAL. 2024 Oct 22;20:11446. doi: 10.15766/mep_2374-8265.11446 (PMC11493853; doi:10.15766/mep_2374-8265.11446)
Supplement: Supplementary file 1 — Facilitator Guide.docxBurnout and Moral Injury.pptxCompassion Fatigue.pptxWorking Through a Pandemic.pptxDifficult Patient.pptxThe New Normal.pptxFinding Meaning.pptxUnpublished Narratives.docxSurvey.docx [file mep_2374-8265.11446-s001.zip › B. Burnout and Moral Injury.pptx]

## Slide 1
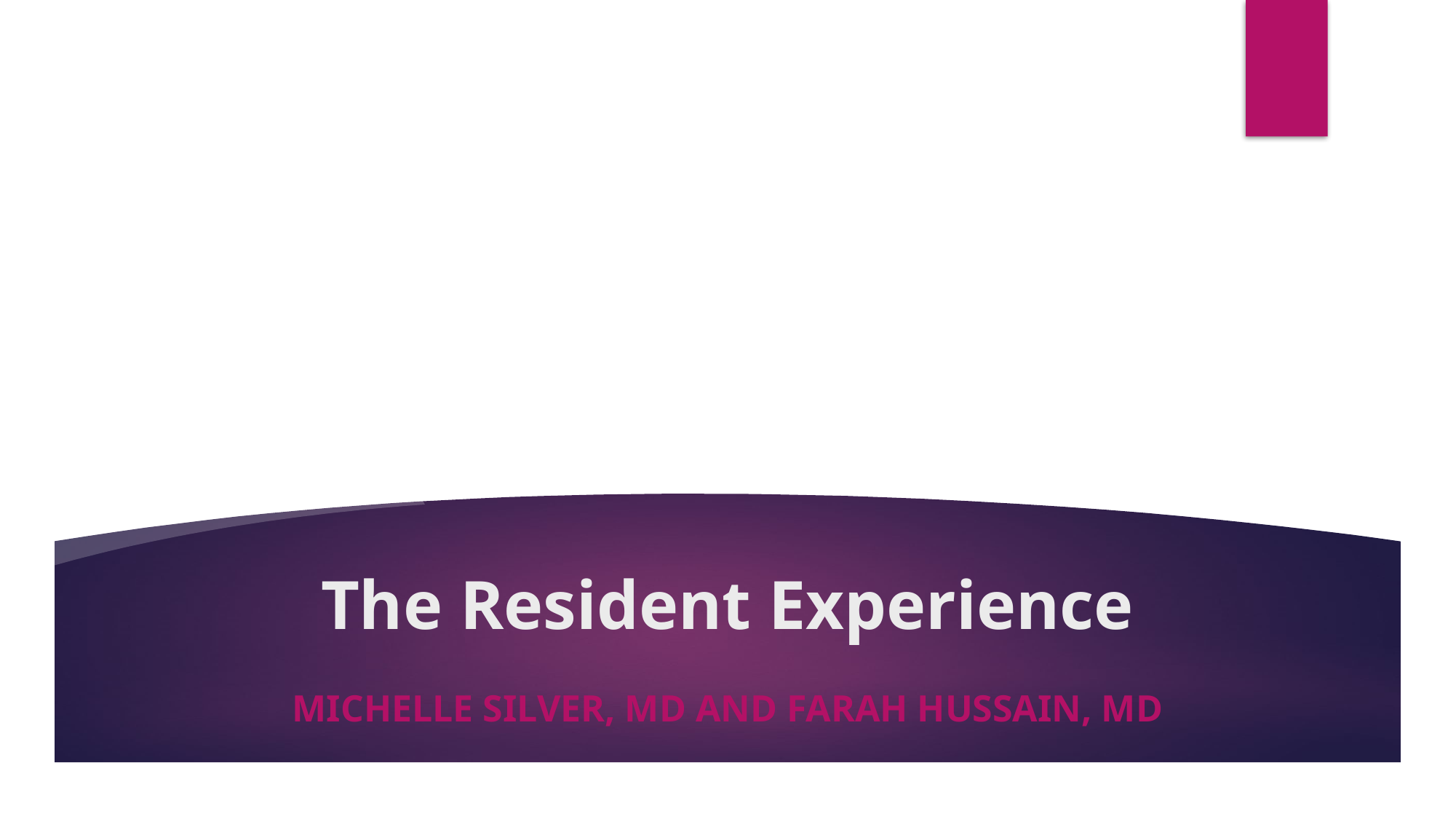

# The Resident Experience
Michelle Silver, MD and Farah Hussain, MD

## Slide 2
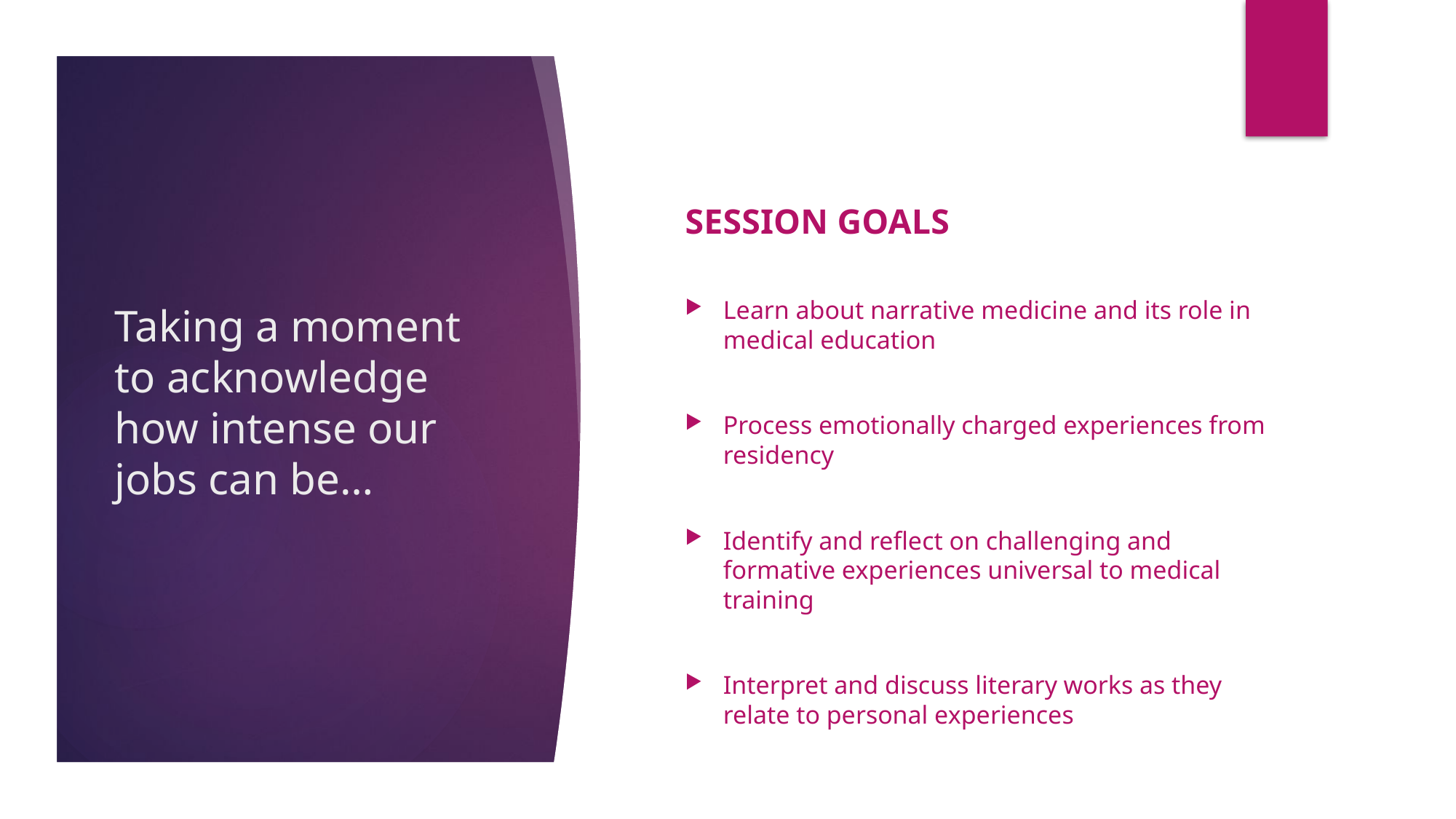

# Taking a moment to acknowledge how intense our jobs can be…
SESSION GOALS
Learn about narrative medicine and its role in medical education
Process emotionally charged experiences from residency
Identify and reflect on challenging and formative experiences universal to medical training
Interpret and discuss literary works as they relate to personal experiences

## Slide 3
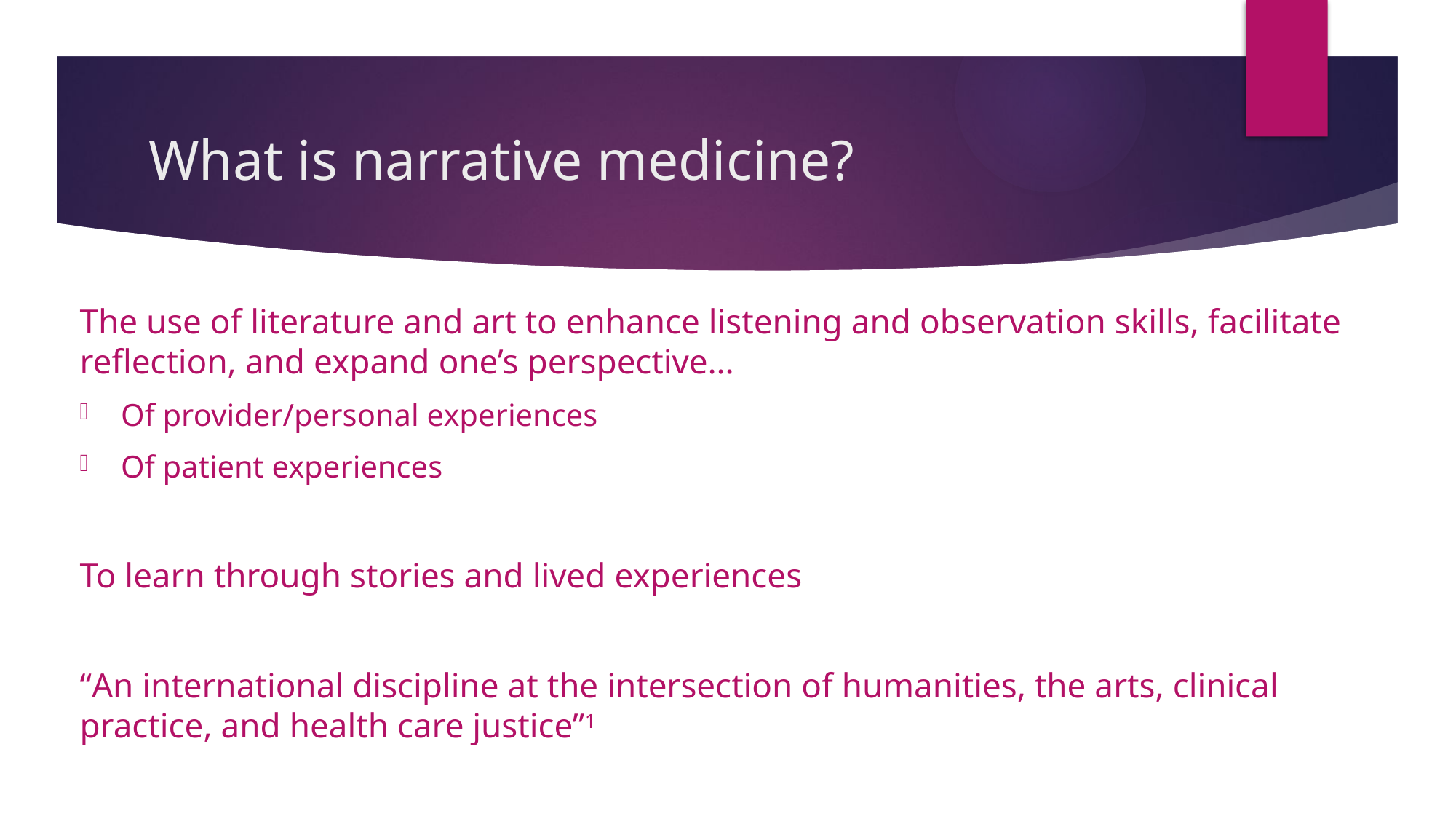

# What is narrative medicine?
The use of literature and art to enhance listening and observation skills, facilitate reflection, and expand one’s perspective…
Of provider/personal experiences
Of patient experiences
To learn through stories and lived experiences
“An international discipline at the intersection of humanities, the arts, clinical practice, and health care justice”1

## Slide 4
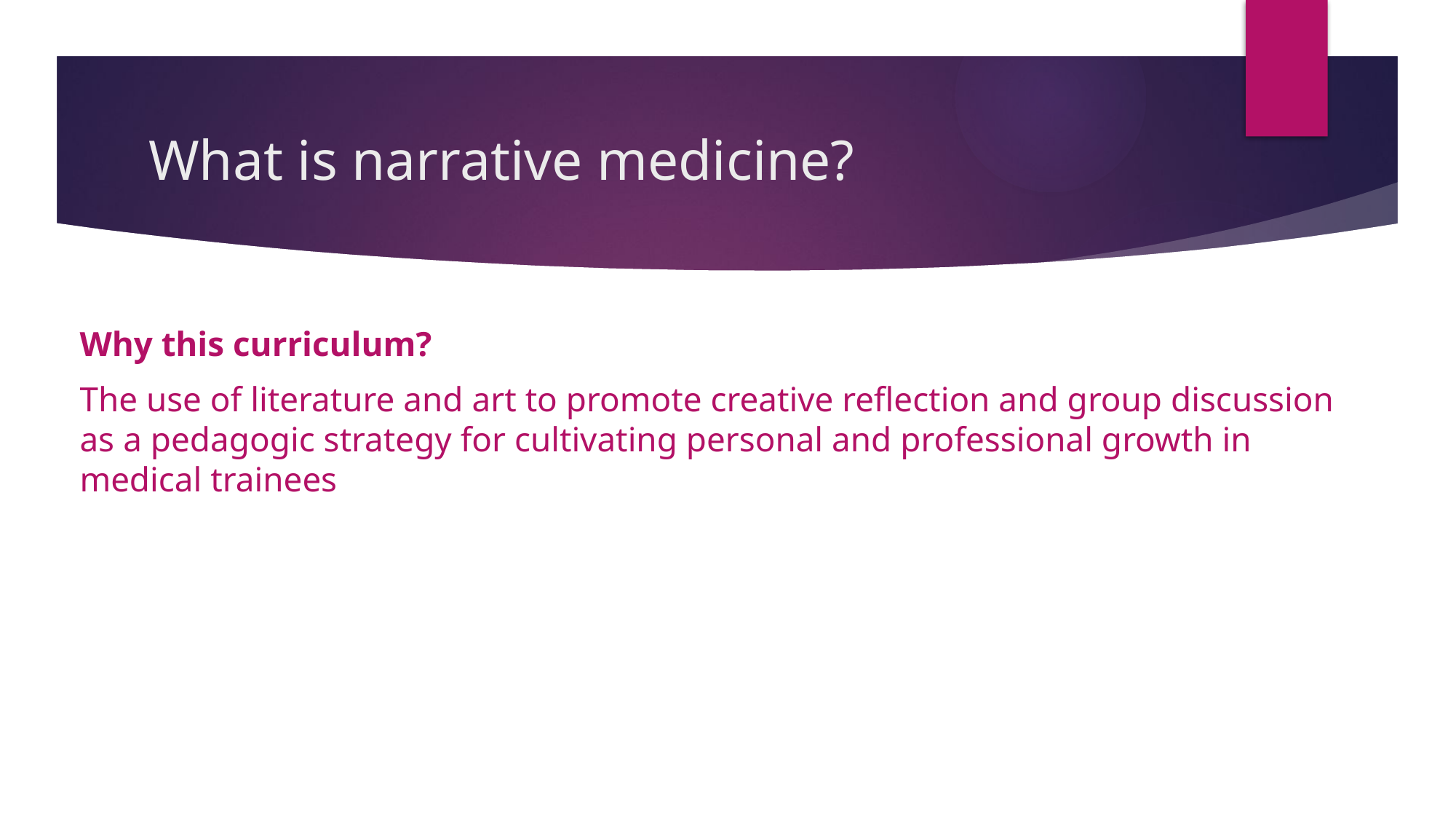

# What is narrative medicine?
Why this curriculum?
The use of literature and art to promote creative reflection and group discussion as a pedagogic strategy for cultivating personal and professional growth in medical trainees

## Slide 5
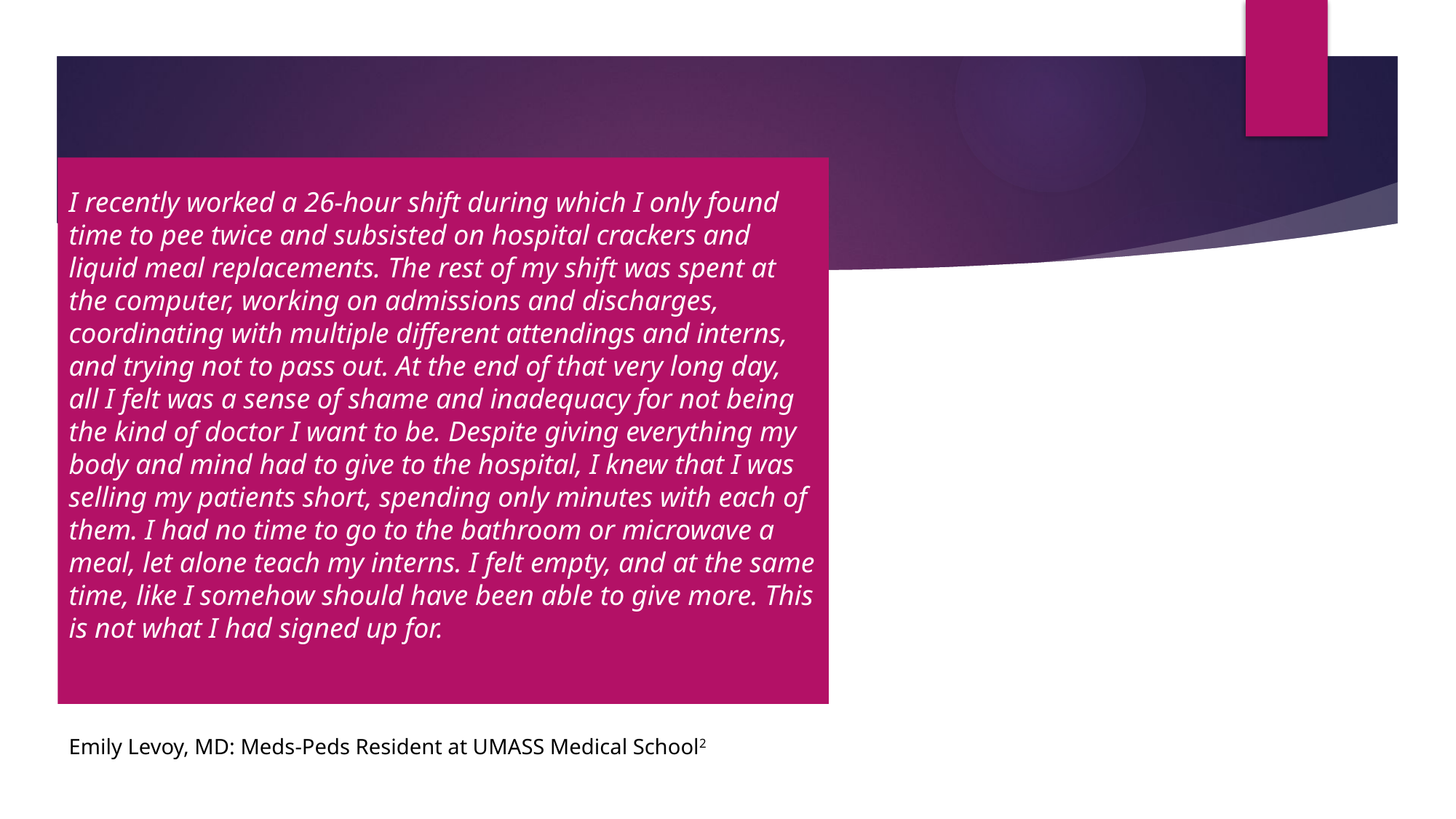

# I recently worked a 26-hour shift during which I only found time to pee twice and subsisted on hospital crackers and liquid meal replacements. The rest of my shift was spent at the computer, working on admissions and discharges, coordinating with multiple different attendings and interns, and trying not to pass out. At the end of that very long day, all I felt was a sense of shame and inadequacy for not being the kind of doctor I want to be. Despite giving everything my body and mind had to give to the hospital, I knew that I was selling my patients short, spending only minutes with each of them. I had no time to go to the bathroom or microwave a meal, let alone teach my interns. I felt empty, and at the same time, like I somehow should have been able to give more. This is not what I had signed up for.
Emily Levoy, MD: Meds-Peds Resident at UMASS Medical School2

## Slide 6
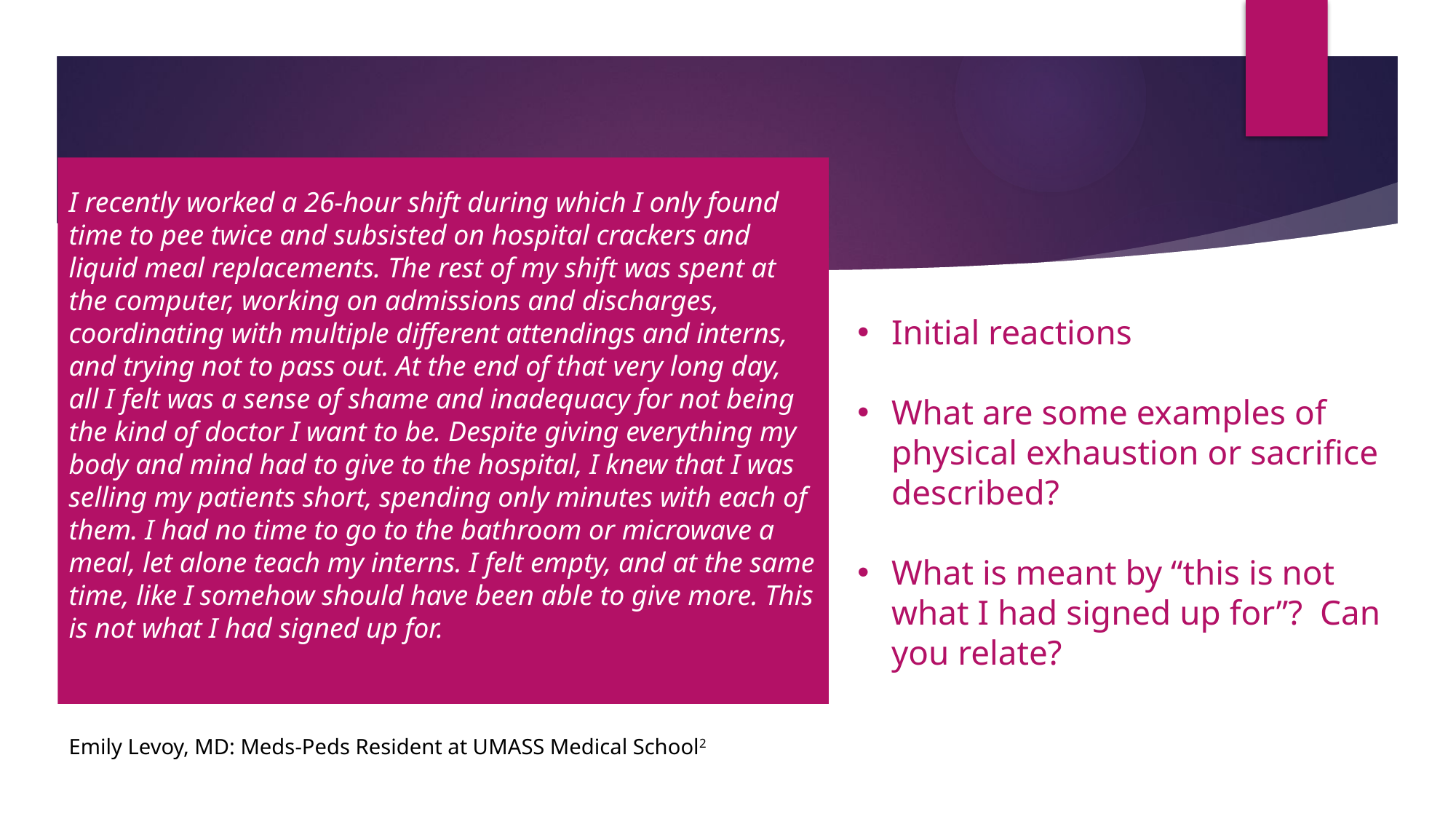

# I recently worked a 26-hour shift during which I only found time to pee twice and subsisted on hospital crackers and liquid meal replacements. The rest of my shift was spent at the computer, working on admissions and discharges, coordinating with multiple different attendings and interns, and trying not to pass out. At the end of that very long day, all I felt was a sense of shame and inadequacy for not being the kind of doctor I want to be. Despite giving everything my body and mind had to give to the hospital, I knew that I was selling my patients short, spending only minutes with each of them. I had no time to go to the bathroom or microwave a meal, let alone teach my interns. I felt empty, and at the same time, like I somehow should have been able to give more. This is not what I had signed up for.
Initial reactions
What are some examples of physical exhaustion or sacrifice described?
What is meant by “this is not what I had signed up for”? Can you relate?
Emily Levoy, MD: Meds-Peds Resident at UMASS Medical School2

## Slide 7
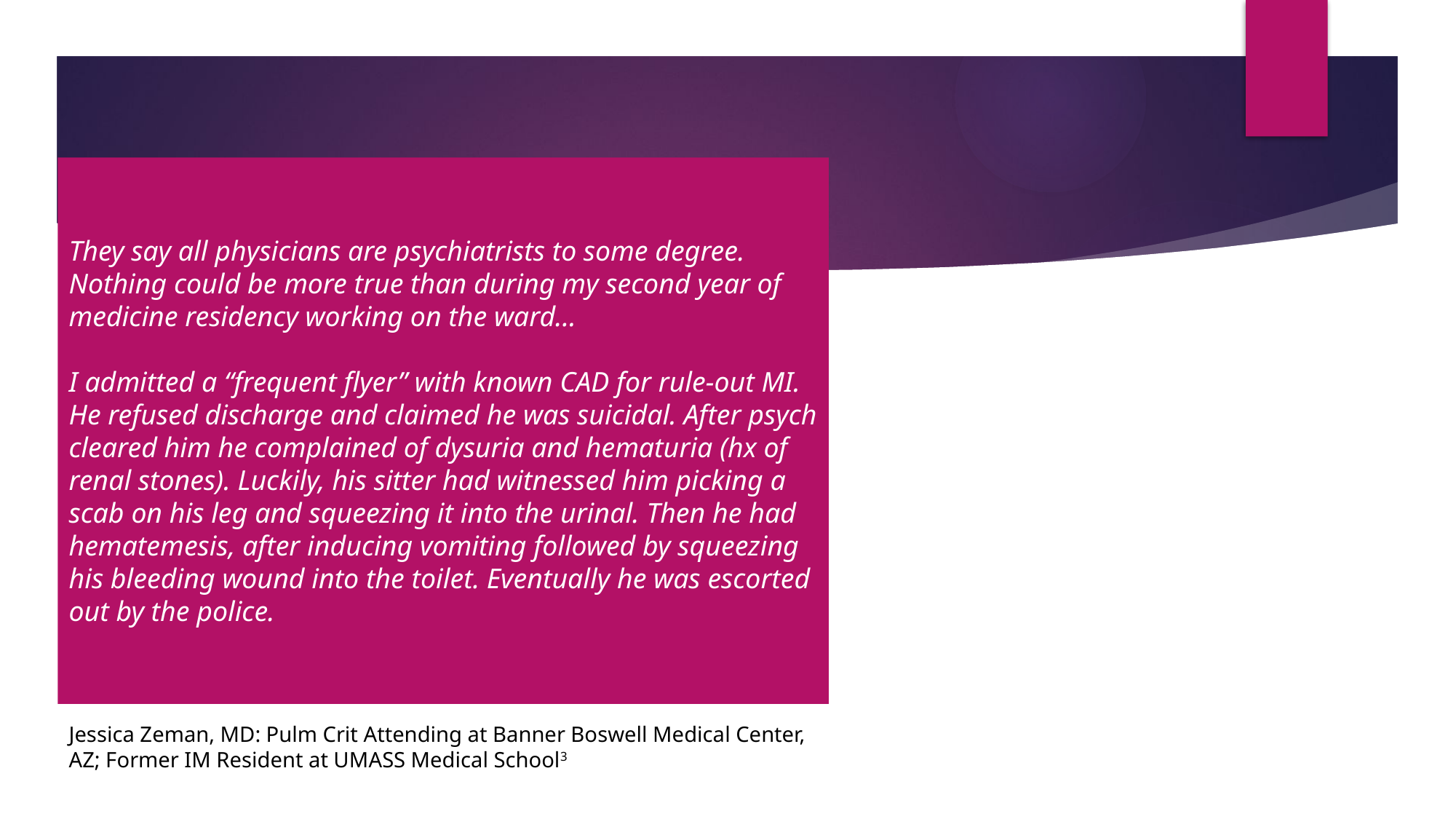

# They say all physicians are psychiatrists to some degree. Nothing could be more true than during my second year of medicine residency working on the ward... I admitted a “frequent flyer” with known CAD for rule-out MI. He refused discharge and claimed he was suicidal. After psych cleared him he complained of dysuria and hematuria (hx of renal stones). Luckily, his sitter had witnessed him picking a scab on his leg and squeezing it into the urinal. Then he had hematemesis, after inducing vomiting followed by squeezing his bleeding wound into the toilet. Eventually he was escorted out by the police.
Jessica Zeman, MD: Pulm Crit Attending at Banner Boswell Medical Center, AZ; Former IM Resident at UMASS Medical School3

## Slide 8
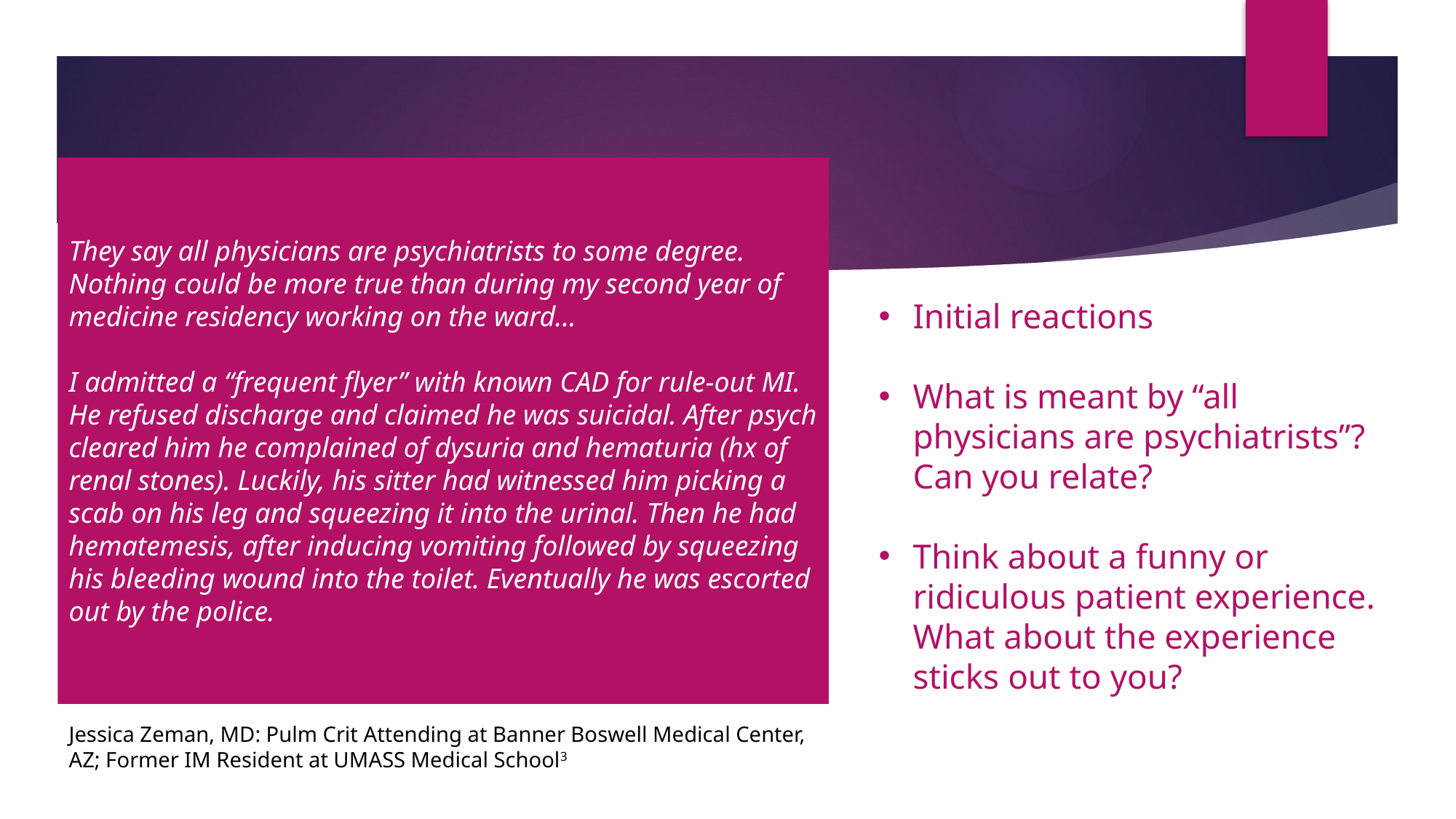

# They say all physicians are psychiatrists to some degree. Nothing could be more true than during my second year of medicine residency working on the ward... I admitted a “frequent flyer” with known CAD for rule-out MI. He refused discharge and claimed he was suicidal. After psych cleared him he complained of dysuria and hematuria (hx of renal stones). Luckily, his sitter had witnessed him picking a scab on his leg and squeezing it into the urinal. Then he had hematemesis, after inducing vomiting followed by squeezing his bleeding wound into the toilet. Eventually he was escorted out by the police.
Initial reactions
What is meant by “all physicians are psychiatrists”? Can you relate?
Think about a funny or ridiculous patient experience. What about the experience sticks out to you?
Jessica Zeman, MD: Pulm Crit Attending at Banner Boswell Medical Center, AZ; Former IM Resident at UMASS Medical School3

## Slide 9
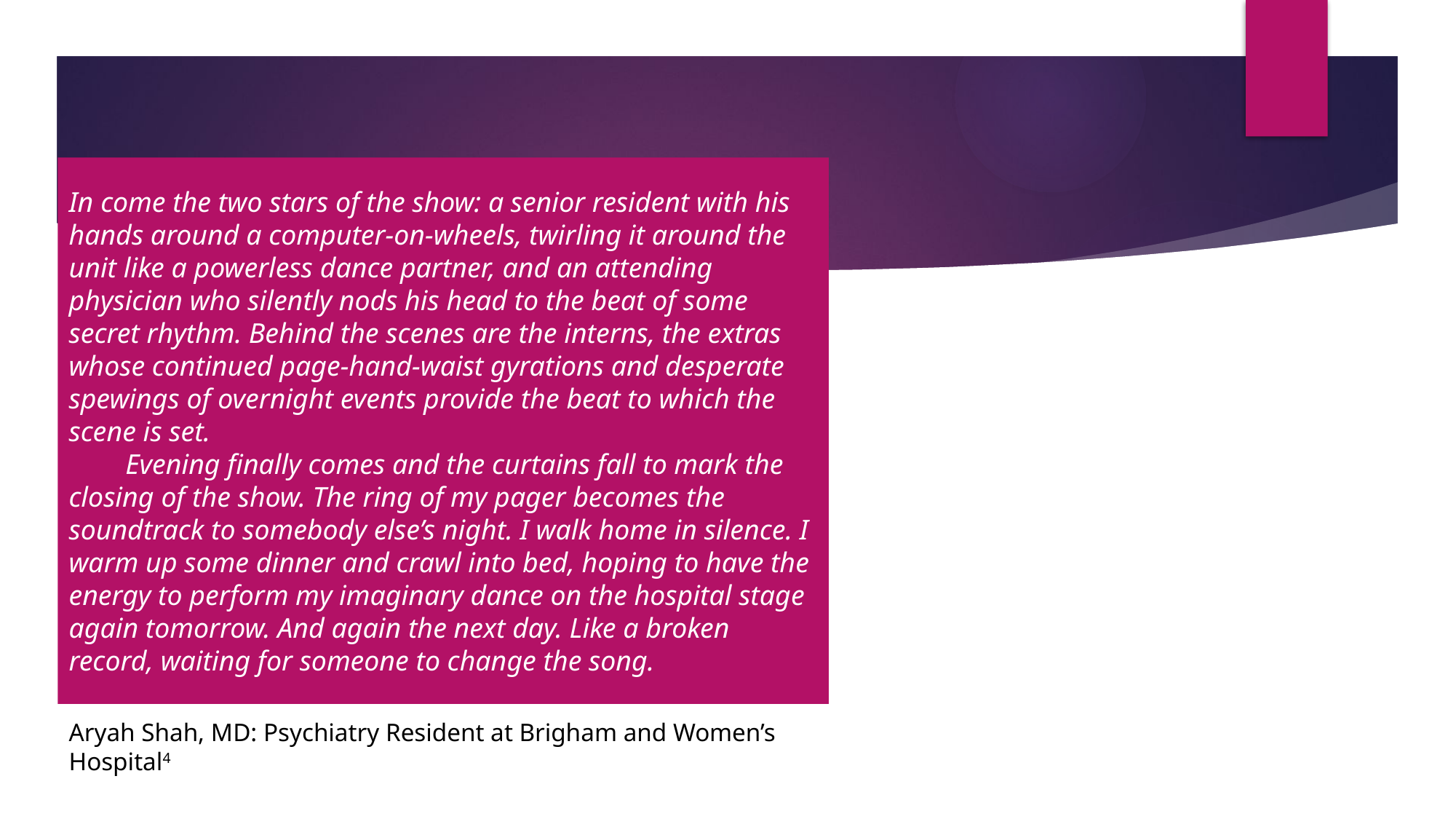

# In come the two stars of the show: a senior resident with his hands around a computer-on-wheels, twirling it around the unit like a powerless dance partner, and an attending physician who silently nods his head to the beat of some secret rhythm. Behind the scenes are the interns, the extras whose continued page-hand-waist gyrations and desperate spewings of overnight events provide the beat to which the scene is set. Evening finally comes and the curtains fall to mark the closing of the show. The ring of my pager becomes the soundtrack to somebody else’s night. I walk home in silence. I warm up some dinner and crawl into bed, hoping to have the energy to perform my imaginary dance on the hospital stage again tomorrow. And again the next day. Like a broken record, waiting for someone to change the song.
Aryah Shah, MD: Psychiatry Resident at Brigham and Women’s Hospital4

## Slide 10
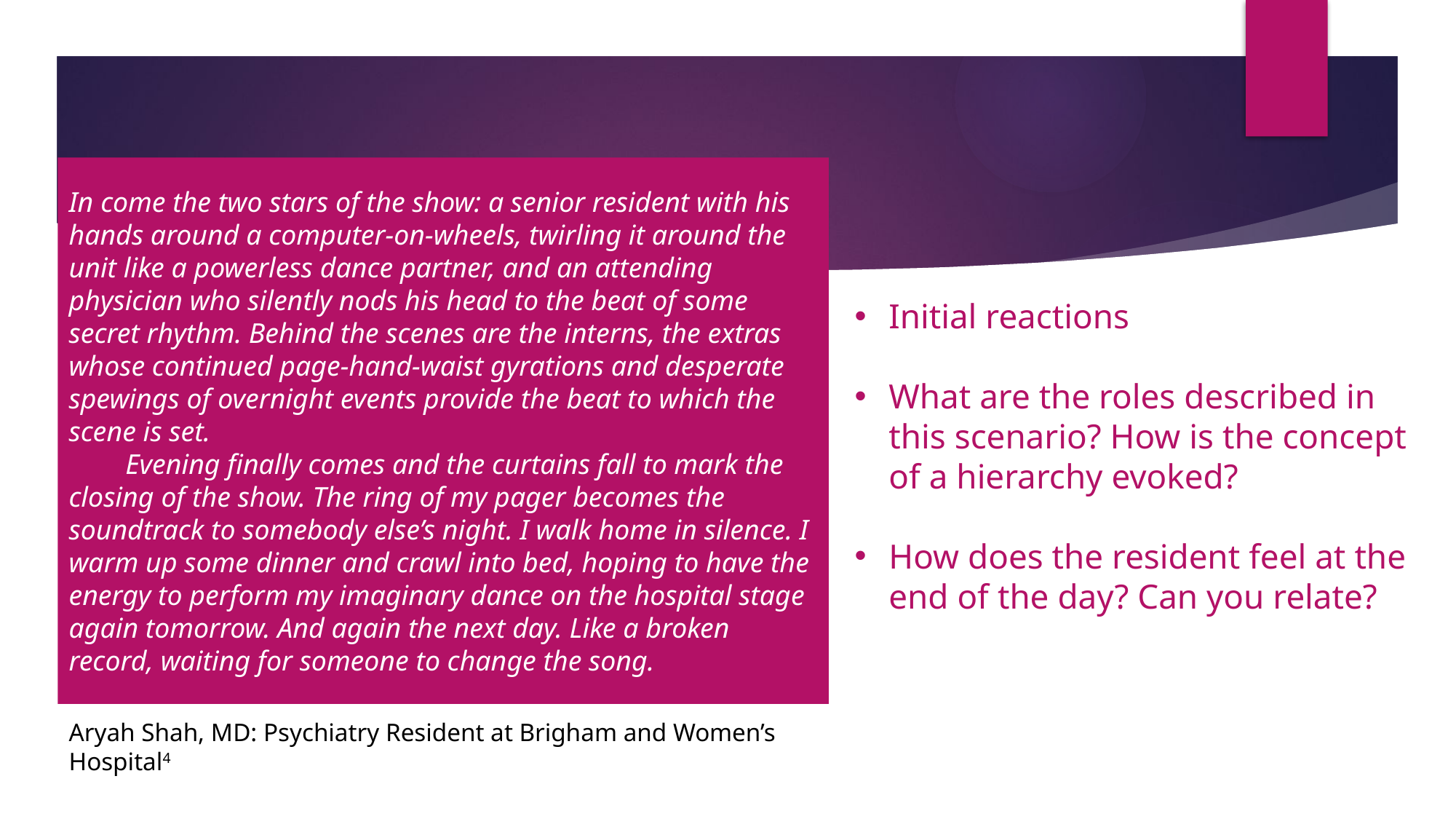

# In come the two stars of the show: a senior resident with his hands around a computer-on-wheels, twirling it around the unit like a powerless dance partner, and an attending physician who silently nods his head to the beat of some secret rhythm. Behind the scenes are the interns, the extras whose continued page-hand-waist gyrations and desperate spewings of overnight events provide the beat to which the scene is set. Evening finally comes and the curtains fall to mark the closing of the show. The ring of my pager becomes the soundtrack to somebody else’s night. I walk home in silence. I warm up some dinner and crawl into bed, hoping to have the energy to perform my imaginary dance on the hospital stage again tomorrow. And again the next day. Like a broken record, waiting for someone to change the song.
Initial reactions
What are the roles described in this scenario? How is the concept of a hierarchy evoked?
How does the resident feel at the end of the day? Can you relate?
Aryah Shah, MD: Psychiatry Resident at Brigham and Women’s Hospital4

## Slide 11
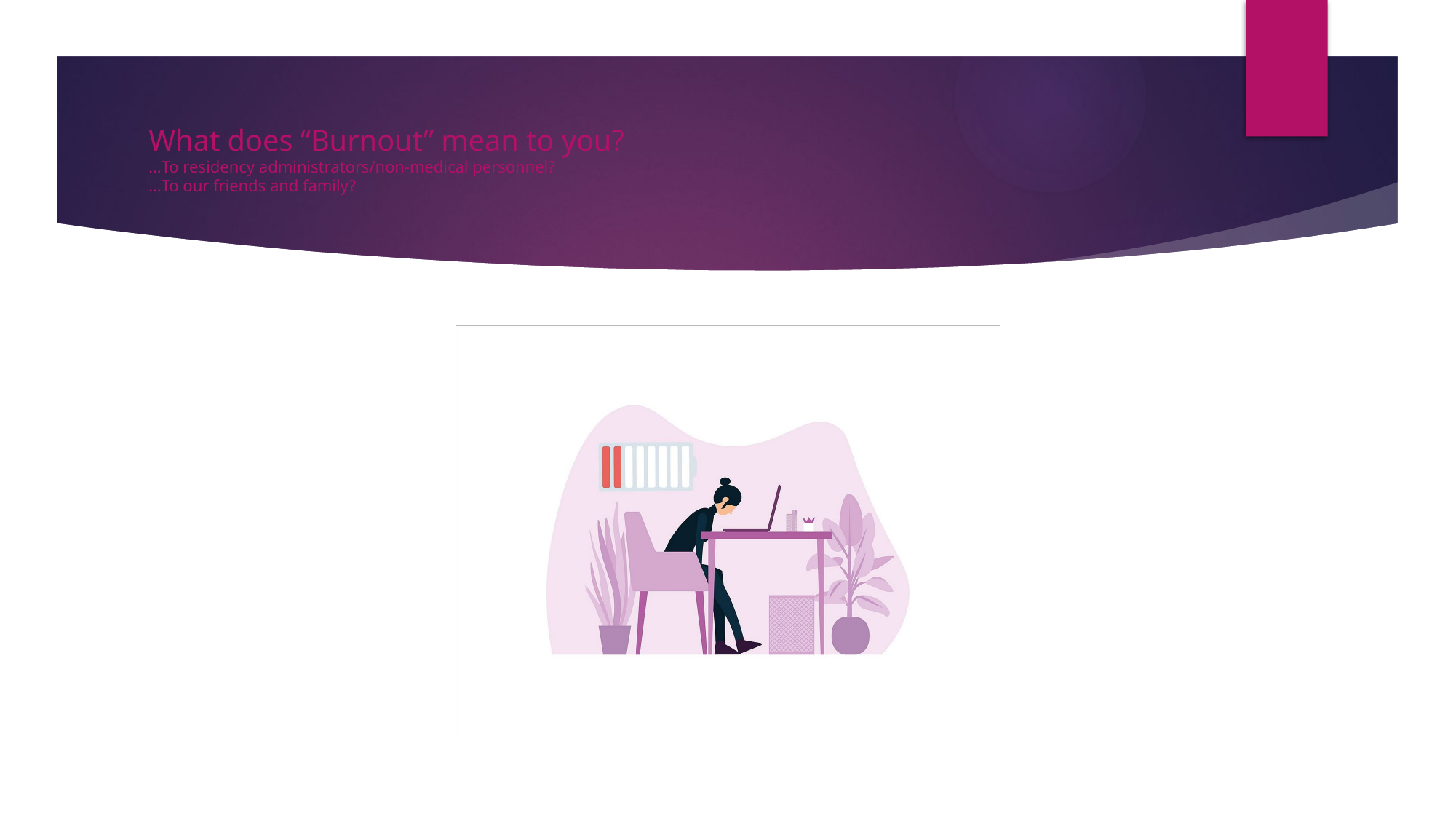

# What does “Burnout” mean to you? …To residency administrators/non-medical personnel? …To our friends and family?

## Slide 12
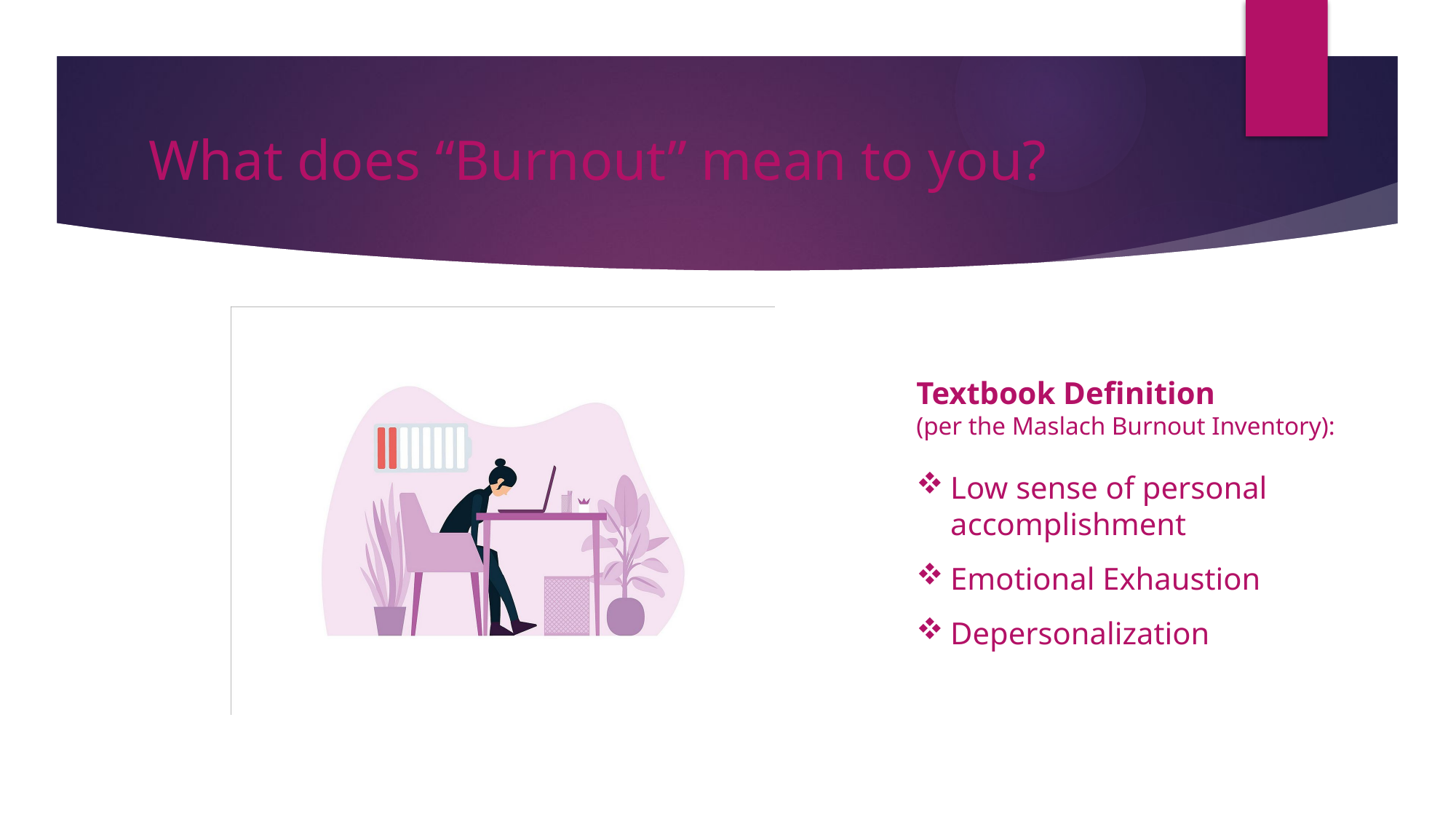

# What does “Burnout” mean to you?
Textbook Definition
(per the Maslach Burnout Inventory):
Low sense of personal accomplishment
Emotional Exhaustion
Depersonalization

## Slide 13
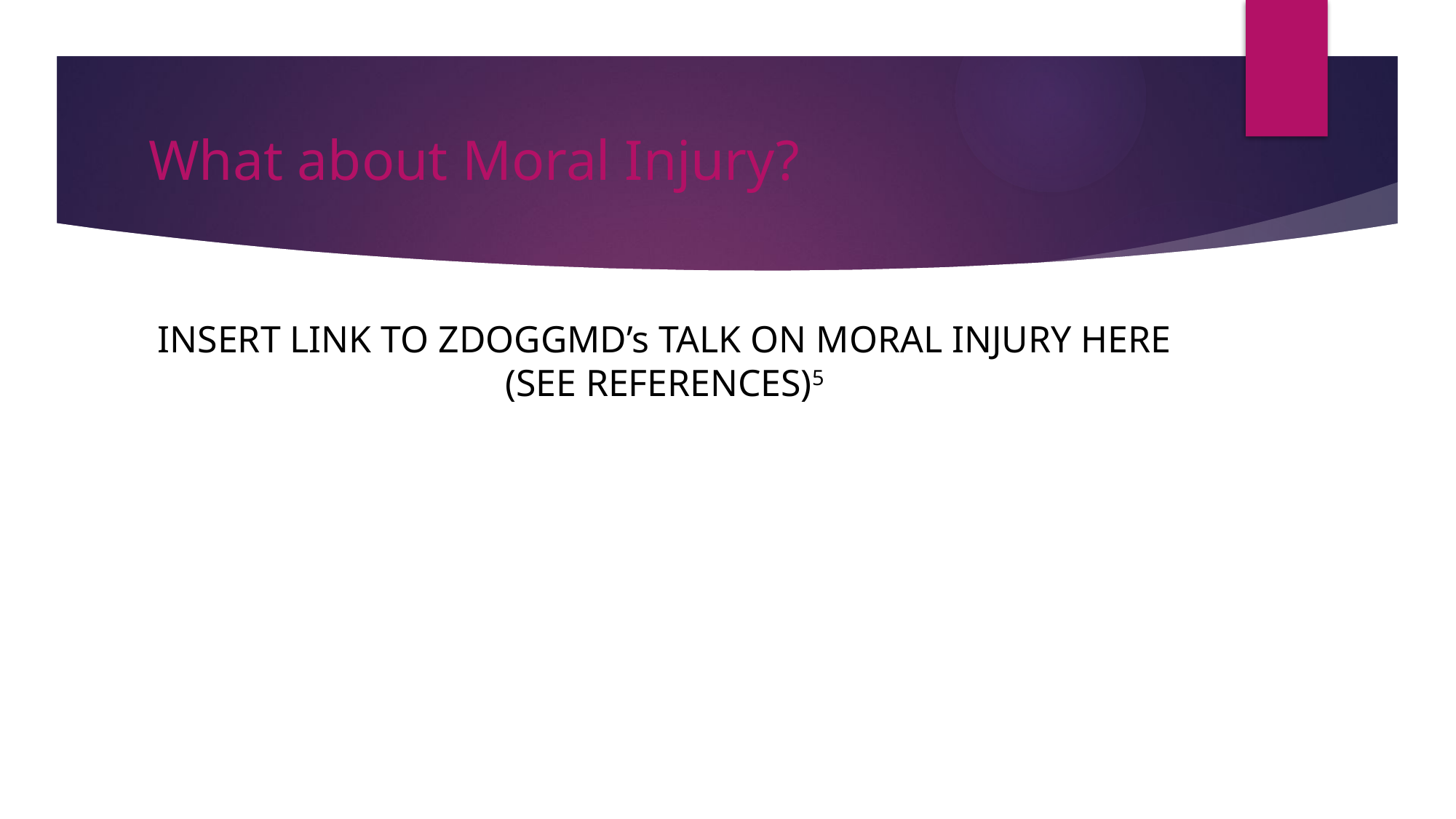

# What about Moral Injury?
INSERT LINK TO ZDOGGMD’s TALK ON MORAL INJURY HERE (SEE REFERENCES)5

## Slide 14
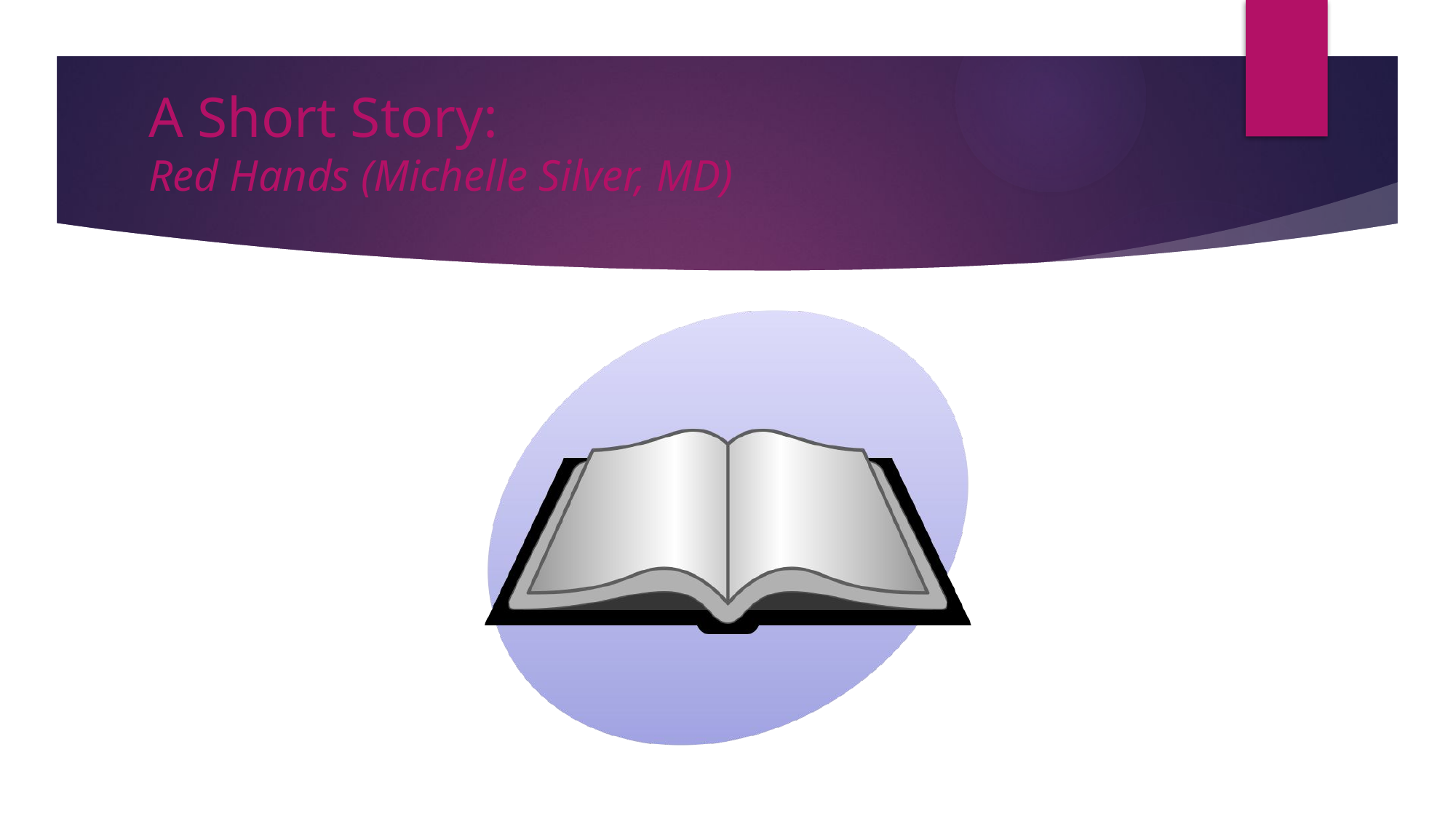

# A Short Story: Red Hands (Michelle Silver, MD)

## Slide 15
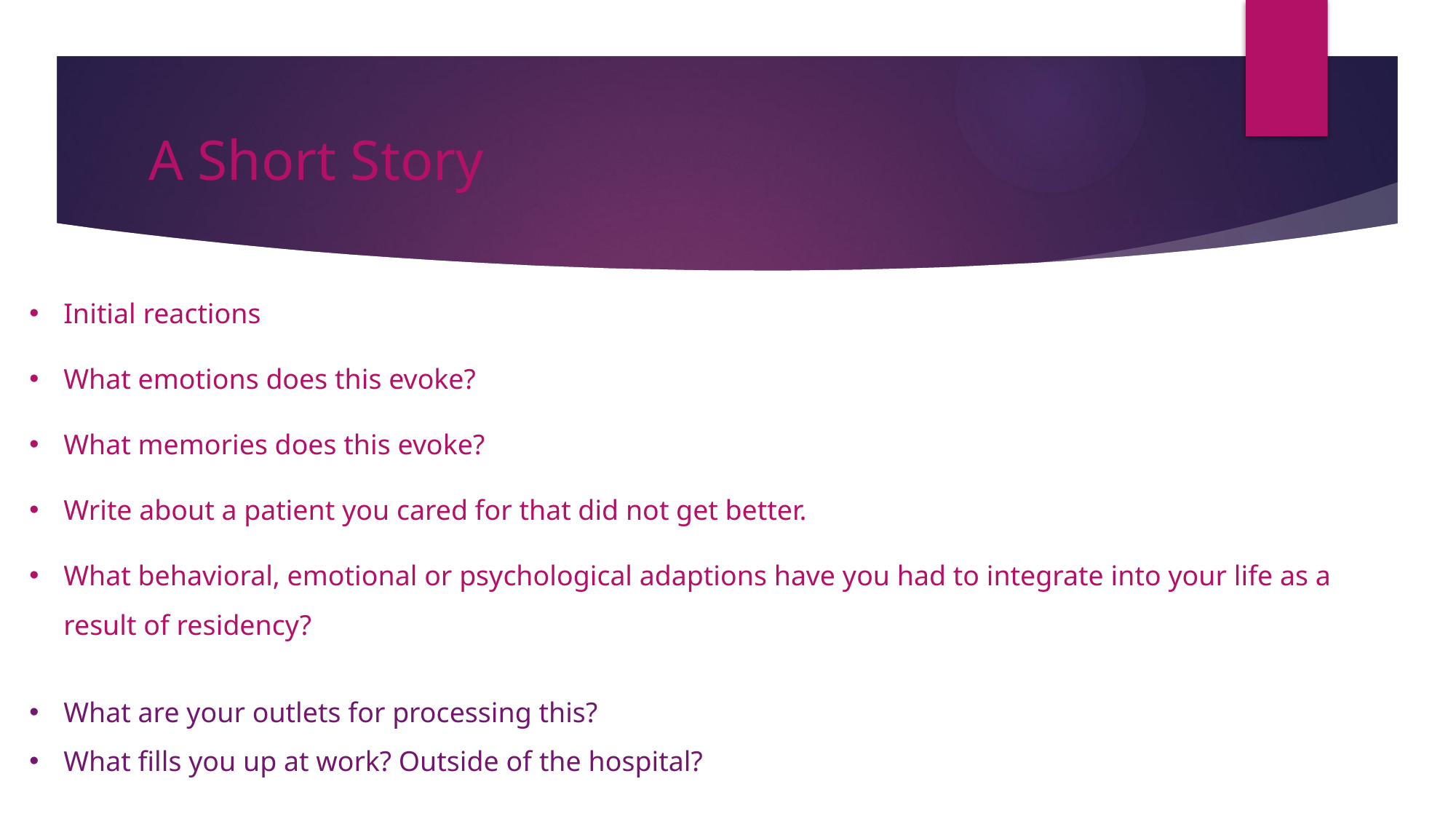

# A Short Story
Initial reactions
What emotions does this evoke?
What memories does this evoke?
Write about a patient you cared for that did not get better.
What behavioral, emotional or psychological adaptions have you had to integrate into your life as a result of residency?
What are your outlets for processing this?
What fills you up at work? Outside of the hospital?

## Slide 16
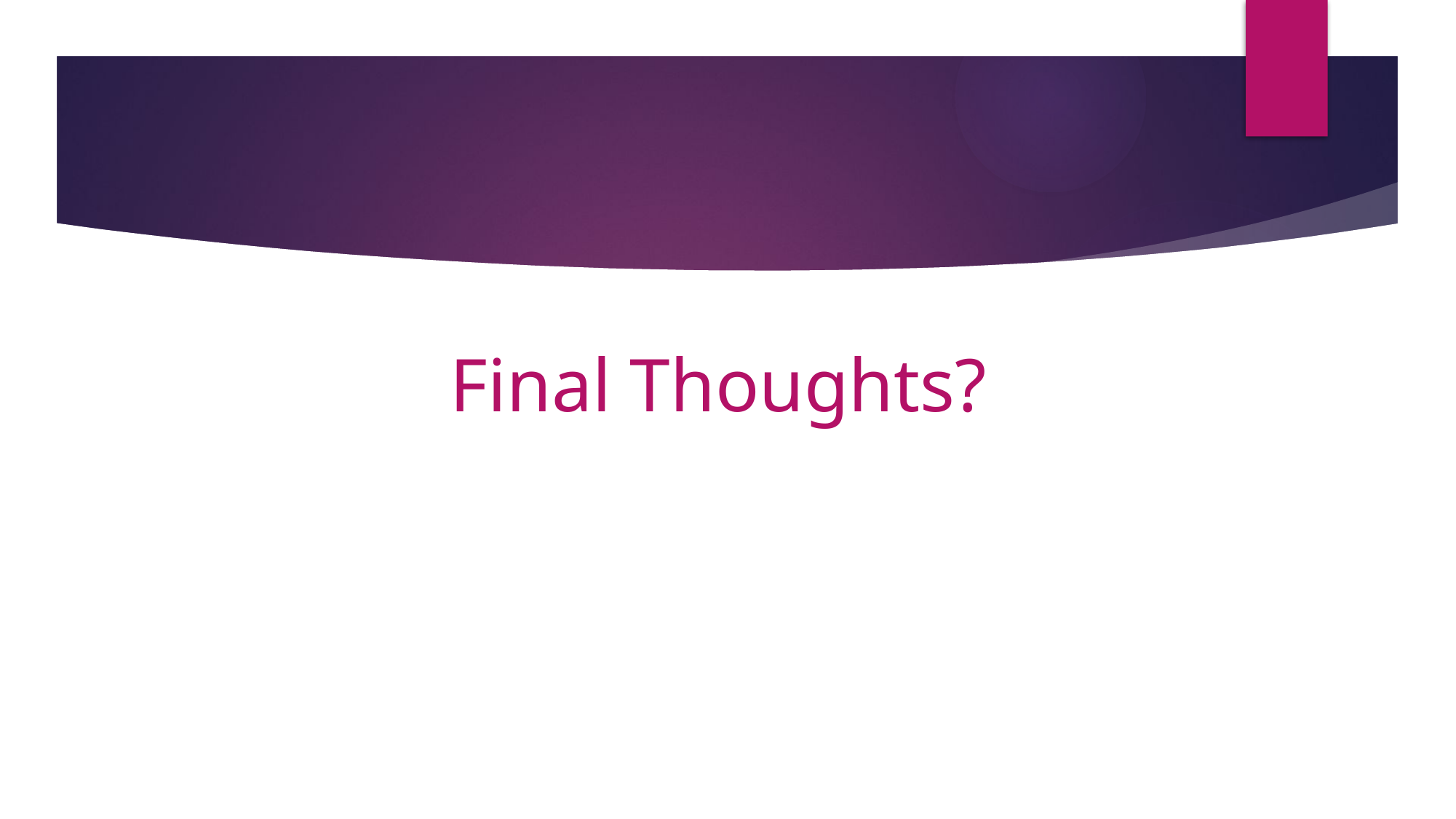

Final Thoughts?

## Slide 17
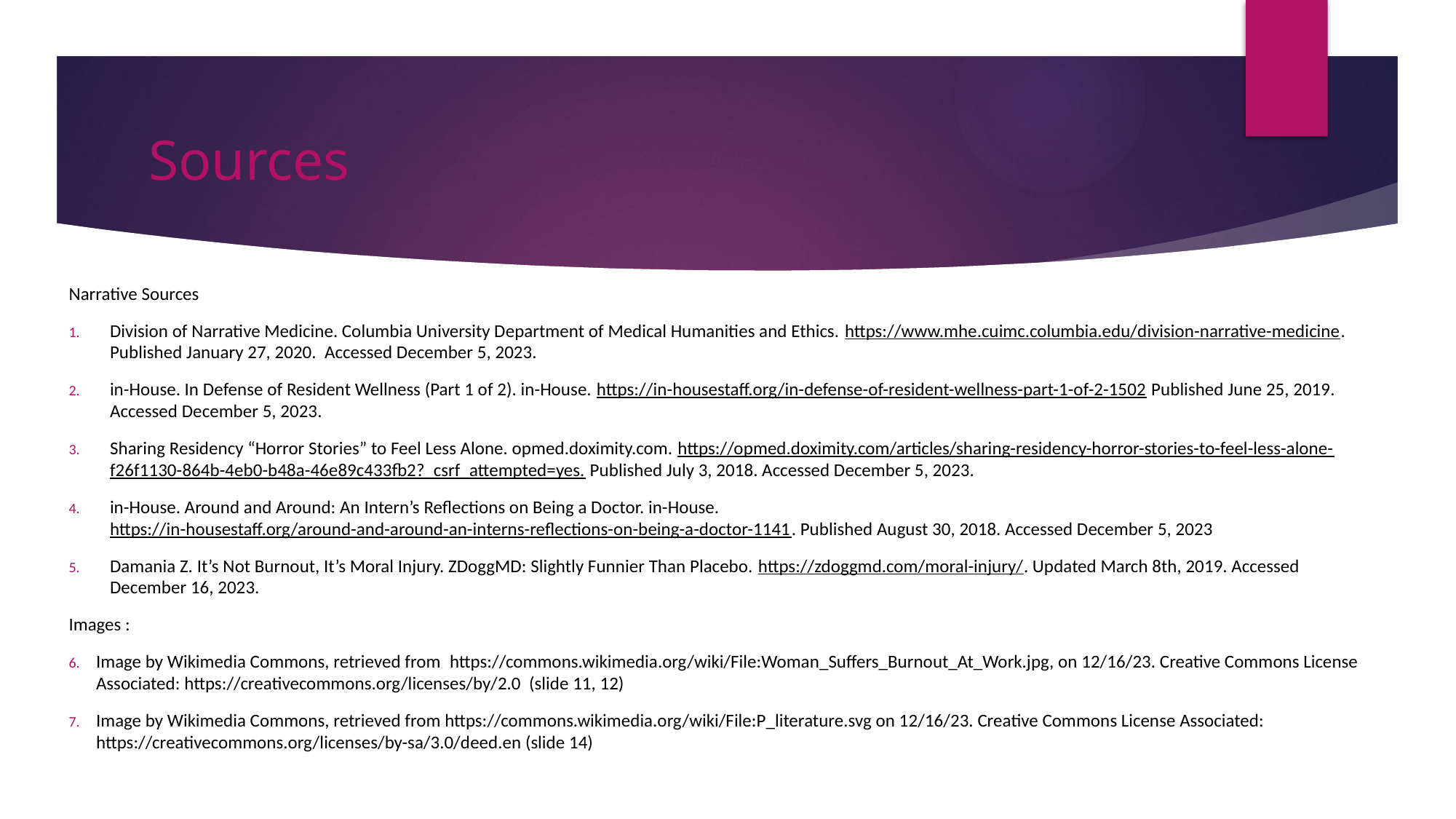

# Sources
Narrative Sources
Division of Narrative Medicine. Columbia University Department of Medical Humanities and Ethics. https://www.mhe.cuimc.columbia.edu/division-narrative-medicine. Published January 27, 2020. Accessed December 5, 2023.
in-House. In Defense of Resident Wellness (Part 1 of 2). in-House. https://in-housestaff.org/in-defense-of-resident-wellness-part-1-of-2-1502 Published June 25, 2019. Accessed December 5, 2023.
Sharing Residency “Horror Stories” to Feel Less Alone. opmed.doximity.com. https://opmed.doximity.com/articles/sharing-residency-horror-stories-to-feel-less-alone-f26f1130-864b-4eb0-b48a-46e89c433fb2?_csrf_attempted=yes. Published July 3, 2018. Accessed December 5, 2023.
in-House. Around and Around: An Intern’s Reflections on Being a Doctor. in-House. https://in-housestaff.org/around-and-around-an-interns-reflections-on-being-a-doctor-1141. Published August 30, 2018. Accessed December 5, 2023
Damania Z. It’s Not Burnout, It’s Moral Injury. ZDoggMD: Slightly Funnier Than Placebo. https://zdoggmd.com/moral-injury/. Updated March 8th, 2019. Accessed December 16, 2023.
Images :
Image by Wikimedia Commons, retrieved from  https://commons.wikimedia.org/wiki/File:Woman_Suffers_Burnout_At_Work.jpg, on 12/16/23. Creative Commons License Associated: https://creativecommons.org/licenses/by/2.0 (slide 11, 12)
Image by Wikimedia Commons, retrieved from https://commons.wikimedia.org/wiki/File:P_literature.svg on 12/16/23. Creative Commons License Associated: https://creativecommons.org/licenses/by-sa/3.0/deed.en (slide 14)

## Slide 18
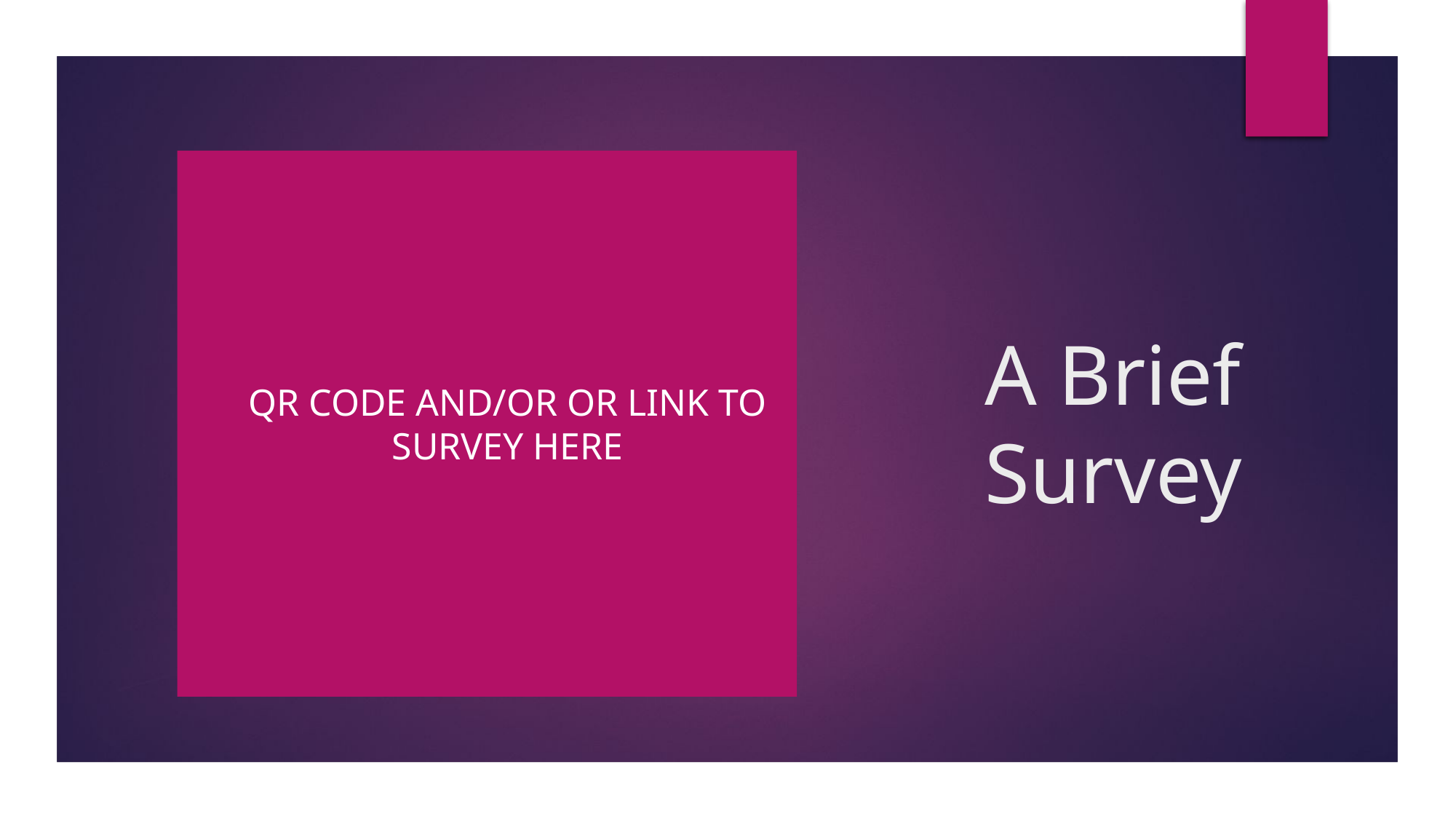

# A Brief Survey
QR CODE AND/OR OR LINK TO SURVEY HERE
