# Supplementary material for: A Resident Narrative Medicine Curriculum to Promote Professional Identity Development: Story-Based Sessions Grounded in Narrative Learning Theory
Source: MedEdPORTAL. 2024 Oct 22;20:11446. doi: 10.15766/mep_2374-8265.11446 (PMC11493853; doi:10.15766/mep_2374-8265.11446)
Supplement: Supplementary file 1 — Facilitator Guide.docxBurnout and Moral Injury.pptxCompassion Fatigue.pptxWorking Through a Pandemic.pptxDifficult Patient.pptxThe New Normal.pptxFinding Meaning.pptxUnpublished Narratives.docxSurvey.docx [file mep_2374-8265.11446-s001.zip › D. Working Through a Pandemic.pptx]

## Slide 1
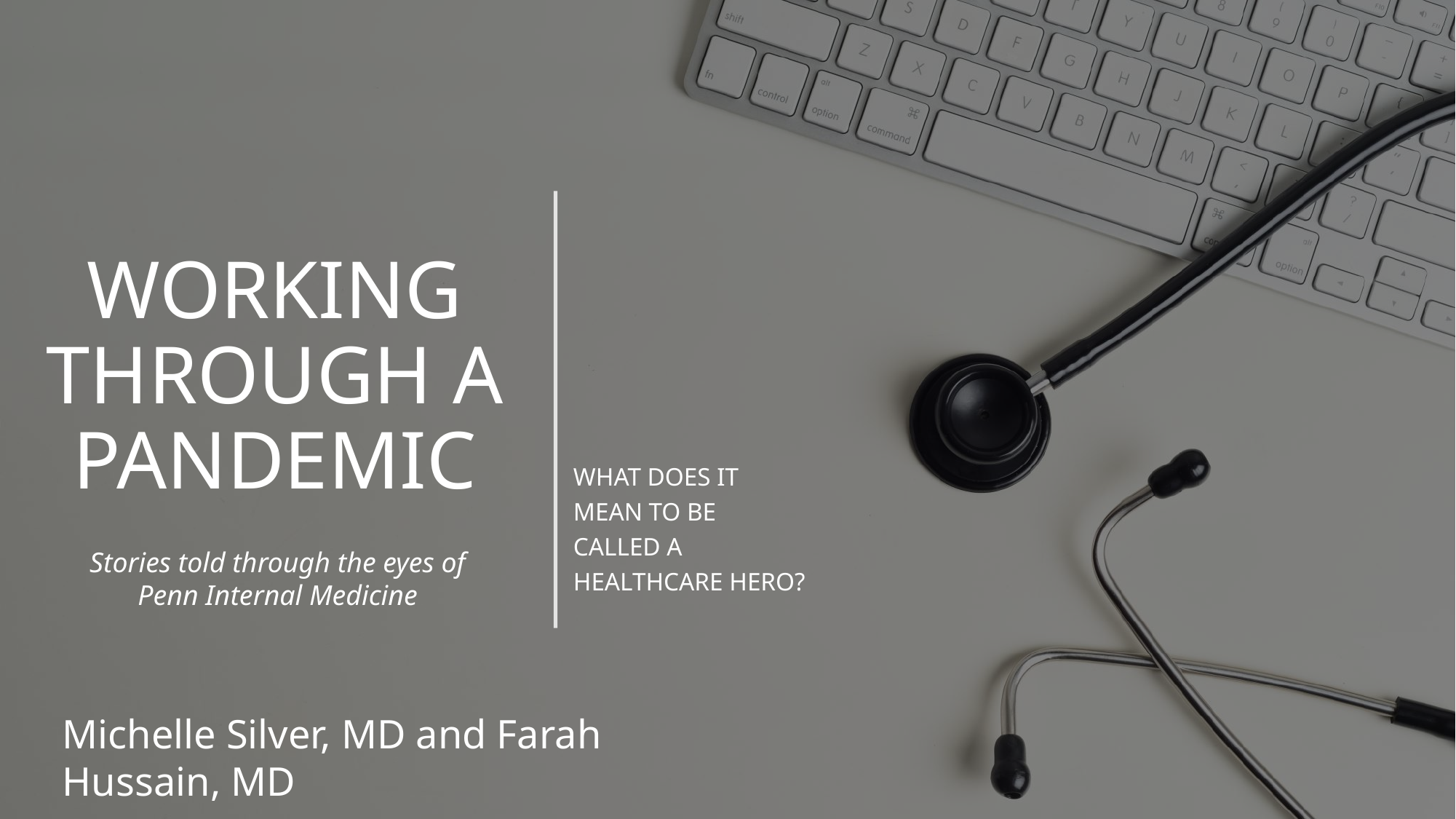

# Working through a pandemic
What does it mean to be called a healthcare hero?
Stories told through the eyes of Penn Internal Medicine
Michelle Silver, MD and Farah Hussain, MD

## Slide 2
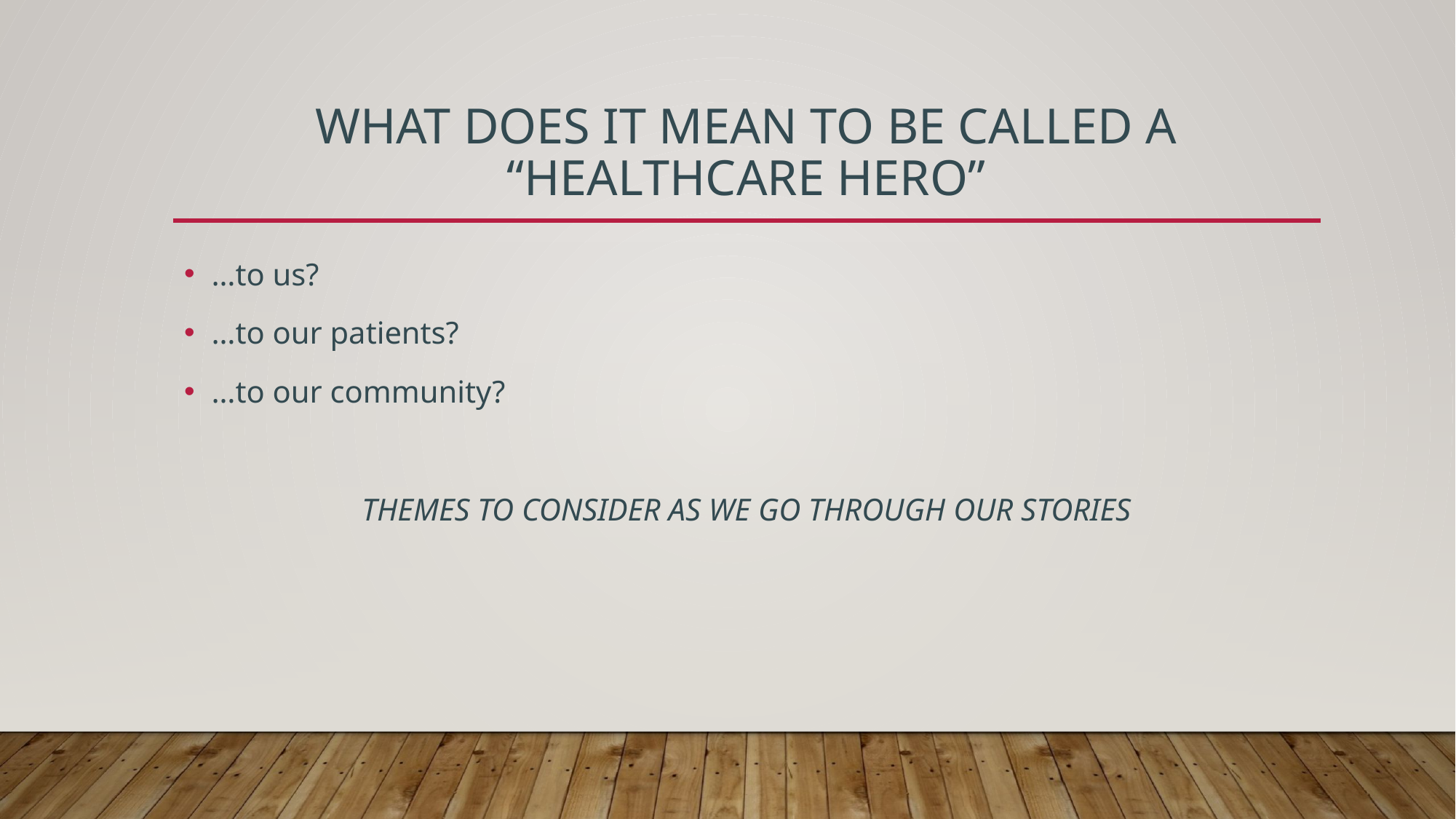

# WHAT DOES IT MEAN TO BE CALLED A “HEALTHCARE HERO”
…to us?
…to our patients?
…to our community?
THEMES TO CONSIDER AS WE GO THROUGH OUR STORIES

## Slide 3
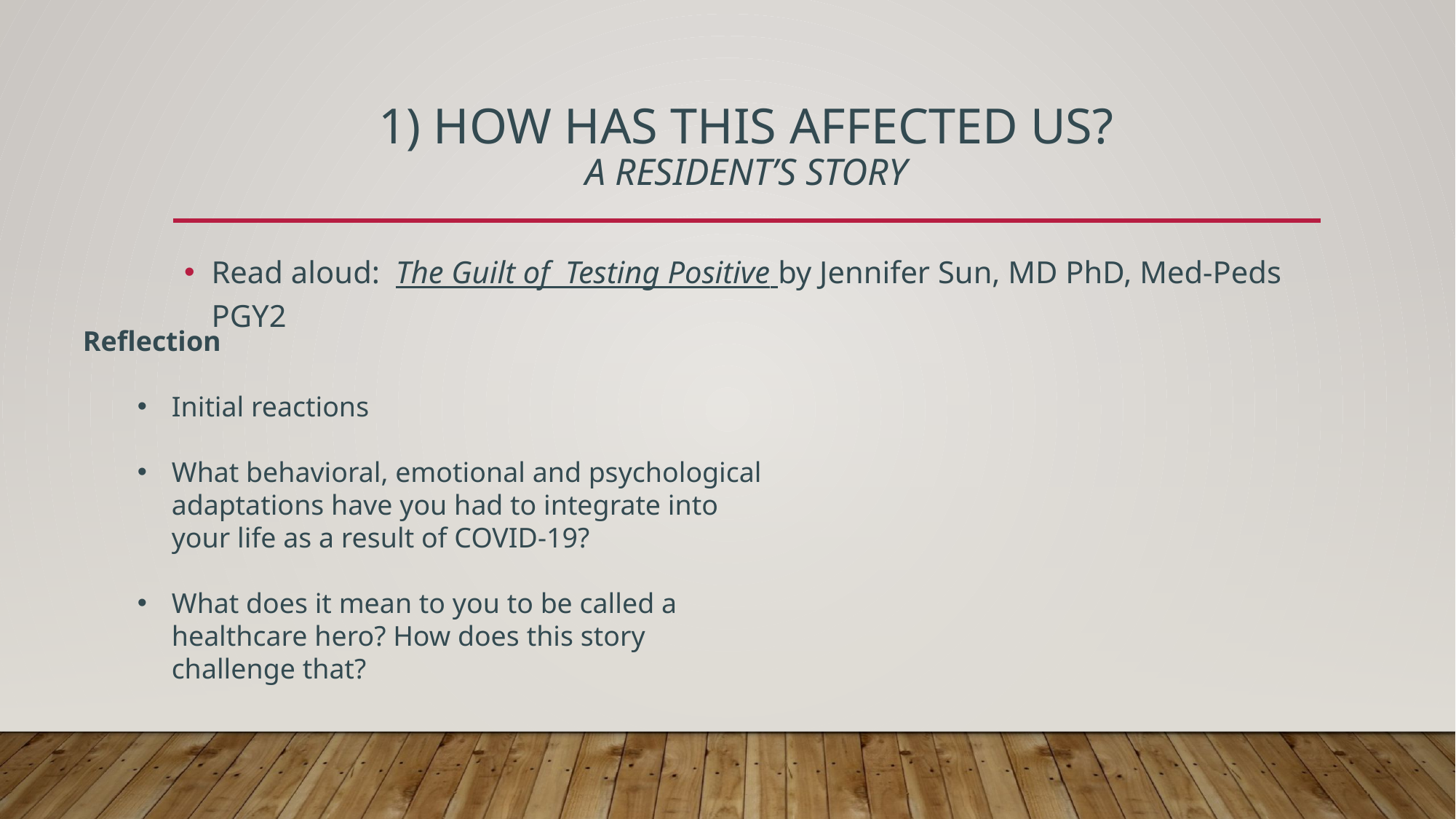

# 1) How has this affected us? A RESIDENT’S STORY
Read aloud: The Guilt of Testing Positive by Jennifer Sun, MD PhD, Med-Peds PGY2
Reflection
Initial reactions
What behavioral, emotional and psychological adaptations have you had to integrate into your life as a result of COVID-19?
What does it mean to you to be called a healthcare hero? How does this story challenge that?

## Slide 4
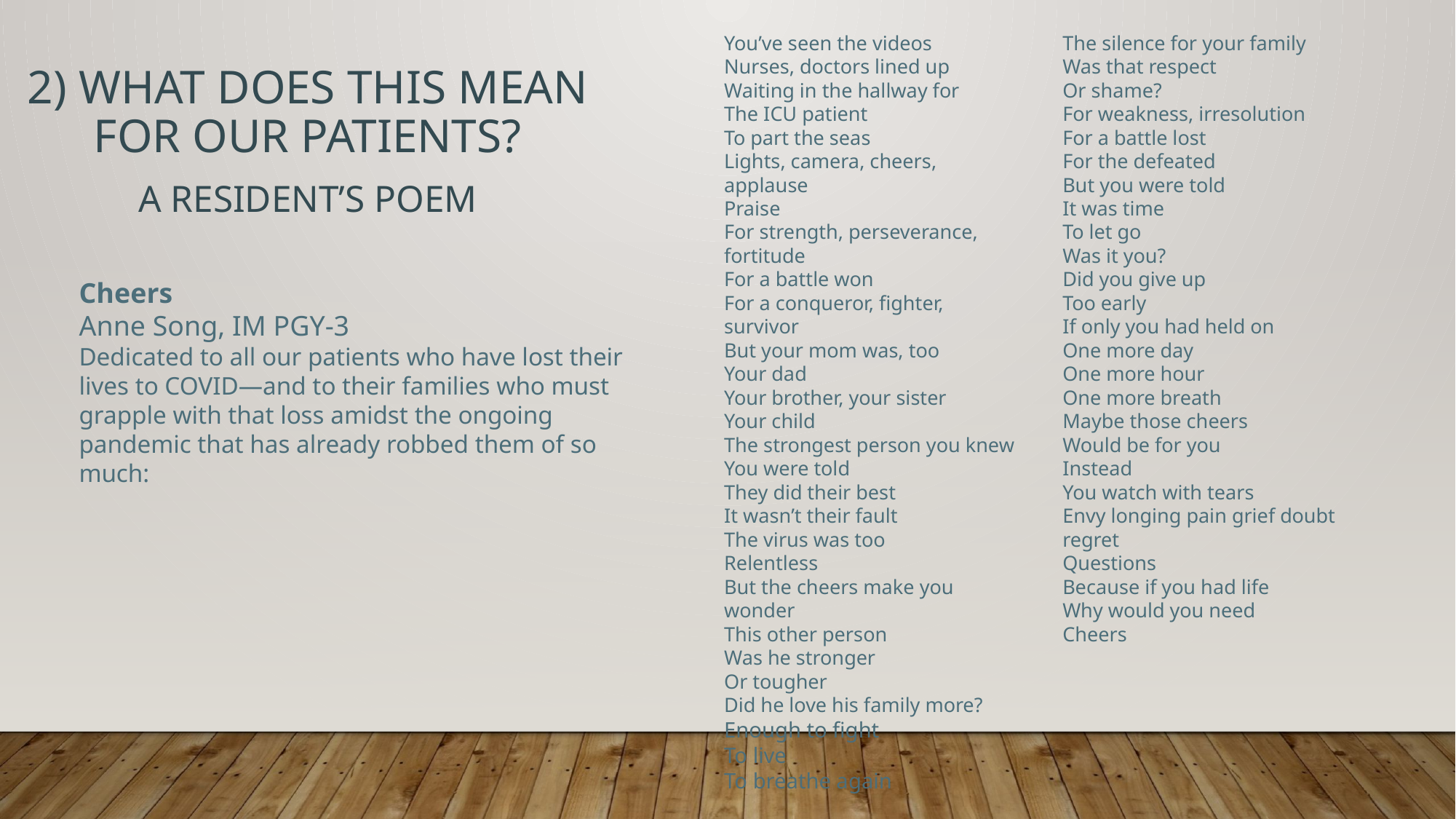

You’ve seen the videos
Nurses, doctors lined up
Waiting in the hallway for
The ICU patient
To part the seas
Lights, camera, cheers, applause
Praise
For strength, perseverance, fortitude
For a battle won
For a conqueror, fighter, survivor
But your mom was, too
Your dad
Your brother, your sister
Your child
The strongest person you knew
You were told
They did their best
It wasn’t their fault
The virus was too
Relentless
But the cheers make you wonder
This other person
Was he stronger
Or tougher
Did he love his family more?
Enough to fight
To live
To breathe again
The silence for your family
Was that respect
Or shame?
For weakness, irresolution
For a battle lost
For the defeated
But you were told
It was time
To let go
Was it you?
Did you give up
Too early
If only you had held on
One more day
One more hour
One more breath
Maybe those cheers
Would be for you
Instead
You watch with tears
Envy longing pain grief doubt regret
Questions
Because if you had life
Why would you need
Cheers
2) What does this mean for our patients?
A resident’s poem
Cheers
Anne Song, IM PGY-3
Dedicated to all our patients who have lost their lives to COVID—and to their families who must grapple with that loss amidst the ongoing pandemic that has already robbed them of so much:

## Slide 5
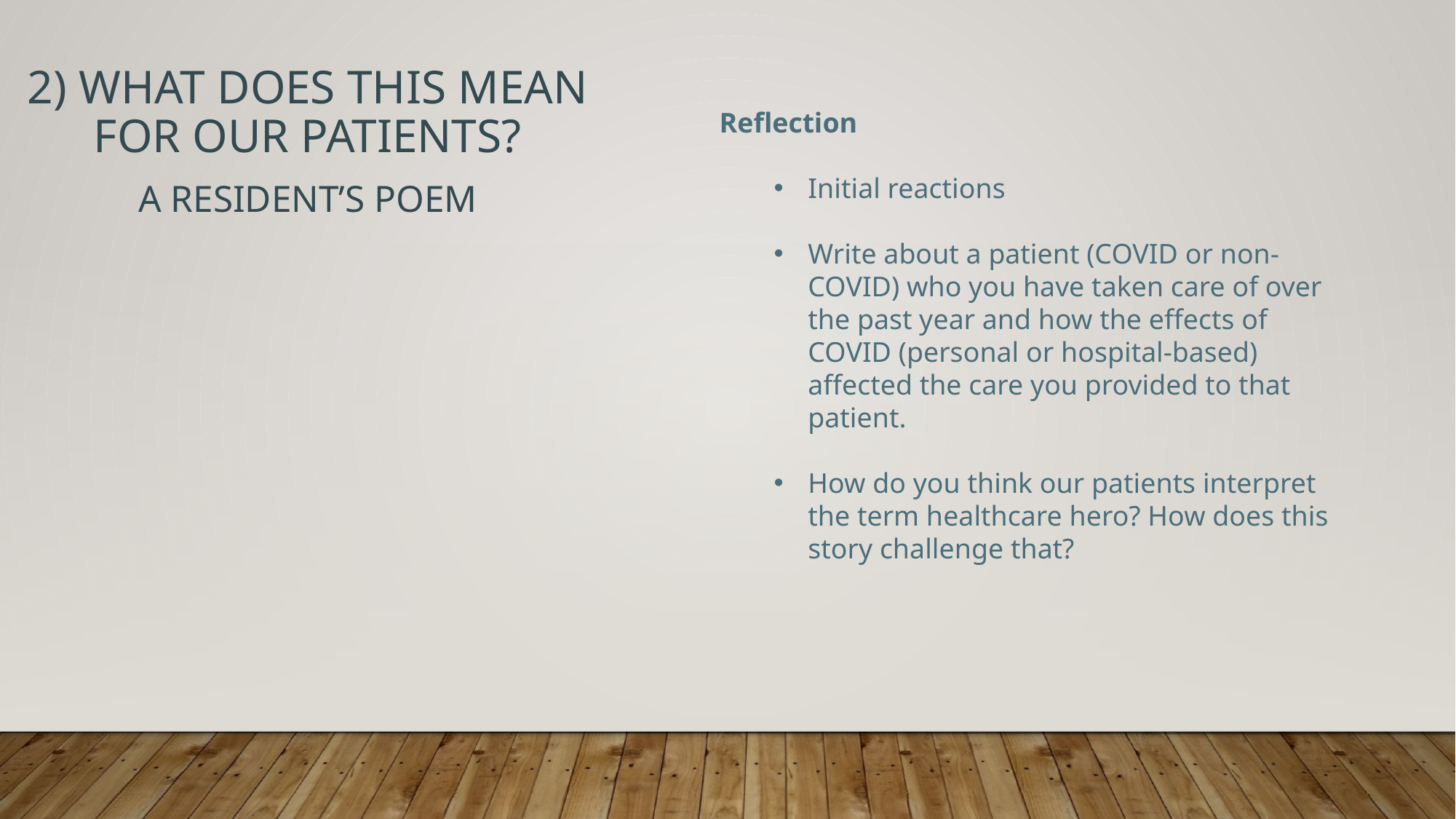

2) What does this mean for our patients?
A resident’s poem
Reflection
Initial reactions
Write about a patient (COVID or non-COVID) who you have taken care of over the past year and how the effects of COVID (personal or hospital-based) affected the care you provided to that patient.
How do you think our patients interpret the term healthcare hero? How does this story challenge that?

## Slide 6
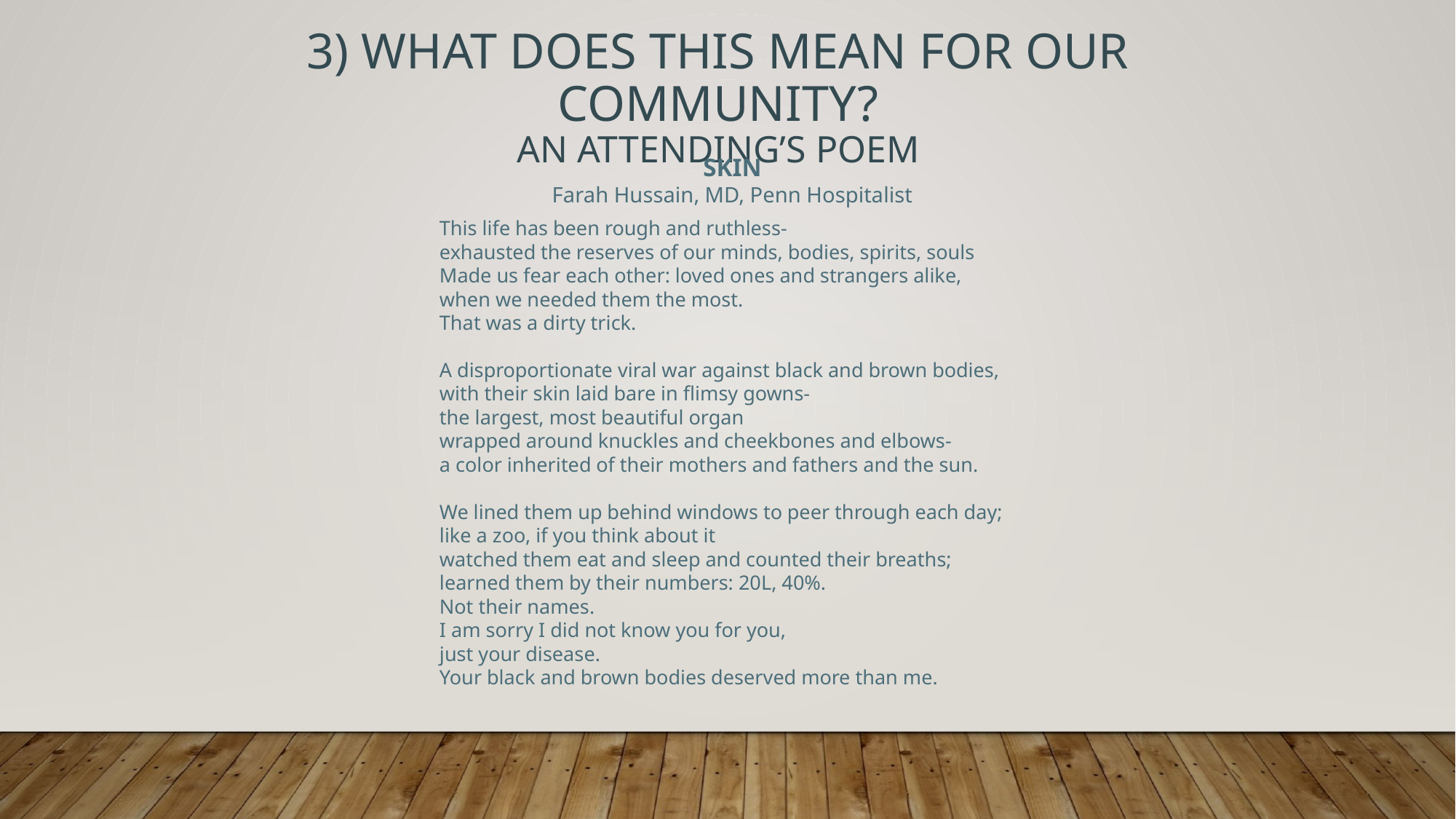

3) What does this mean for our community?
AN ATTENDING’S POEM
SKIN
Farah Hussain, MD, Penn Hospitalist
This life has been rough and ruthless-
exhausted the reserves of our minds, bodies, spirits, souls
Made us fear each other: loved ones and strangers alike,
when we needed them the most.
That was a dirty trick.
A disproportionate viral war against black and brown bodies,
with their skin laid bare in flimsy gowns-
the largest, most beautiful organ
wrapped around knuckles and cheekbones and elbows-
a color inherited of their mothers and fathers and the sun.
We lined them up behind windows to peer through each day;
like a zoo, if you think about it
watched them eat and sleep and counted their breaths;
learned them by their numbers: 20L, 40%.
Not their names.
I am sorry I did not know you for you,
just your disease.
Your black and brown bodies deserved more than me.

## Slide 7
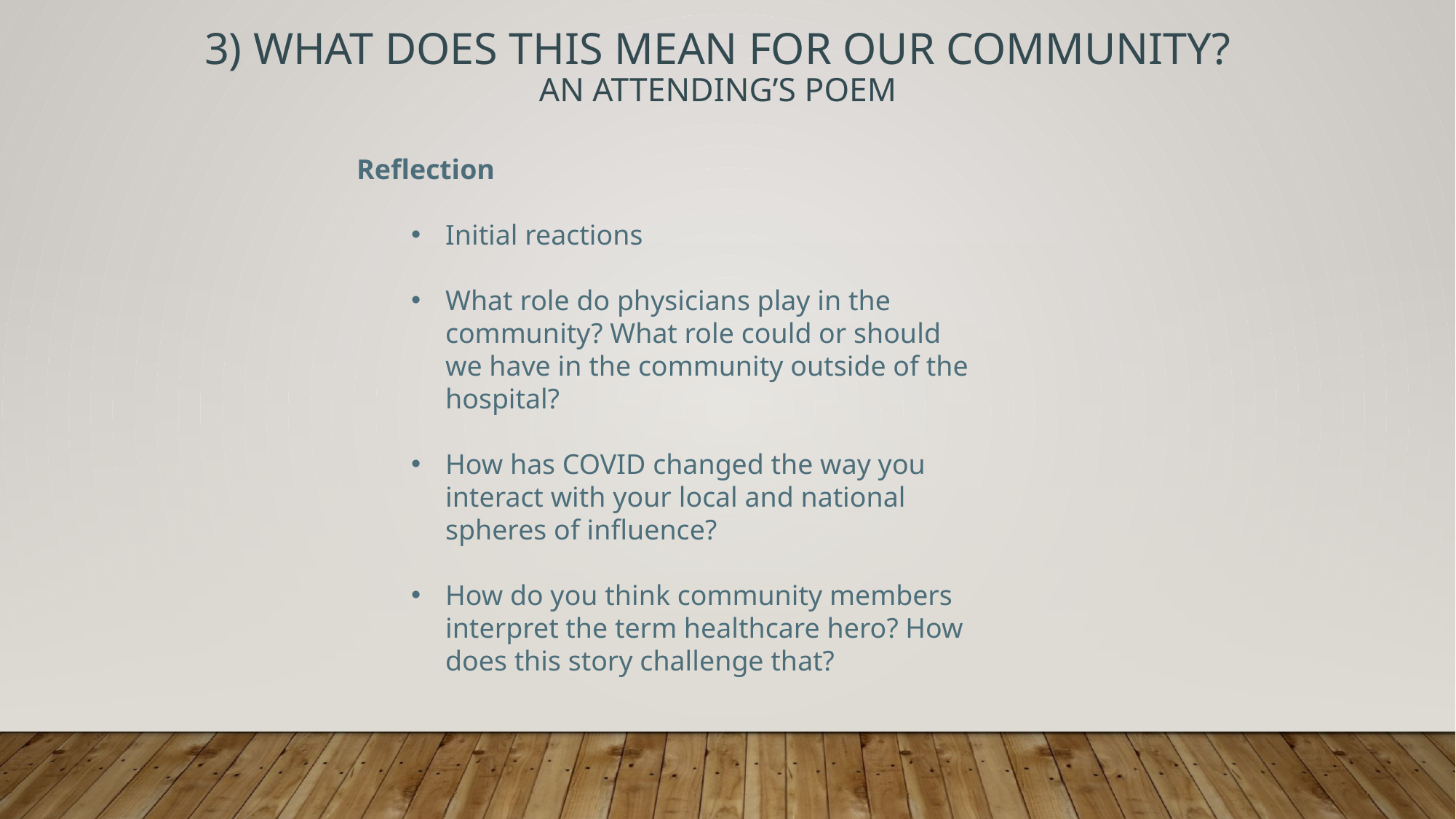

3) What does this mean for our community?
AN ATTENDING’s poem
Reflection
Initial reactions
What role do physicians play in the community? What role could or should we have in the community outside of the hospital?
How has COVID changed the way you interact with your local and national spheres of influence?
How do you think community members interpret the term healthcare hero? How does this story challenge that?

## Slide 8
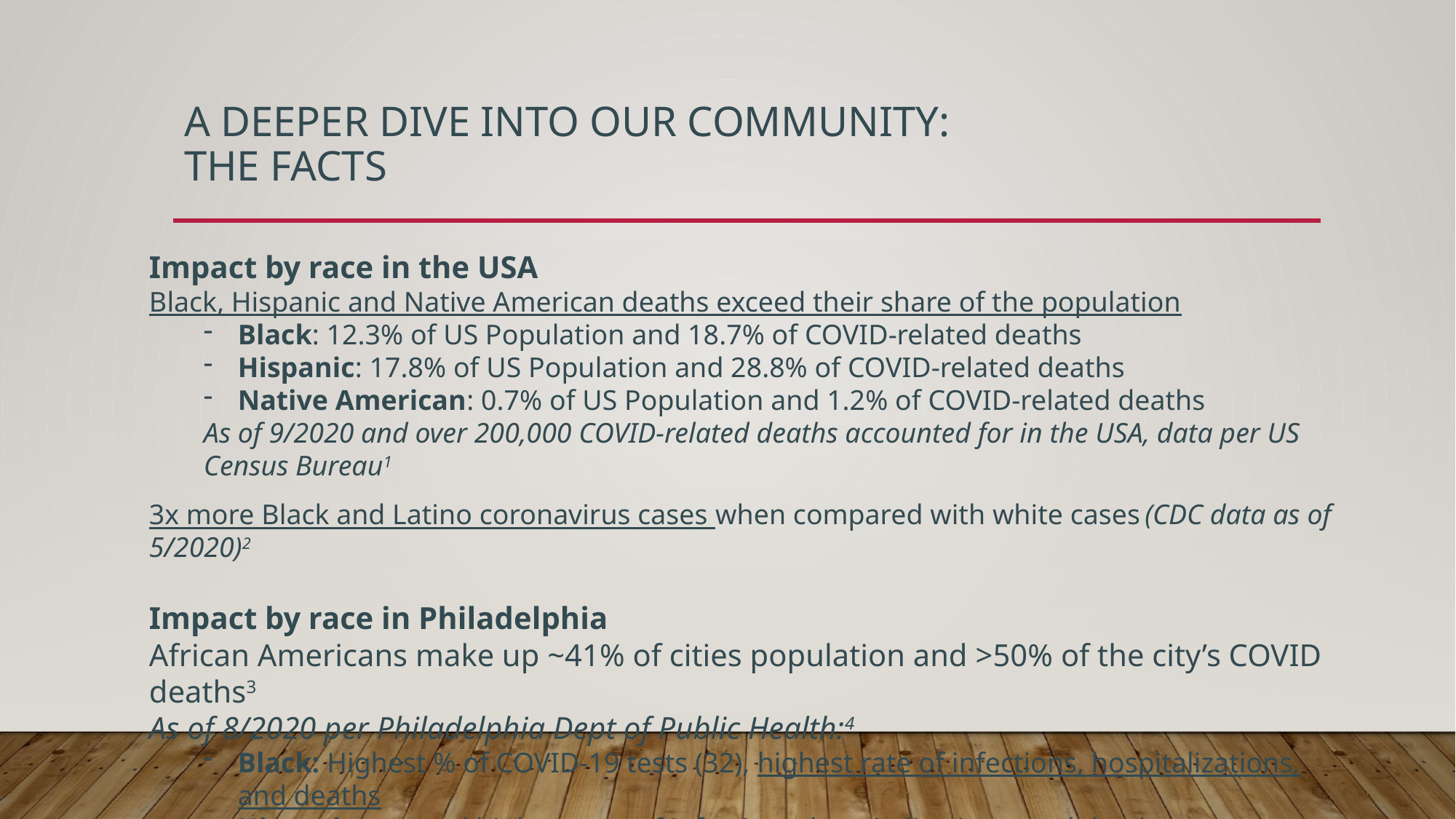

# A deeper dive into our community: the facts
Impact by race in the USA
Black, Hispanic and Native American deaths exceed their share of the population
Black: 12.3% of US Population and 18.7% of COVID-related deaths
Hispanic: 17.8% of US Population and 28.8% of COVID-related deaths
Native American: 0.7% of US Population and 1.2% of COVID-related deaths
As of 9/2020 and over 200,000 COVID-related deaths accounted for in the USA, data per US Census Bureau1
3x more Black and Latino coronavirus cases when compared with white cases (CDC data as of 5/2020)2
Impact by race in Philadelphia
African Americans make up ~41% of cities population and >50% of the city’s COVID deaths3
As of 8/2020 per Philadelphia Dept of Public Health:4
Black: Highest % of COVID-19 tests (32), highest rate of infections, hospitalizations, and deaths
Hispanic: second highest rate of infections, hospitalizations, and deaths

## Slide 9
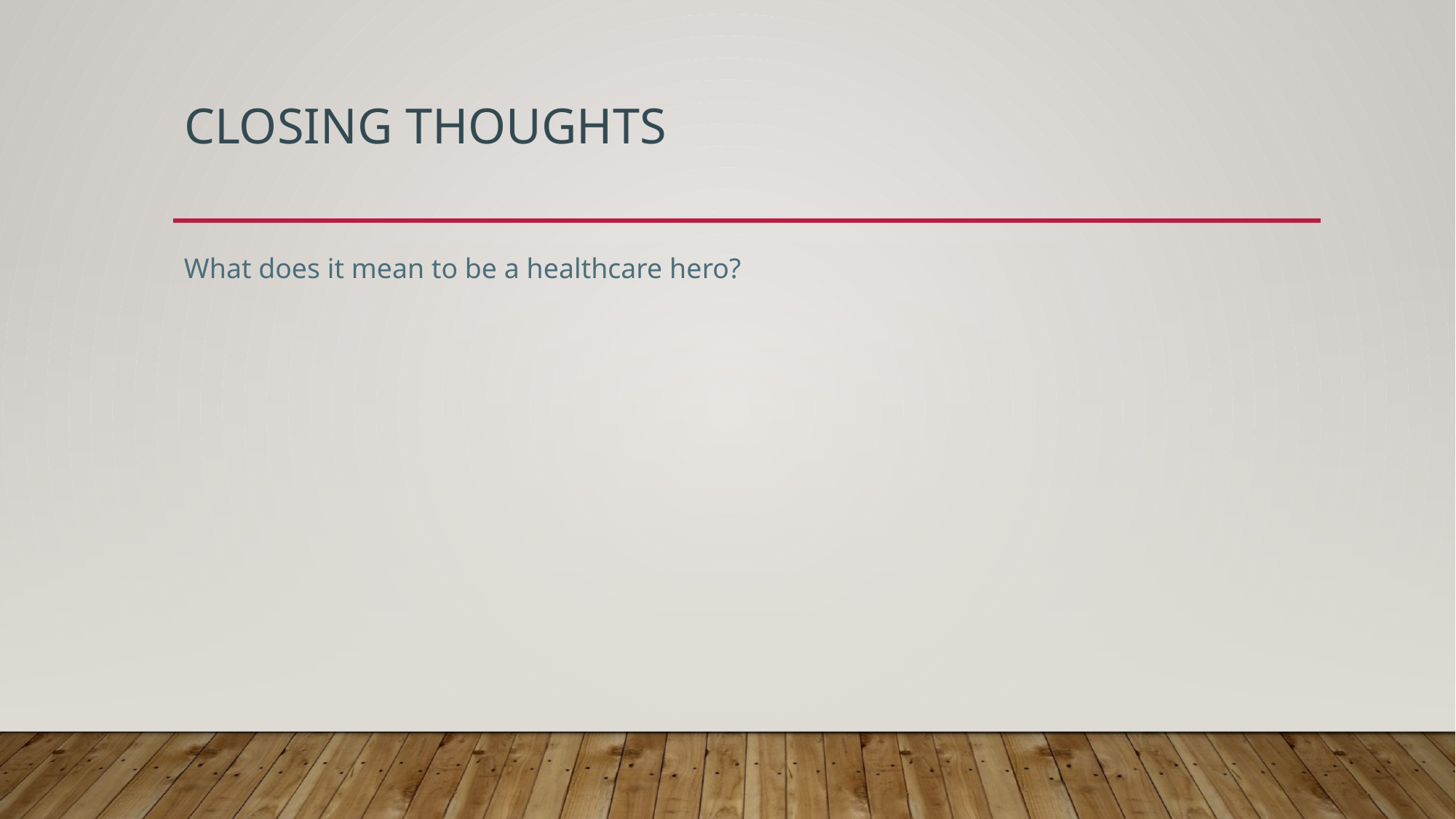

# Closing thoughts
What does it mean to be a healthcare hero?

## Slide 10
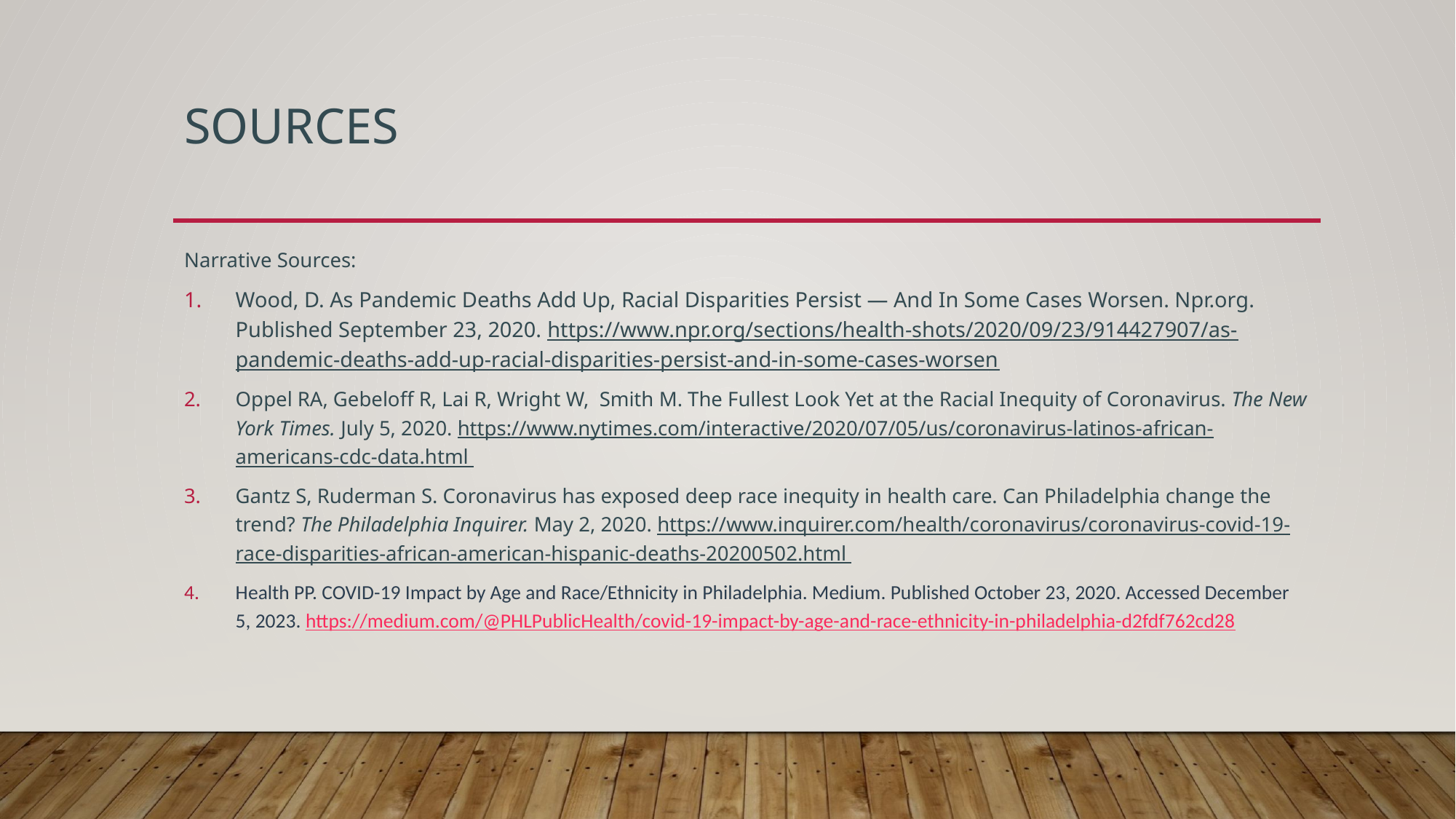

# SOURCES
Narrative Sources:
Wood, D. As Pandemic Deaths Add Up, Racial Disparities Persist — And In Some Cases Worsen. Npr.org. Published September 23, 2020. https://www.npr.org/sections/health-shots/2020/09/23/914427907/as-pandemic-deaths-add-up-racial-disparities-persist-and-in-some-cases-worsen
Oppel RA, Gebeloff R, Lai R, Wright W, Smith M. The Fullest Look Yet at the Racial Inequity of Coronavirus. The New York Times. July 5, 2020. https://www.nytimes.com/interactive/2020/07/05/us/coronavirus-latinos-african-americans-cdc-data.html
Gantz S, Ruderman S. Coronavirus has exposed deep race inequity in health care. Can Philadelphia change the trend? The Philadelphia Inquirer. May 2, 2020. https://www.inquirer.com/health/coronavirus/coronavirus-covid-19-race-disparities-african-american-hispanic-deaths-20200502.html
Health PP. COVID-19 Impact by Age and Race/Ethnicity in Philadelphia. Medium. Published October 23, 2020. Accessed December 5, 2023. https://medium.com/@PHLPublicHealth/covid-19-impact-by-age-and-race-ethnicity-in-philadelphia-d2fdf762cd28

## Slide 11
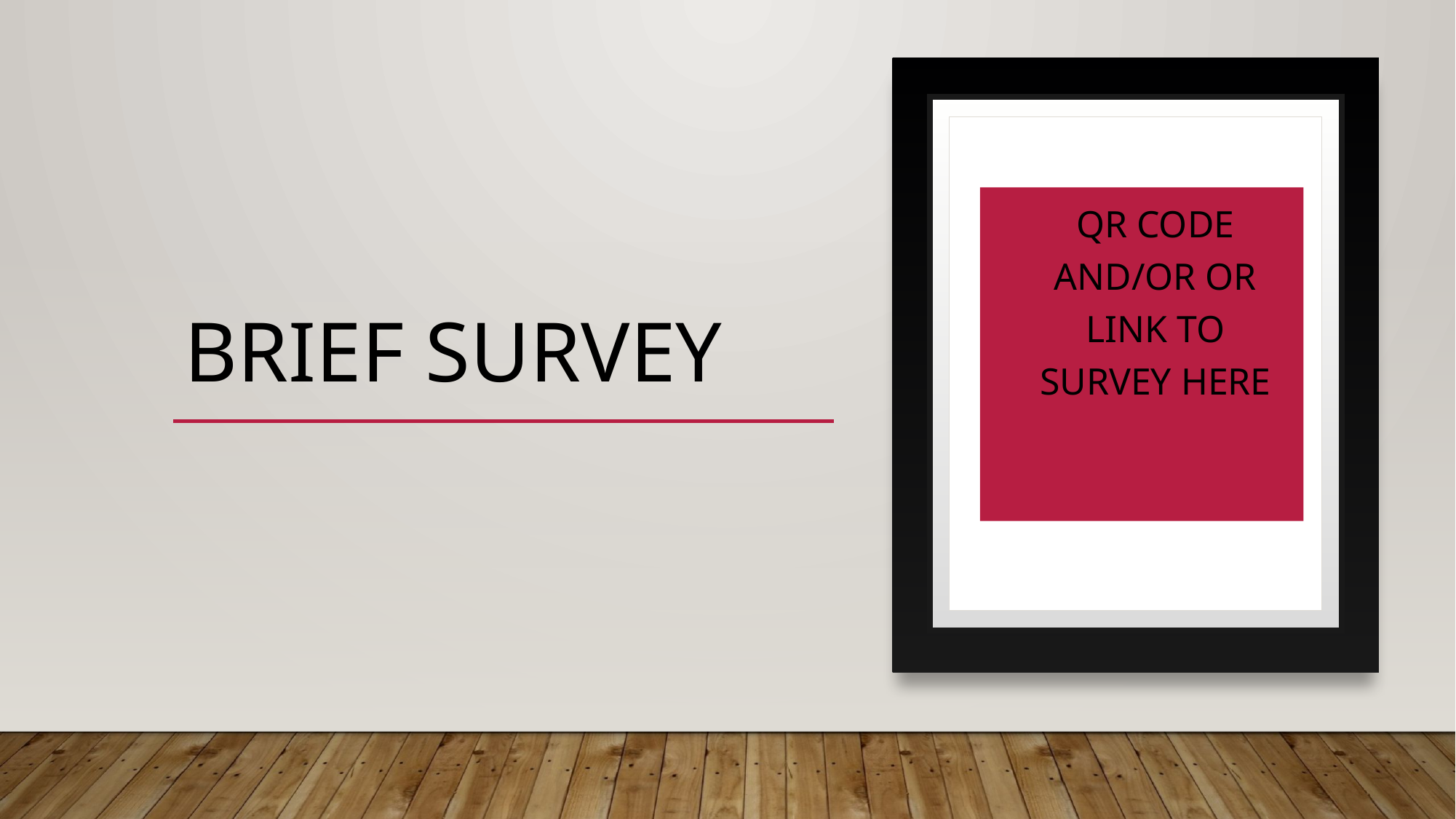

# BRIEF SURVEY
QR CODE AND/OR OR LINK TO SURVEY HERE
