# Supplementary material for: A Resident Narrative Medicine Curriculum to Promote Professional Identity Development: Story-Based Sessions Grounded in Narrative Learning Theory
Source: MedEdPORTAL. 2024 Oct 22;20:11446. doi: 10.15766/mep_2374-8265.11446 (PMC11493853; doi:10.15766/mep_2374-8265.11446)
Supplement: Supplementary file 1 — Facilitator Guide.docxBurnout and Moral Injury.pptxCompassion Fatigue.pptxWorking Through a Pandemic.pptxDifficult Patient.pptxThe New Normal.pptxFinding Meaning.pptxUnpublished Narratives.docxSurvey.docx [file mep_2374-8265.11446-s001.zip › C. Compassion Fatigue.pptx]

## Slide 1
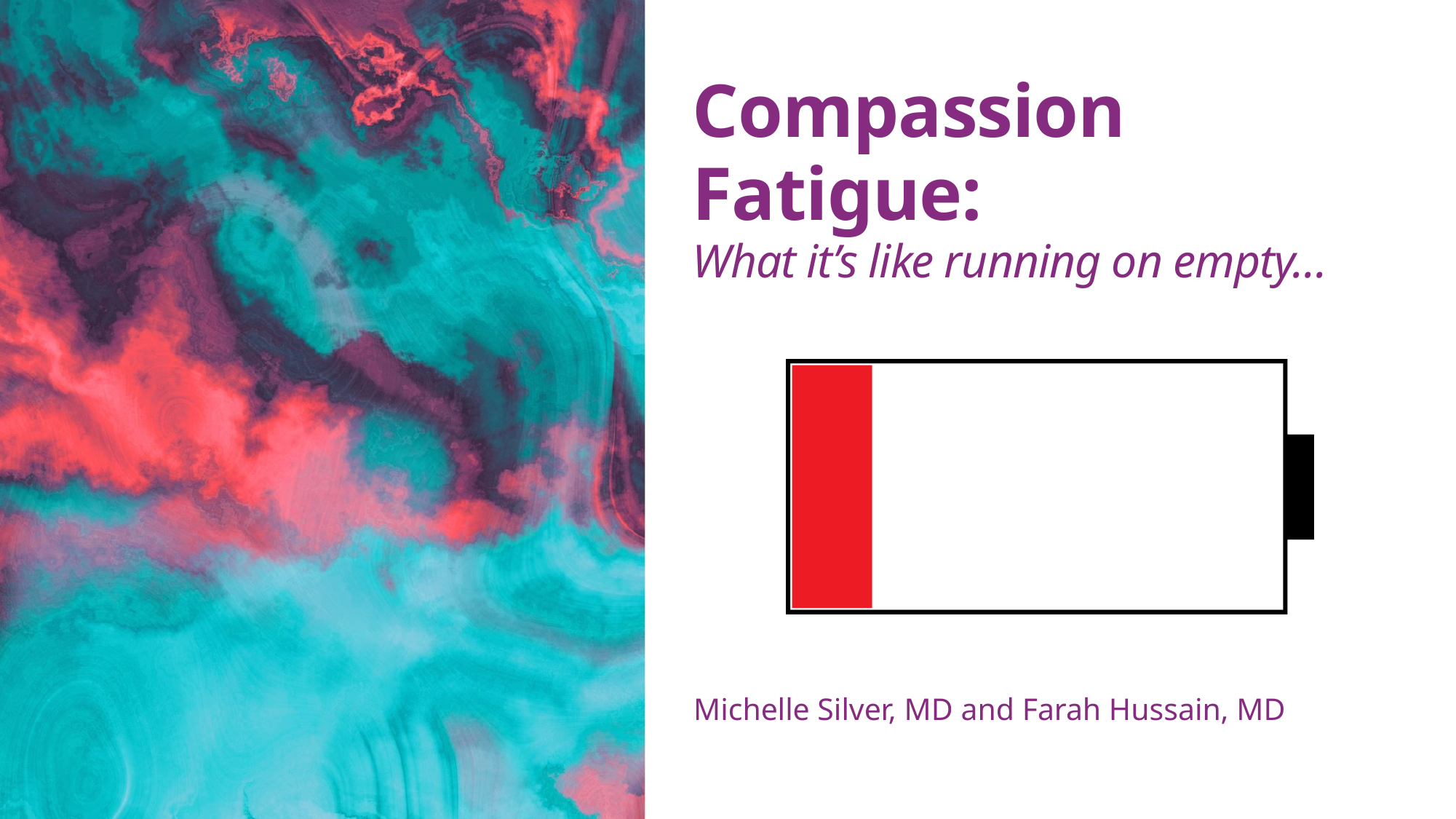

# Compassion Fatigue: What it’s like running on empty…
Michelle Silver, MD and Farah Hussain, MD

## Slide 2
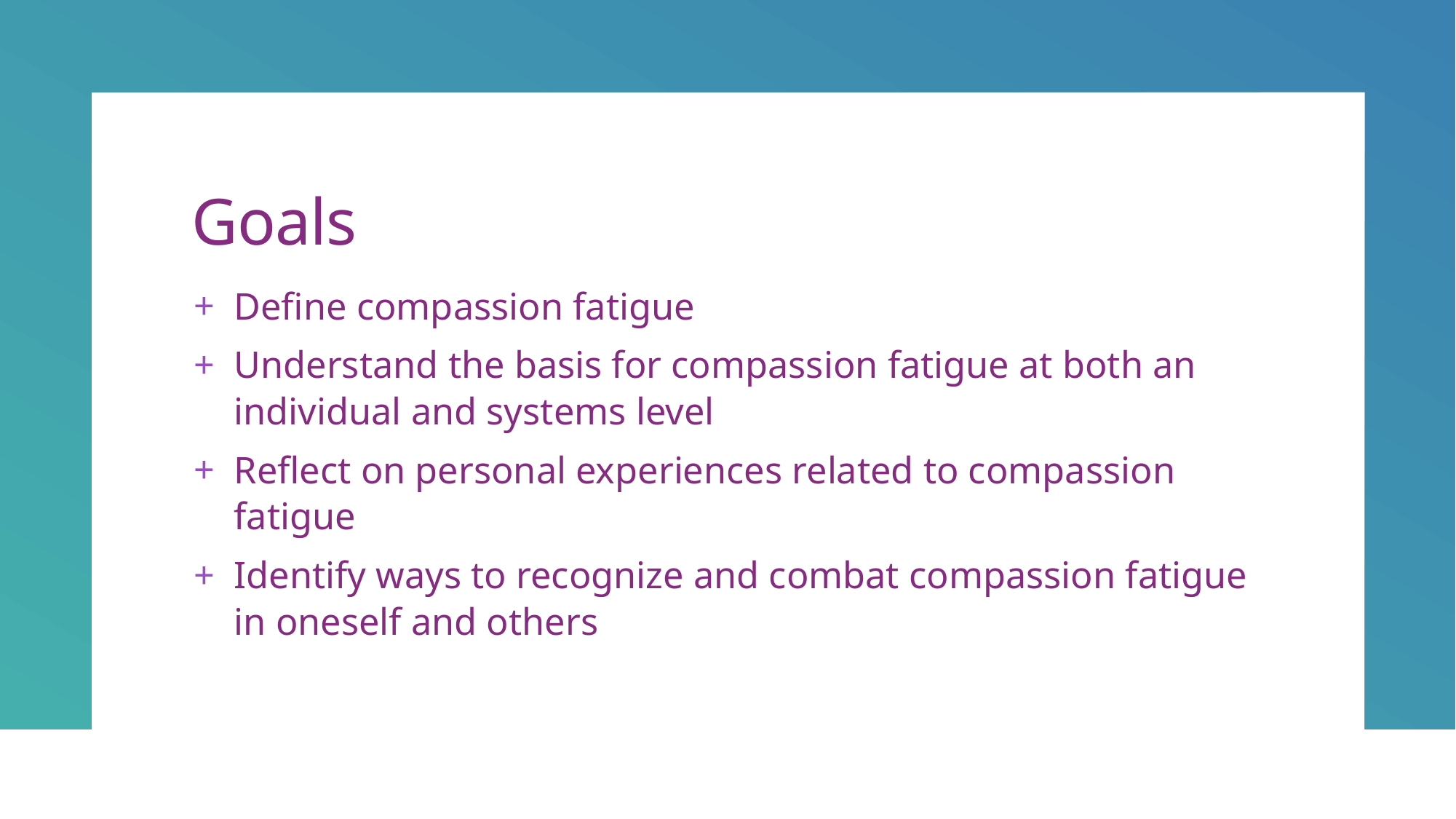

# Goals
Define compassion fatigue
Understand the basis for compassion fatigue at both an individual and systems level
Reflect on personal experiences related to compassion fatigue
Identify ways to recognize and combat compassion fatigue in oneself and others

## Slide 3
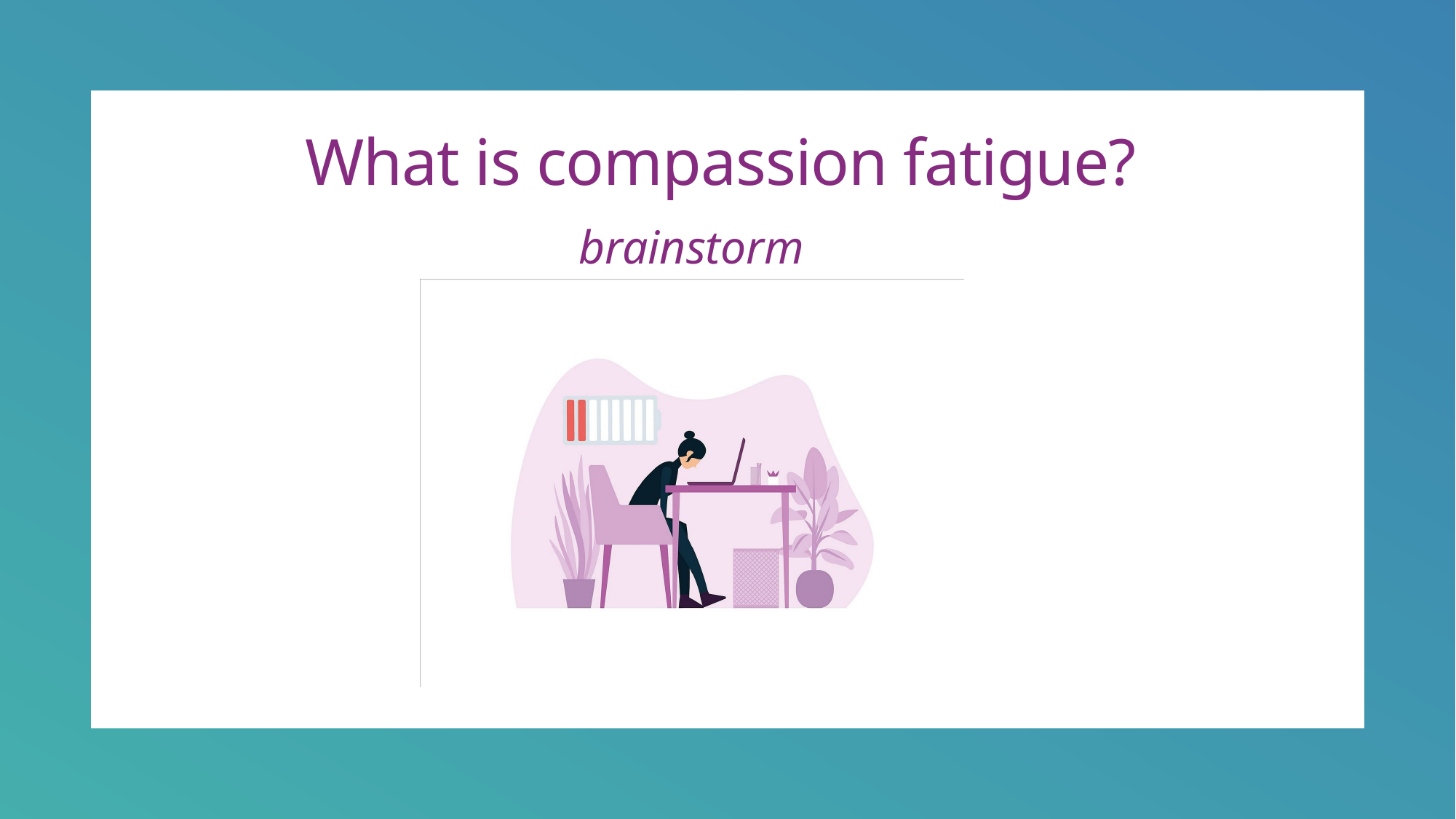

# What is compassion fatigue?
brainstorm

## Slide 4
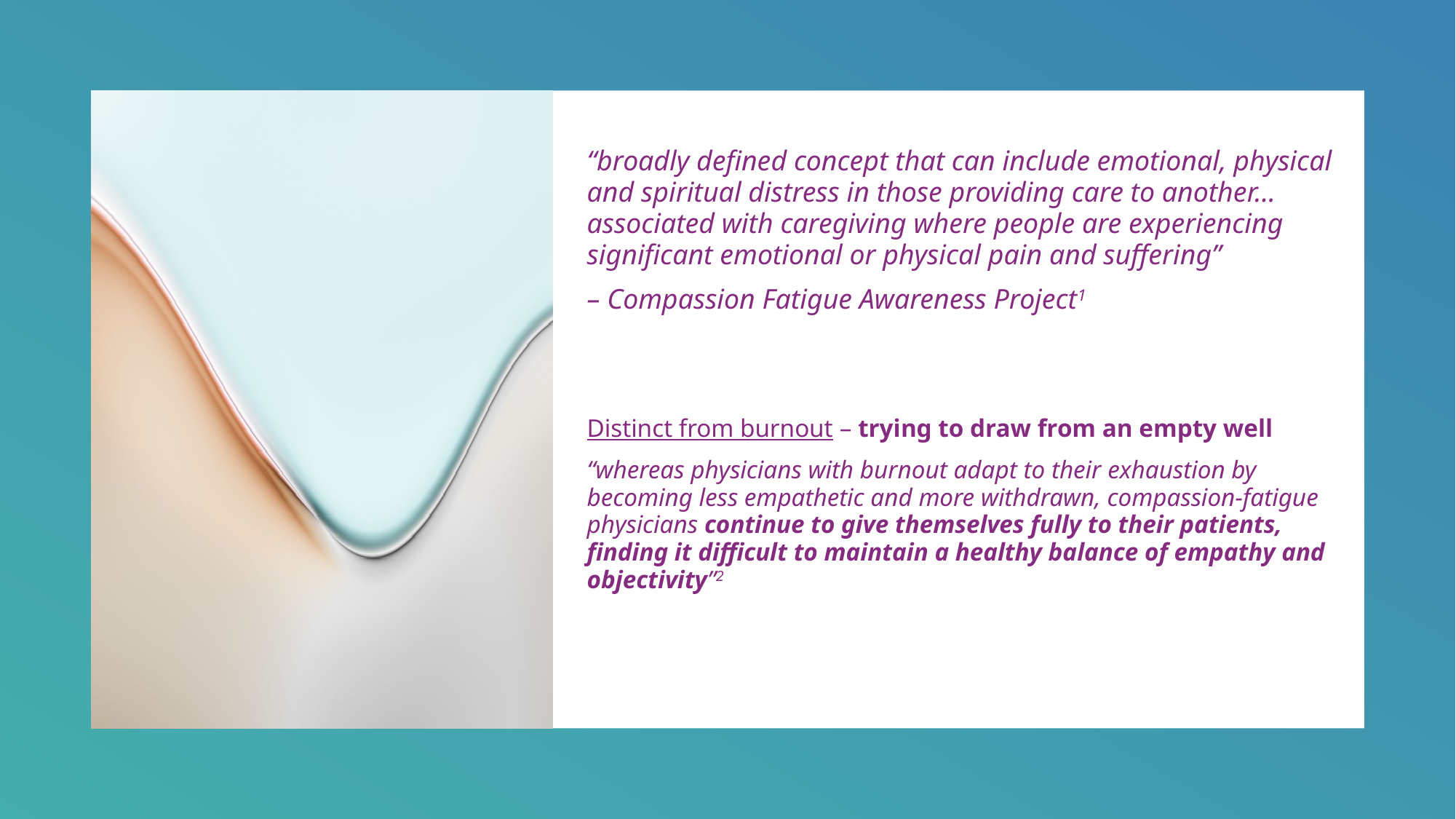

“broadly defined concept that can include emotional, physical and spiritual distress in those providing care to another… associated with caregiving where people are experiencing significant emotional or physical pain and suffering”
– Compassion Fatigue Awareness Project1
Distinct from burnout – trying to draw from an empty well
“whereas physicians with burnout adapt to their exhaustion by becoming less empathetic and more withdrawn, compassion-fatigue physicians continue to give themselves fully to their patients, finding it difficult to maintain a healthy balance of empathy and objectivity”2

## Slide 5
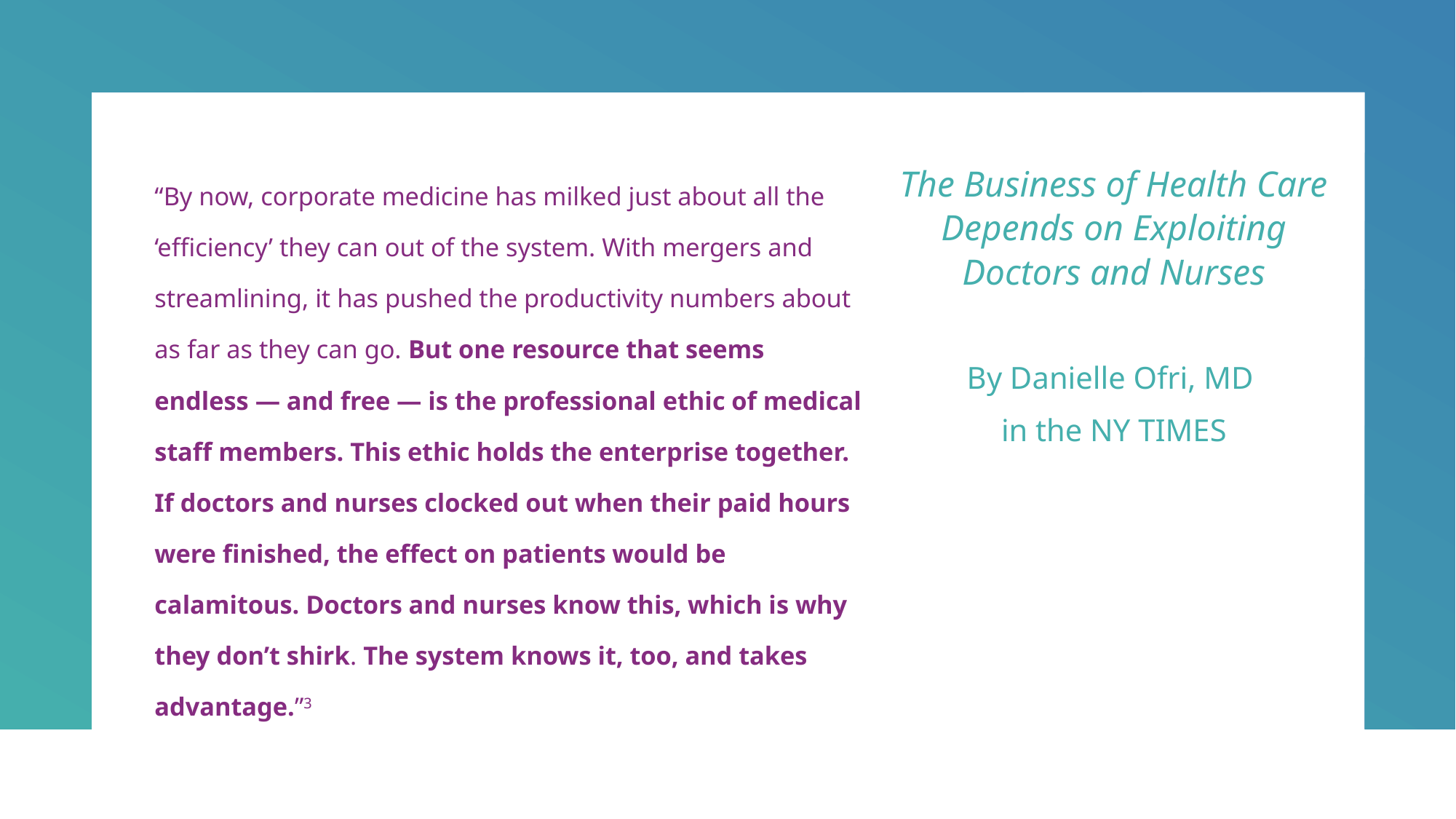

“By now, corporate medicine has milked just about all the ‘efficiency’ they can out of the system. With mergers and streamlining, it has pushed the productivity numbers about as far as they can go. But one resource that seems endless — and free — is the professional ethic of medical staff members. This ethic holds the enterprise together. If doctors and nurses clocked out when their paid hours were finished, the effect on patients would be calamitous. Doctors and nurses know this, which is why they don’t shirk. The system knows it, too, and takes advantage.”3
The Business of Health Care Depends on Exploiting Doctors and Nurses
By Danielle Ofri, MD
in the NY TIMES

## Slide 6
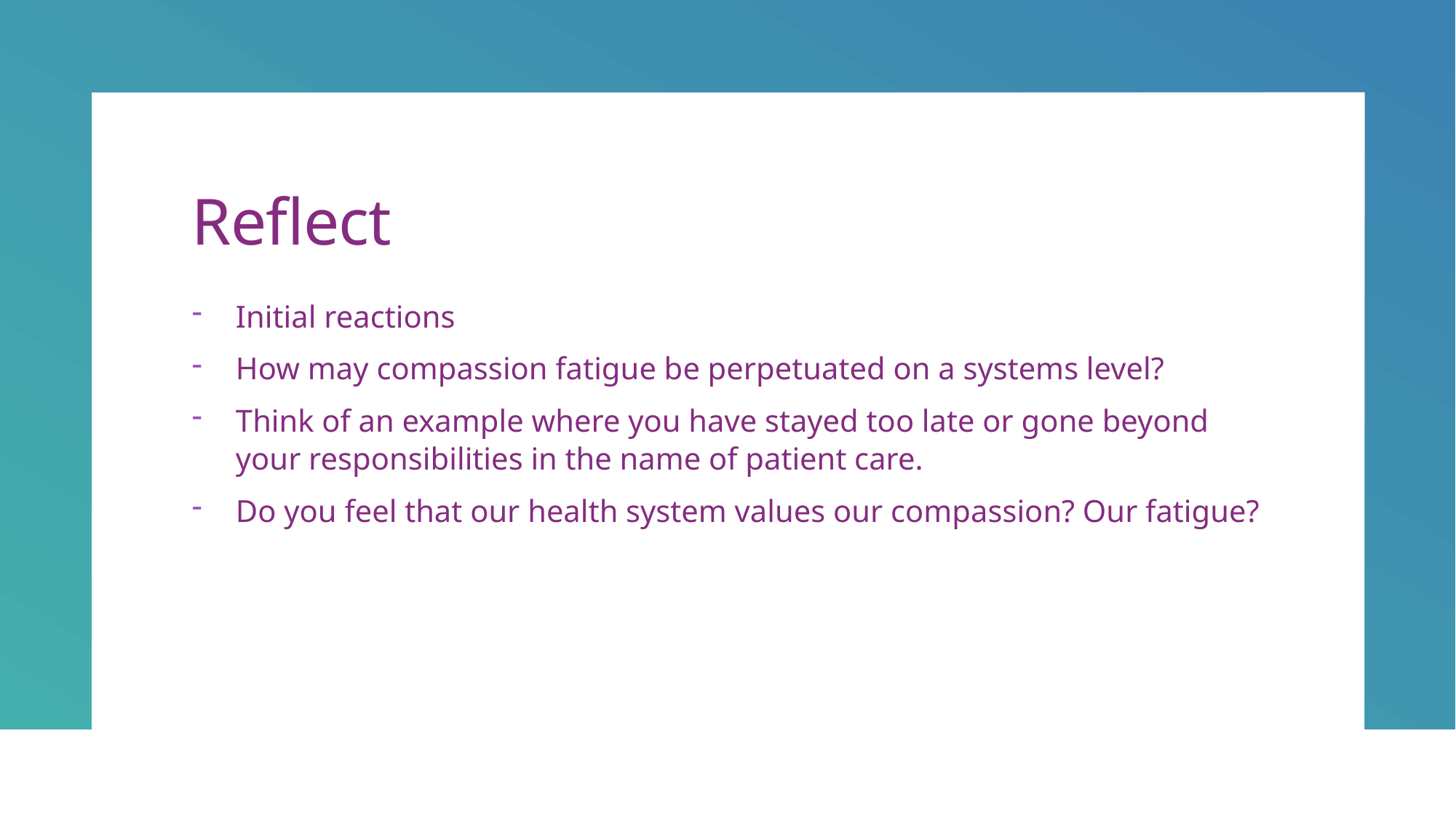

# Reflect
Initial reactions
How may compassion fatigue be perpetuated on a systems level?
Think of an example where you have stayed too late or gone beyond your responsibilities in the name of patient care.
Do you feel that our health system values our compassion? Our fatigue?

## Slide 7
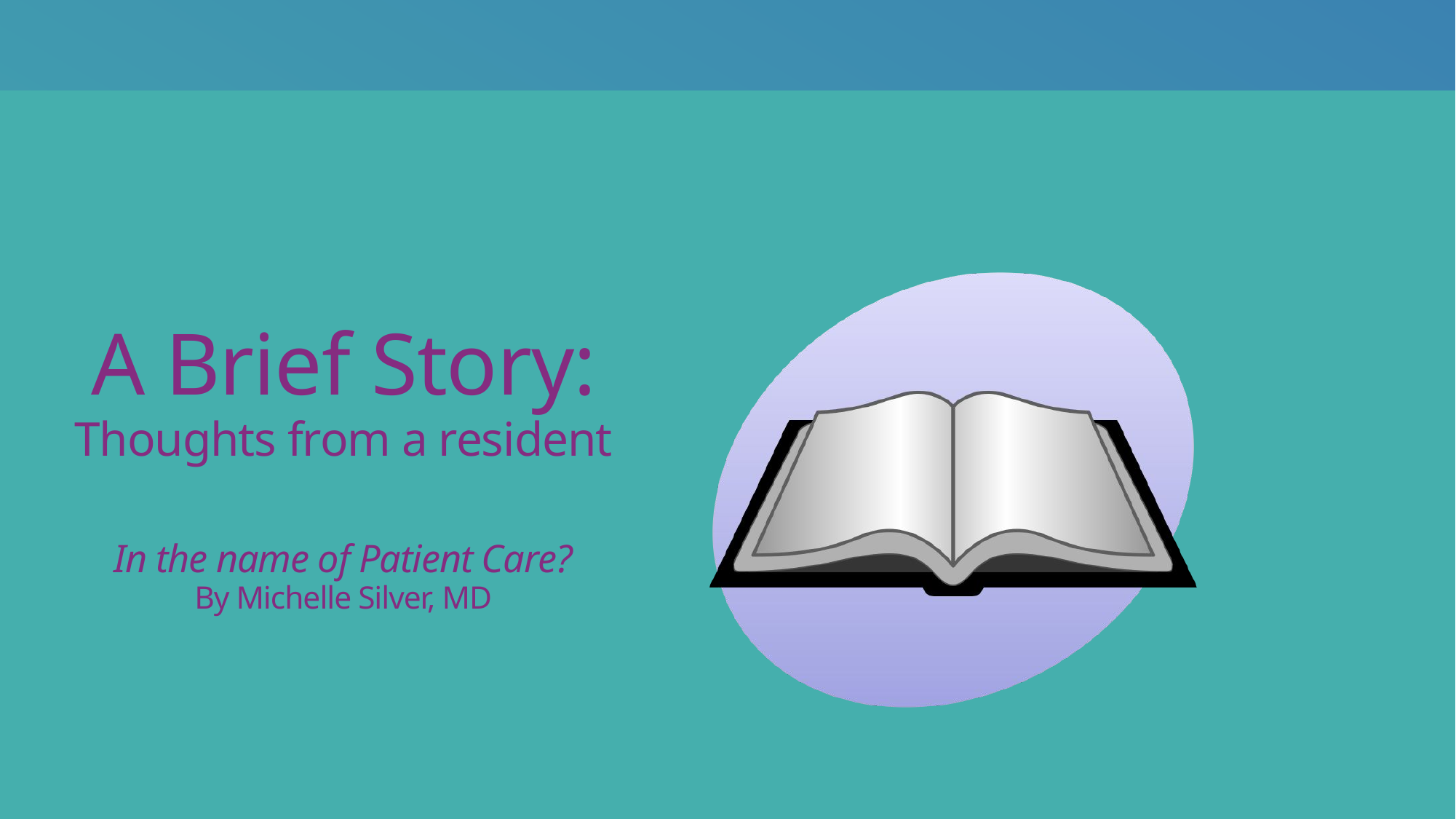

# A Brief Story: Thoughts from a resident In the name of Patient Care? By Michelle Silver, MD

## Slide 8
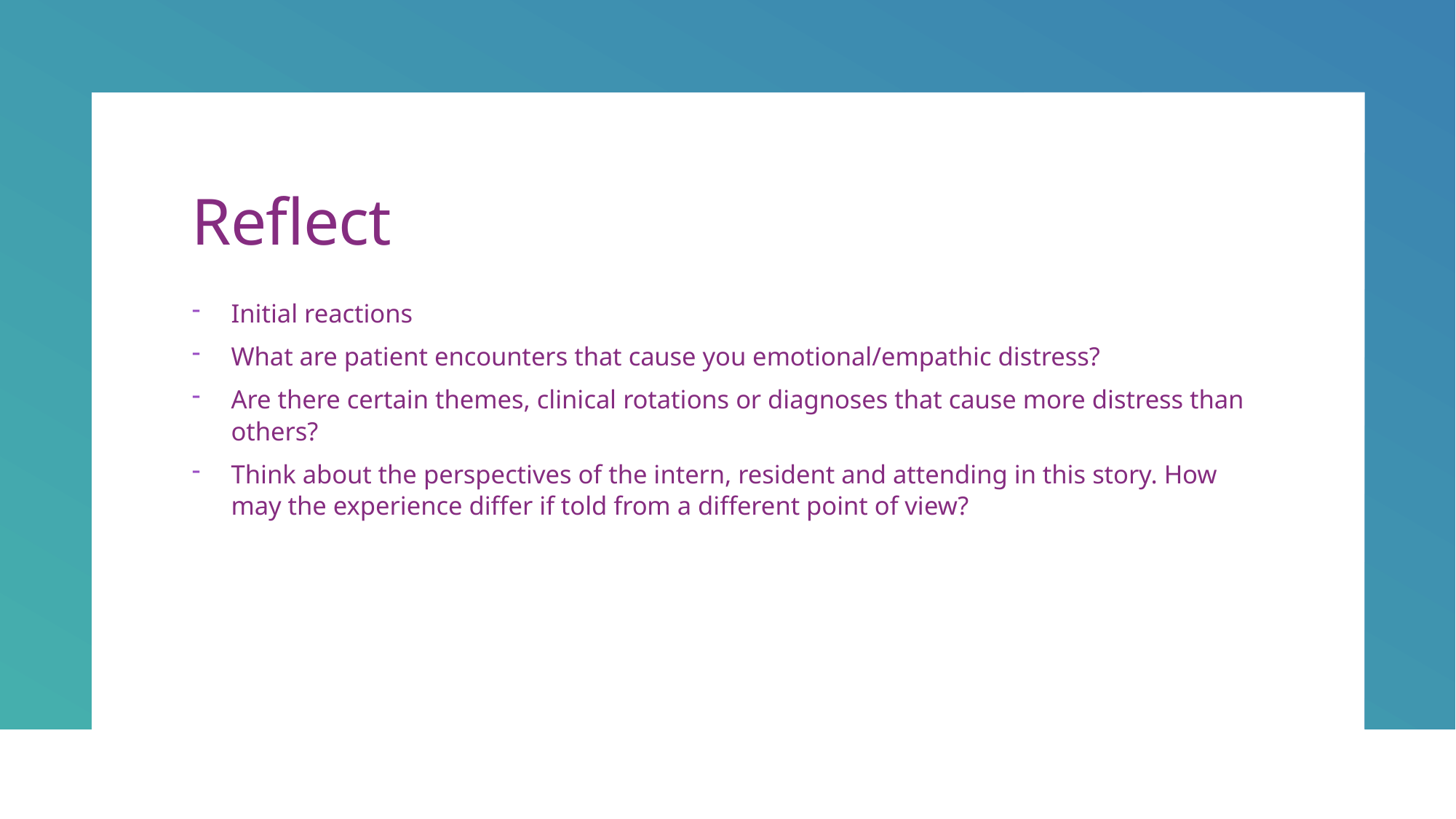

# Reflect
Initial reactions
What are patient encounters that cause you emotional/empathic distress?
Are there certain themes, clinical rotations or diagnoses that cause more distress than others?
Think about the perspectives of the intern, resident and attending in this story. How may the experience differ if told from a different point of view?

## Slide 9
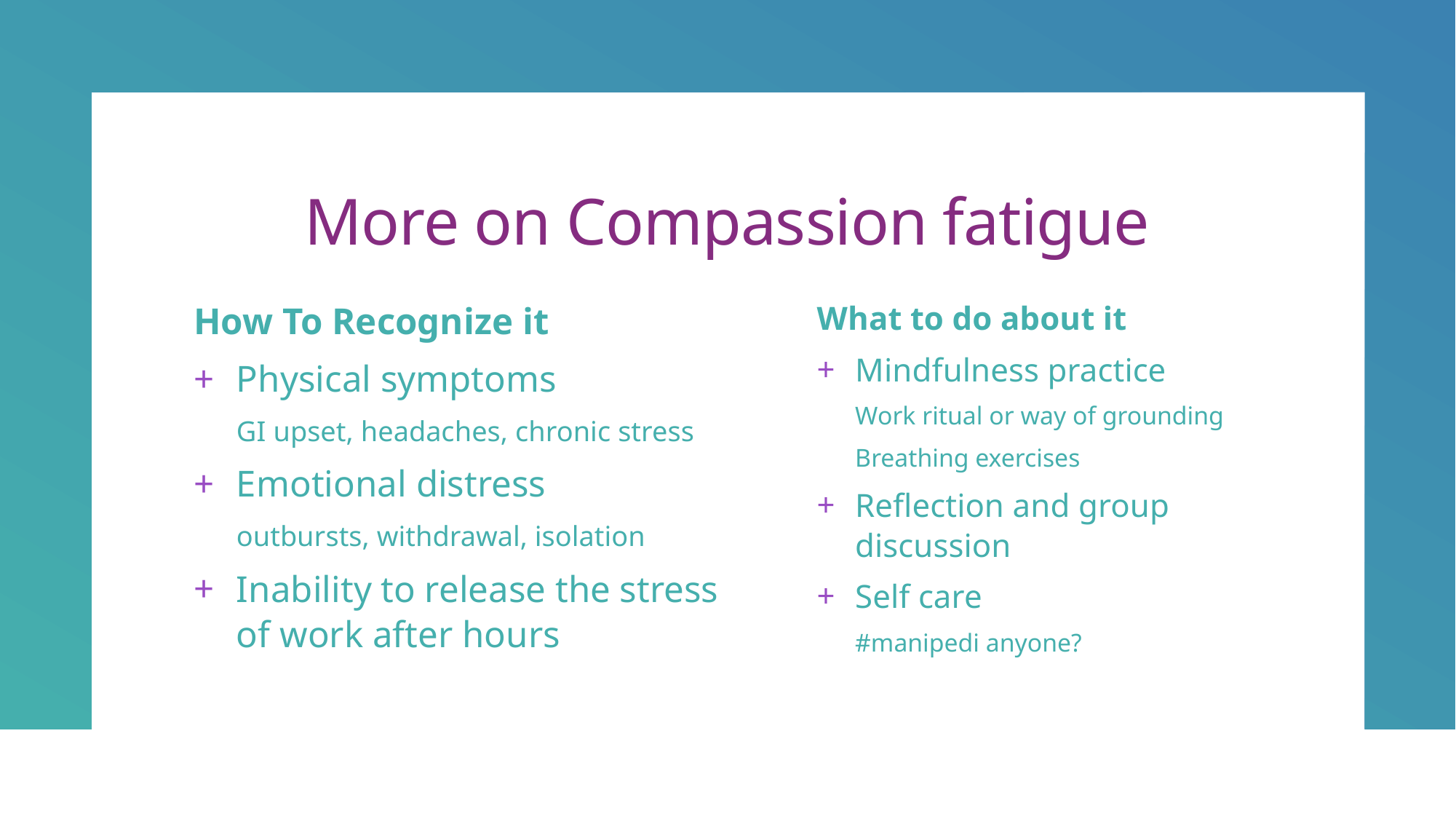

# More on Compassion fatigue
How To Recognize it
Physical symptoms
GI upset, headaches, chronic stress
Emotional distress
outbursts, withdrawal, isolation
Inability to release the stress of work after hours
What to do about it
Mindfulness practice
Work ritual or way of grounding
Breathing exercises
Reflection and group discussion
Self care
#manipedi anyone?

## Slide 10
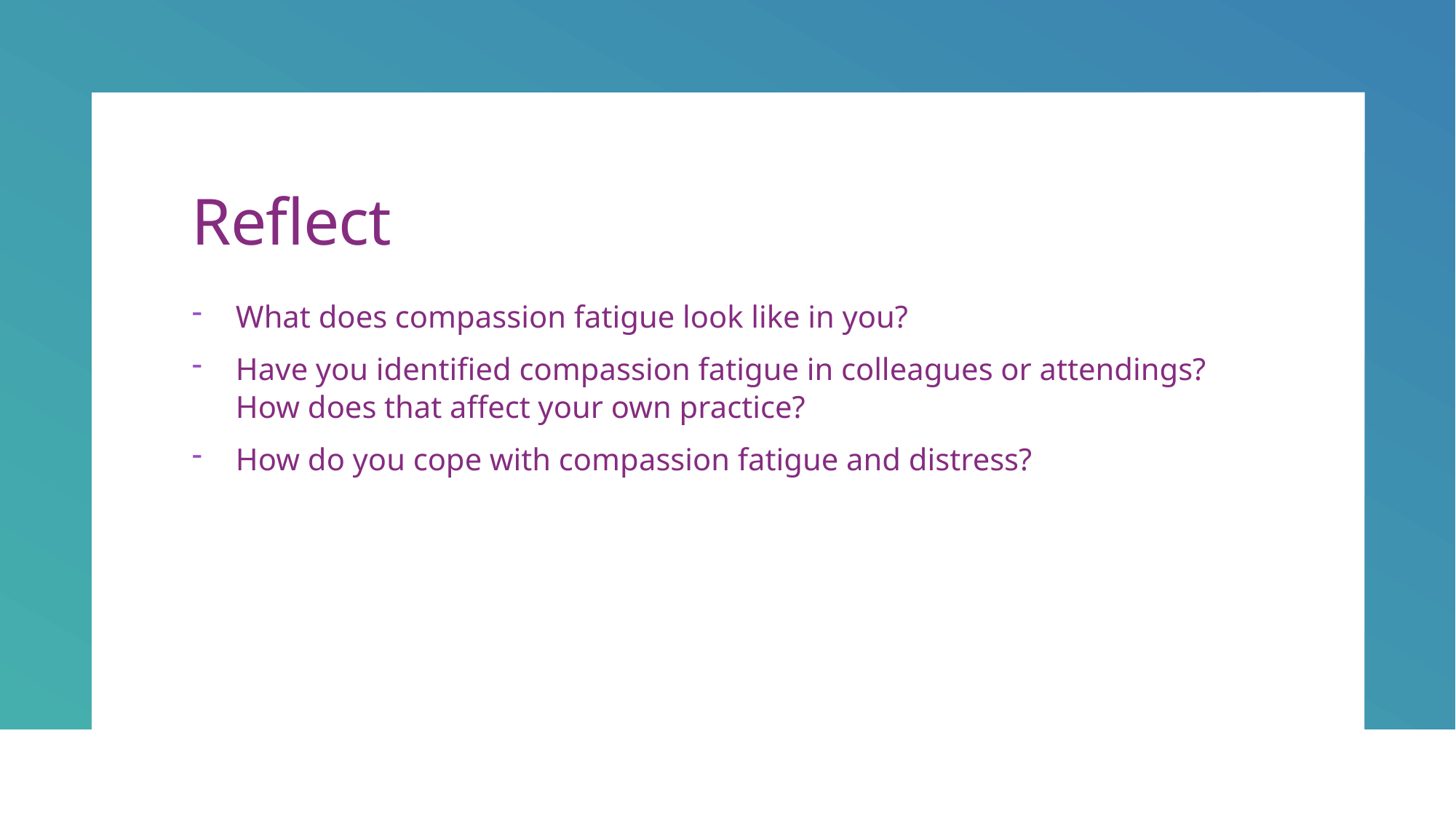

# Reflect
What does compassion fatigue look like in you?
Have you identified compassion fatigue in colleagues or attendings? How does that affect your own practice?
How do you cope with compassion fatigue and distress?

## Slide 11
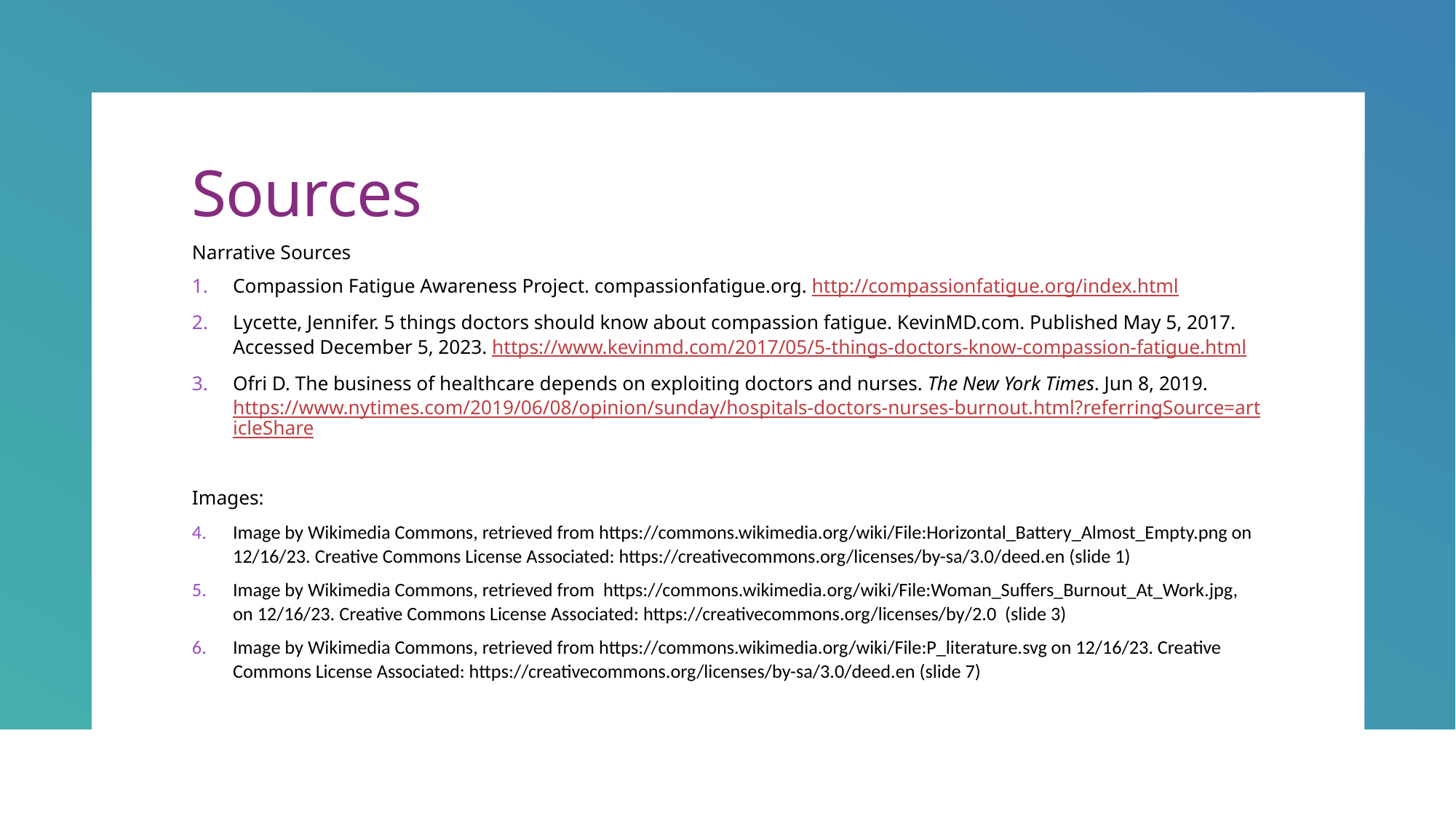

# Sources
Narrative Sources
Compassion Fatigue Awareness Project. compassionfatigue.org. http://compassionfatigue.org/index.html
Lycette, Jennifer. 5 things doctors should know about compassion fatigue. KevinMD.com. Published May 5, 2017. Accessed December 5, 2023. https://www.kevinmd.com/2017/05/5-things-doctors-know-compassion-fatigue.html
Ofri D. The business of healthcare depends on exploiting doctors and nurses. The New York Times. Jun 8, 2019. https://www.nytimes.com/2019/06/08/opinion/sunday/hospitals-doctors-nurses-burnout.html?referringSource=articleShare
Images:
Image by Wikimedia Commons, retrieved from https://commons.wikimedia.org/wiki/File:Horizontal_Battery_Almost_Empty.png on 12/16/23. Creative Commons License Associated: https://creativecommons.org/licenses/by-sa/3.0/deed.en (slide 1)
Image by Wikimedia Commons, retrieved from  https://commons.wikimedia.org/wiki/File:Woman_Suffers_Burnout_At_Work.jpg, on 12/16/23. Creative Commons License Associated: https://creativecommons.org/licenses/by/2.0  (slide 3)
Image by Wikimedia Commons, retrieved from https://commons.wikimedia.org/wiki/File:P_literature.svg on 12/16/23. Creative Commons License Associated: https://creativecommons.org/licenses/by-sa/3.0/deed.en (slide 7)

## Slide 12
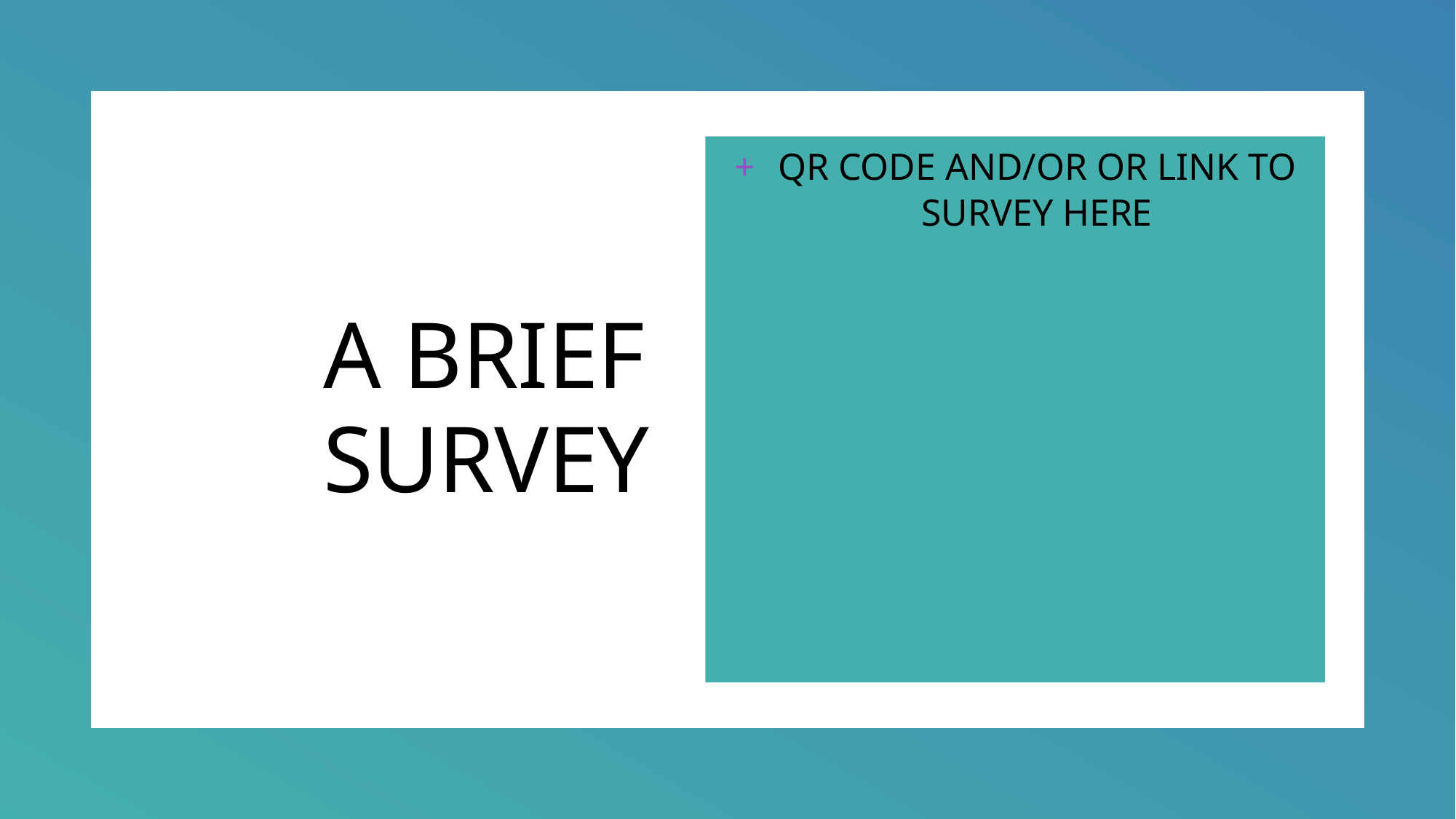

QR CODE AND/OR OR LINK TO SURVEY HERE
# A BRIEF SURVEY
