# Supplementary material for: A Resident Narrative Medicine Curriculum to Promote Professional Identity Development: Story-Based Sessions Grounded in Narrative Learning Theory
Source: MedEdPORTAL. 2024 Oct 22;20:11446. doi: 10.15766/mep_2374-8265.11446 (PMC11493853; doi:10.15766/mep_2374-8265.11446)
Supplement: Supplementary file 1 — Facilitator Guide.docxBurnout and Moral Injury.pptxCompassion Fatigue.pptxWorking Through a Pandemic.pptxDifficult Patient.pptxThe New Normal.pptxFinding Meaning.pptxUnpublished Narratives.docxSurvey.docx [file mep_2374-8265.11446-s001.zip › _Educational Summary Report_11446.pdf]

MedEdPORTAL® The AAMC Journal of Teaching and Learning Resources

Original Publication Open Access

# A Resident Narrative Medicine Curriculum to Promote Professional Identity Development: Story-Based Sessions Grounded in Narrative Learning Theory

Michelle Silver, MD*, Farah Hussain, MD

*Corresponding author: michelle.silver8@gmail.com

## Abstract

**Introduction:** Empathy, reflection, and social connectedness are important skills for physician identity development and are increasingly challenged by burnout. Humanities-based interventions like narrative medicine (NM) are emerging in medical education to promote these skills. Only 17% of such initiatives target graduate medical learners. Furthermore, interventions are inconsistent in approach and theory representation. NM uses story-based learning to promote reflection and group discussion. Inspired by narrative learning theory, we developed NM sessions for residents to foster healthy identity development. **Methods:** Ninety-minute sessions were integrated into curricula for PGY 1-PGY 3 internal medicine–primary care residents at two large academic centers. Sessions involved engagement with a narrative source (stories, poems, art), personal reflection, and group discussion. Topics ranged from burnout to difficult patients. Participants completed anonymous postsession surveys assessing satisfaction, attitudes, and skills. **Results:** Fifteen sessions occurred from 2021 to 2023, with three to 10 residents per session. Fifty residents completed between one and four sessions, with 68 survey responses (response rate: 88%). Over 95% ranked 4-5 out of 5 for overall impression ($n = 67$) and personal value ($n = 65$) of sessions. Sessions were highly enjoyable ($M = 4.8$), with mean scores of $>4$ out of 5 for impact on wellness, appreciation of work values, social connectedness, and patient care. **Discussion:** NM sessions demonstrated measurable improvements in several domains of professional performance including wellness, job satisfaction, and patient care, while promoting camaraderie and emotion processing. Our materials offer tremendous potential for promoting healthy identity formation.

### Keywords
Burnout, Empathy, Narrative Learning Theory, Wellness, Humanities (Art, Literature, Music), Professional Identity Formation, Professionalism, Reflection/Narrative Medicine, Well-Being/Mental Health

## Educational Objectives

By the end of this activity, learners will be able to:

1. Interpret and discuss literary works as they relate to personal experiences.
2. Recognize the challenges and formative experiences universal to medical training.
3. Analyze and articulate challenging work experiences through storytelling.
4. Examine how challenging work experiences inform their professional identity.
5. Apply shared reflection and emotion processing to improve social connection with colleagues.

**Citation:**
Silver M, Hussain F. A resident narrative medicine curriculum to promote professional identity development: story-based sessions grounded in narrative learning theory. *MedEdPORTAL*. 2024;20:11446. https://doi.org/10.15766/mep_2374-8265.11446

## Introduction

Empathy, capacity for reflection, and social connectedness are important skills for becoming a compassionate and professional physician. These attributes play a crucial role in professional identity development and are increasingly challenged in modern medicine by the evolving burnout epidemic.[1,2] Therefore, these skills are essential curricular targets in medical education.[1] The arts and humanities are becoming widely incorporated in medical education to enhance professional identity formation, encourage reflective practice, and promote interpersonal connections.[3,4] Academic literature began reporting on the use of literature and the humanities in medical education in the 1970s, which, by the end of the 20th century, led to more publications explicitly directed toward clinical audiences and a clearer definition of one such intervention, narrative medicine (NM).[5] NM is an application

Copyright © 2024 Silver and Hussain This is an open-access publication distributed under the terms of the Creative Commons Attribution license. 

of arts and humanities that utilizes literature and art to promote creative reflection and group discussion around stories pertinent to medical training. This approach of narrative competence—the ability to acknowledge, interpret, and act on stories—has become a pedagogic strategy for cultivating growth in medical trainees via personal insight, as well as skills to articulate formative work experiences.[6,7] In one such example, NM has been demonstrated to positively impact professional identity development in medical students.[3]

While utilization of arts and humanities is increasing, it is more commonly implemented at the level of undergraduate medical education (UME), as opposed to graduate medical education (GME). Only 17% of published arts and humanities initiatives target the GME population, despite this being a crucial time for the development of professional identity as well as a high-risk time for burnout and declining empathy.[2,8-10] Existing publications on NM interventions in GME learners are pilot studies limited in scope. Data typically represent 10-30 learners and range in assessment from surveys to semi-structured interviews and focus groups. Examples include a voluntary NM intervention in pediatric residents, mandatory NM seminars for PGY 1 surgery residents, a graphic design workshop for neurology residents, and a voluntary NM workshop for internal medicine residents.[11-14] When implemented, data continue to support positive effect on learner well-being but otherwise vary in targeted outcomes. Identified barriers to implementing such a curriculum at the GME level include (1) limitations in time and funding for nonclinical education, (2) the prioritization of other nonclinical activities such as research and QI on fellowship and job applications, and (3) the lack of cultural or structural support.[15] Many of these barriers can be circumvented by using existing mandatory didactic time and a structured, evidence-based approach. Therefore, there is a dire need for more evidence to promote cultural buy-in at the GME level.

In addition to lacking GME representation, arts and humanities publications are widely inconsistent in approach and theory representation. Only 30% of publications explicitly frame theories, and only 11% utilize NM, according to a recent scoping review commissioned by the Association of American Medical Colleges.[8] While newly emerging data support the role of NM in professional identity formation in medical trainees, narrative theory and narrative learning are historic and widely accepted psychological theories linked to identity formation in adulthood.[16] According to narrative theory, human existence is grounded in the use of stories to make meaning of experiences—and how people craft these stories is intimately tied to their identities. Narrative learning is based in constructivist learning theory and is the process by which meaning is made. Storytelling is a complex adult learning process that appeals to all aspects of humanity from concrete to imaginative to emotional and promotes higher-level processing. More aptly put, "the construction of the narrative is necessary to make the experience accessible (that is, to language it), and how it is constructed determines what meaning it has for the person."[16] Therefore, by aligning our resource with the application of narrative learning methods, we hope to better define the role of NM in medical education, providing a centralized framework for others to build from when creating both session content and survey or interview design. Furthermore, our tools offer consistency in how these sessions are structured and specifically target NM's role in professional identity development in GME learners.

Residency is a uniquely formative period of medical training full of many challenging, emotionally intense, and unfamiliar stories. We know that during this time, trainees show worsening self-esteem, declining empathy, and increased burnout.[9,10] Narrative learning theory suggests storytelling may have tremendous potential in combating such decline by promoting positive identity development through processing and articulating lived experiences. Providing intentional time and space to construct narratives around challenging experiences can promote healthy identity formation and target burnout amongst this high-risk population.

In the current educational resource, we introduced a novel NM curriculum for medicine residents centered around stories universal to trainee experiences. We implemented sessions during mandatory didactic time for internal medicine–primary care residents in their PGY 1-PGY 3 years at two large urban academic institutions from 2021 through 2023. Sessions were built into a yearlong curriculum but also could be stand-alone. Session facilitators had experiential expertise leading NM and wellness initiatives. Session structure was adapted from an evidence-based NM curriculum that had primarily been implemented at the UME level.[17,18] Surveys administered at the end of each session were designed to evaluate participant reactions, as well as perceived impact on wellness and professional identity development.

## Methods

Setting and Population

Participants included residents in the primary care tracks of internal medicine residency programs at two large urban academic institutions: Hospital of the University of Pennsylvania

Copyright © 2024 Silver and Hussain This is an open-access publication distributed under the terms of the Creative Commons Attribution license.

(Penn) and Beth Israel Deaconess Medical Center (BIDMC). NM sessions were embedded in the primary care curriculum, occurring during mandatory didactic time in preassigned clinic cohorts. Cohorts included residents across all three training years. We initially held sessions virtually due to required precautions related to the COVID-19 pandemic, with transition to in-person sessions by early 2022 per hospital policy. Each session included three to 10 resident participants and one to two facilitators. Facilitators included trusted core faculty members. Penn began this curriculum in January 2021. After one author joined a new institution (Michelle Silver), we began the same sessions, in parallel, at BIDMC starting in November 2022.

Facilitators
Facilitators for all sessions included one internal medicine faculty member (Farah Hussain and later Michelle Silver) and the resident leader (Michelle Silver) for the project. As facilitators, we had experiential expertise leading NM and wellness sessions both during residency and as attendings. This involved observing skills from senior mentors with expertise in areas such as positive psychology, mindfulness techniques, and Balint groups, to gain experience with fostering safe spaces to promote resident engagement and vulnerability. After several opportunities for observation, one faculty facilitator (Farah Hussain) led small-group sessions within the internal medicine residency's emotional intelligence curriculum to gain expertise in cultivating compassionate environments to encourage sharing and vulnerability.

Skills recommended for effective facilitators include (1) experience with positive psychology and NM techniques, (2) experience with trauma-informed care, (3) a position as a trusted member of the core faculty (through mentorship or other leadership opportunities) while not holding any evaluative roles, and (4) the ability to incorporate compassionate and empathetic discussion techniques to ensure psychological and emotional safety during the sessions included in the curriculum. For additional guidance, please see the facilitator guide (Appendix A).

Sessions
*Schedule:* We ran 15 ninety-minute sessions from January 2021 through September 2023 across both institutions. Sessions were held during protected mandatory didactic time. At each institution, the internal medicine–primary care residents were divided into two cohorts; sessions were conducted twice, once for each cohort. Frequency of sessions varied by institution due to differences in scheduling. At site 1, each cohort had 8-12 weeks between sessions and averaged three sessions per academic year. At site 2, each cohort also averaged three sessions per academic year and completed sessions every 2 months within a 6-month period.

*Session structure:* We adapted sessions from an evidence-based NM curriculum to follow the same organization: (1) reflective engagement with narrative, literary, or art sources; (2) personal reflection on the source; and (3) sharing and discussion in a monitored, supportive environment.[17,18] Each session was designed around a formative topic universal to internal medicine training. Themes were selected in response to informal focus groups with residents in each program, to represent their prioritized topics. After the presentation of a story or art source, we provided discussion questions to aid in personal reflection, with dedicated time to process responses and the option to write them down. There were no preassigned readings. We created PowerPoint presentations to facilitate each session, including sources and discussion questions (Appendices B-G). Sources for each session included poems, stories, and art, the majority of which had been published, as well as some unpublished sources created by peers internally (Appendix H). For the last 5-10 minutes of each session, we allotted time for participants to complete an optional online survey (Appendix I).

*Session themes:* Session themes included the following:

1. Burnout and moral injury.
2. Compassion fatigue: What running on empty is like.
3. Working through a pandemic: What does it mean to be called a health care hero?
4. Difficult patients.
5. The new normal: residency 2 years into a pandemic.
6. Finding meaning in medicine.

*Session layout:* For Zoom sessions, we encouraged participants to have their cameras on for the entirety of the session and to view images of all participants, as opposed to presenter view, to promote a sense of community. We held in-person sessions in a small conference room with participants seated around a large table. All sessions followed a PowerPoint for audiovisual assistance that displayed the session title, literary sources, and discussion prompts. Workshops were facilitated by one or both members of the educational session team, which included a trainee within the primary care program at Penn, transitioned to faculty in the Department of Medicine at BIDMC (Michelle Silver), and a faculty member in the Department of Medicine at Penn (Farah Hussain). We have provided guidance for session facilitation and best practices in a facilitator guide (Appendix A). We recommend reviewing this guide prior to starting any

Copyright © 2024 Silver and Hussain This is an open-access publication distributed under the terms of the Creative Commons Attribution license.

session in order to become familiar with best practices. We also recommend reviewing the survey (Appendix I) to guide targeted evaluation measures, keeping these in mind as sessions are designed and facilitated.

### Data Acquisition and Analysis

We provided learners with a link and QR code to the anonymous survey at the end of each NM session (Appendix I). They were allotted 5-10 minutes, away from facilitators, to complete it. The survey included three open-ended questions and 14 items scored on 5-point Likert scales (1 = *negative* or *not useful*, 5 = *greatly positive* or *extremely useful*). We developed the survey based on learning objectives and previously identified attitudes and skills necessary for professional identity formation. Given the variability and inconsistency with which NM interventions have been evaluated, the survey was not based on prior publications. Open-ended questions addressed perceived purpose of the session, overall impression, and feedback. Likert-scale questions ranged from sense of wellness and enjoyment from the session to its impact on job satisfaction, patient care, and connectivity with coresidents. We presented descriptive summary statistics for each of our outcomes. Prior to implementation, the survey was approved for use, under the exempt category, by the institutional review boards independently at Hospital of the University of Pennsylvania (protocol number 845017) and Beth Israel Deaconess Medical Center (protocol number 2022P000722).

## Results

We implemented NM sessions for a total of 50 residents over the course of three academic years (2021-2023) at two academic institutions, including internal medicine–primary care residents in the PGY 1-PGY 3 years. Residents participated in at least one session as part of their mandatory didactic time, with the option to complete a survey at the end of each session. Participants averaged two to three sessions total. Participants completed the survey after each session they were a part of, meaning some participants completed the survey multiple times. A total of 15 sessions took place, resulting in 68 survey responses. Average survey response rate was 88%.

Survey data were both qualitative (open-ended responses) and quantitative (through Likert-scale questions). Based on the Kirkpatrick model of evaluation,[19] our evaluation was limited to reaction and self-reported learning in relation to attitude, confidence, and perceived skills acquired after each session.

### Qualitative Data

In a content analysis of open responses, an appreciation of a safe space for emotional processing emerged as a theme. When asked why sessions were part of their training, multiple participants said to "prevent burnout" and the terms *wellness, reflection, resilience*, and *humanity* were used numerous times. There were comments about the catharsis of group discussion and the ability to articulate and process challenging experiences and its positive impact on patient care. When asked for their general impressions, multiple residents used the words *fantastic, positive*, and *important* and emphasized value in connecting with coresidents. Lastly, when asked for areas of improvement, few comments were provided, but themes centered around providing more diverse representation with reading and media, such as outside of home institution and considering a nonphysician perspective; having more group discussion and fewer writing prompts; trying to find time during inpatient rotations for these sessions; and finding ways to incorporate more sessions throughout the year and expand to non–primary care residents. See the Figure for notable quotations.

### Quantitative Data

On average, responses to all 5-point Likert-scale questions were rated as positive ($M > 3.0$), as opposed to neutral (3.0) or negative (<3.0). Over 95% of responses ranked 4-5 out of 5 for overall impression ($n = 67$ out of 68) and personal value ($n = 65$ out of 68). Sessions were rated as being highly enjoyable ($M = 4.8$) and personally valuable (4.7), with a high overall impression (4.7). Residents reported positive impact on wellness (4.1), appreciation of work values (4.2), and job satisfaction (3.9). They also reported improvement in professional skills including connectedness with coresidents (4.6), ability to articulate patient encounters (4.2), and impact on patient care (4.2). Group discussions were rated as the most useful component of sessions (4.9) in comparison to reading/source review (4.5), and a majority of residents did not engage in the optional writing component.

## Discussion

An NM curriculum adapted to internal medicine residents and aimed at processing formative training experiences demonstrated strongly favorable survey results, with measurable improvements in several domains of professional performance. Residents reported personal enjoyment and value from each session and identified the emergence of many attitudes and skills crucial to professional identity development including job satisfaction, peer camaraderie, and impact on patient care. Open-ended responses highlighted wellness, resilience, and the prevention of burnout as key takeaways while identifying the importance of a safe space for emotion processing and improved understanding of challenging work encounters. Regarding Likert-scale questions, responses skewed strongly positive for

Copyright © 2024 Silver and Hussain This is an open-access publication distributed under the terms of the Creative Commons Attribution license.

| Why These Sessions? | Overall Impression |
|---|---|
| "To help us navigate the emotional complexities of our work and to better equip us personally and professionally to have meaningful careers."<br><br>"To develop a framework outside of the context of clinical care to reflect on learning, practice, and care of patients, and to better articulate my own experiences and thoughts."<br><br>"To help process our experiences as physicians to improve our wellness as well as experiences with our patients."<br><br>"It is important to discuss experiences unique to medicine with colleagues . . . to process and heal from the experiences."<br><br>"To reflect on past experiences and articulate them."<br><br>"They are ways in which we can reconnect with the aspect of us that make us human and realize the barriers towards empathy to ourselves and others."<br><br>"A way to ground us . . . and find meaning as humans and doctors. They are problem solving and hope identifying."<br><br>"They help us understand how to deal with internal and interpersonal incongruence and identify how to remain human."<br><br>"Reflect on experiences and the impact on our training."<br><br>"Reconnect with the meaning of my job."<br><br>"Improving wellness, help with avoiding future burnout but learning processing skills early in training." | "It was very cathartic to talk with my co-residents and hear that they are experiencing similar feelings as me towards patients and residency overall."<br><br>"It was fantastic. I really enjoyed speaking with other residents about how we identify tough situations."<br><br>"It was fantastic. I really appreciate the combination of discussion and prompts, which allow better response and openness than asking 'how are you'."<br><br>"Very helpful investment in my education and wellbeing."<br><br>"Highly positive—excellent session with a highly relevant topic."<br><br>"Allowed me to feel not alone in my experiences."<br><br>"Very nice to have a platform and structure to discuss our reactions."<br><br>"Amazing, welcoming environment with great material."<br><br>"Phenomenal discussion."<br><br>"Helped me cultivate gratitude and empathy."<br><br>"I feel like we need about 100 sessions."<br><br>"An incredible joy." |

**Figure.** Selection of quotations from survey responses provided by residents at the end of each session when asked (1) why these sessions were part of their training and (2) their overall impression of the sessions.

perceived impact on peer connectedness, ability to articulate patient encounters, and patient care. There was a weaker but still positive response regarding job satisfaction. These findings support the theory of narrative learning, where storytelling is crucial in how people learn, find meaning, and form identities.

This resource comes at a vulnerable time in medical training, as residents newly face a large volume of challenging, emotionally intense, and unfamiliar stories. As a result, residents demonstrate higher rates of burnout and declining empathy throughout their training.[9,10] These NM sessions can be an effective way to target this, with dedicated time and space to reflect on topics important for identity development that are not readily addressed in other aspects of their training—as evidenced by the lack of humanities and reflection-based curricular interventions that exist at the GME level. Our results offer hope that an evidence-based NM

Copyright © 2024 Silver and Hussain This is an open-access publication distributed under the terms of the Creative Commons Attribution license. 

approach, grounded in narrative learning theory, provides a solution to this gap.

Within sessions, residents preferred group discussion over writing or individual reflection. We suspect this was due to a lack of experience and comfort with the latter. In fact, a majority of residents chose not to participate in the optional writing component, and many expressed an appreciation for discussion questions to assist in reflection. With increased exposure to NM, we are hopeful of addressing this gap in the skill set. Our pilot curriculum is intended to expand and take place over multiple years, with the ability to recycle material with resident turnover, but also to adapt session themes in response to evolving priorities.

Skillful faculty facilitation is key in promoting resident engagement during NM sessions. It is important that facilitators have experience and comfort in the medical humanities to aid in interpretation of the literary sources. Furthermore, they require communication skills and empathy to create a safe and open learning environment. We acknowledge that openness and level of engagement during these sessions likely led to the exceptionally high ratings on the postsession survey. Therefore, faculty development and instructional skills remain a key factor in the implementation of this curriculum. From a facilitation perspective, the small-group structure is successful in that residents feel comfortable sharing vulnerable experiences with their peers. However, some limitations include challenges with encouraging all group members to share equally, as those with more intense emotions are often more outspoken, as well as challenges with providing residents with formal resources through which to report their challenging encounters to prompt institutional change (i.e., expressing frustrations with sessions during which they raise concerns but have no power to address underlying issues leading to the challenges they face). Moving forward, it would be beneficial to incorporate more feedback on the faculty experience to further optimize the structure and content of the sessions.

We acknowledge several other limitations. First, we implemented this curriculum with a subgroup of residents at two academic institutions. For future directions, we hope to expand to other institutions and residency training programs outside of internal medicine. Second, based on the Kirkpatrick model of evaluation,[19] our surveys were limited to reaction and self-reported learning. There was no direct assessment of participant ability to meet the intended objectives, and we did not use a validated survey instrument for our quantitative, Likert-scale questions. This limitation came from the variability and inconsistency with which NM curricula have been evaluated. Strength of results came from qualitative data, where open-ended response questions allowed for identification of emerging themes related to the course objectives. Because of this, we believe an enhanced qualitative analysis—in the form of semi-structured interviews or focus groups—can provide more direct measures of learning and behavior. With learning, interviews can explore the degree to which participants acquire reflective skills, sense of camaraderie, positive work-related attitudes, and comfort with analyzing literary text. With behaviors, interviews can explore how and why the sessions impact patient care or peer connectivity. Semi-structured interviews can also more directly target themes as they relate to validated survey measures of topics like professional identity formation, burnout, and resilience. Therefore, in the future, we hope to implement semi-structured interviews and focus groups with participants at the end of each academic year for theory-guided content analysis. Furthermore, future directions should continue to incorporate resident feedback from previous sessions and adapt themes around timely events and uniquely challenging experiences in the context of residency training. Finally, only one iteration of this educational activity was implemented and evaluated. We anticipate strength in future iterations as we continue to reassess and incorporate feedback.

In general, we found these sessions were well received by residents while promoting growth, positivity, and professional identity development during a particularly vulnerable period in a physician's career. We encourage outside institutions to adopt a similar model and invite programs to reach out to collaborate and review effective strategies to do so.

## Appendices

A. Facilitator Guide.docx
B. Burnout and Moral Injury.pptx
C. Compassion Fatigue.pptx
D. Working Through a Pandemic.pptx
E. Difficult Patient.pptx
F. The New Normal.pptx
G. Finding Meaning.pptx
H. Unpublished Narratives.docx
I. Survey.docx

*All appendices are peer reviewed as integral parts of the Original Publication.*

Copyright © 2024 Silver and Hussain This is an open-access publication distributed under the terms of the Creative Commons Attribution license.

**Michelle Silver, MD:** Instructor of Medicine, Department of Medicine, Harvard Medical School and Beth Israel Deaconess Medical Center; ORCID: https://orcid.org/0000-0001-6027-8782

**Farah Hussain, MD:** Assistant Professor of Clinical Medicine, Department of Medicine, Hospital of the University of Pennsylvania

**Disclosures**
None to report.

**Funding/Support**
None to report.

**Prior Presentations**
Silver M, Hussain F. Integration of a narrative medicine curriculum in primary care residency improves personal and professional identity development. Presented at: Society of General Internal Medicine (SGIM) Annual Meeting; April 6-9, 2022; Orlando, FL.

Silver M, Hussain F. Integration of narrative medicine curricula in primary care residency provides needed space for group reflection and connectedness. Poster presented virtually at: Northeast Group on Educational Affairs (NEGEA) Spring Meeting; April 20-22, 2021.

**Ethical Approval**
The Hospital of the University of Pennsylvania and the Beth Israel Deaconess Medical Center Institutional Review Boards deemed further review of this project not necessary.

## References

1. Wald HS, Anthony D, Hutchinson TA, Liben S, Smilovitch M, Donato AA. Professional identity formation in medical education for humanistic, resilient physicians: pedagogic strategies for bridging theory to practice. *Acad Med*. 2015;90(6):753-760. https://doi.org/10.1097/ACM.0000000000000725
2. Shanafelt TD, Hasan O, Dyrbye LN, et al. Changes in burnout and satisfaction with work-life balance in physicians and the general US working population between 2011 and 2014. *Mayo Clin Proc*. 2015;90(12):1600-1613. https://doi.org/10.1016/j.mayocp.2015.08.023
3. Miller E, Balmer D, Hermann N, Graham G, Charon R. Sounding narrative medicine: studying students' professional identity development at Columbia University College of Physicians and Surgeons. *Acad Med*. 2014;89(2):335-342. https://doi.org/10.1097/ACM.0000000000000098
4. Aluri J, Ker J, Marr B, et al. The role of arts-based curricula in professional identity formation: results of a qualitative analysis of learner's written reflections. *Med Educ Online*. 2023;28(1): 2145105. https://doi.org/10.1080/10872981.2022.2145105
5. Jones AH. Why teach literature in medicine? Answers from three decades. *J Med Humanit*. 2013;34(4):415-428. https://doi.org/10.1007/s10912-013-9241-9
6. Remein CD, Childs E, Pasco JC, et al. Content and outcomes of narrative medicine programmes: a systematic review of the literature through 2019. *BMJ Open*. 2020;10:e031568. https://doi.org/10.1136/bmjopen-2019-031568
7. Charon R, DasGupta S, Hermann N, et al. *The Principles and Practice of Narrative Medicine*. Oxford University Press; 2017.
8. Moniz T, Golafshani M, Gaspar CM, et al. How are the arts and humanities used in medical education? Results of a scoping review. *Acad Med*. 2021;96(8):1213-1222. https://doi.org/10.1097/ACM.0000000000004118
9. Shalaby R, Oluwasina F, Eboreime E, et al. Burnout among residents: prevalence and predictors of depersonalization, emotional exhaustion and professional unfulfillment among resident doctors in Canada. *Int J Environ Res Public Health*. 2023;20(4):3677. https://doi.org/10.3390/ijerph20043677
10. Neumann M, Edelhäuser F, Tauschel D, et al. Empathy decline and its reasons: a systematic review of studies with medical students and residents. *Acad Med*. 2011;86(8):996-1009. https://doi.org/10.1097/ACM.0b013e318221e615
11. Bajaj N, Phelan J, McConnell EE, Reed SM. A narrative medicine intervention in pediatric residents led to sustained improvements in resident well-being. *Ann Med*. 2023;55(1):849-859. https://doi.org/10.1080/07853890.2023.2185674
12. Kirkland KB, Craig SR. Exploring the surgical gaze through literature and art. *JAMA*. 2018;319(15):1532-1534. https://doi.org/10.1001/jama.2018.0396
13. Ronan LK, Czerwiec MK. A novel graphic medicine curriculum for resident physicians: boosting empathy and communication through comics. *J Med Humanit*. 2020;41(4):573-578. https://doi.org/10.1007/s10912-020-09654-2
14. Collier K, Gupta A, Vinson A. Motivating change in resident language use through narrative medicine workshops. *BMC Med Educ*. 2022;22:663. https://doi.org/10.1186/s12909-022-03721-z
15. Wesley T, Hamer D, Karam G. Implementing a narrative medicine curriculum during the internship year: an internal medicine residency program experience. *Perm J*. 2018;22(2):17-187. https://doi.org/10.7812/TPP/17-187
16. Clark MC, Rossiter M. Narrative learning in adulthood. *New Dir Adult Contin Educ*. 2008;(19):61-70. https://doi.org/10.1002/ace.306
17. Millota MM, van Thiel GJMW, van Delden JJM. Narrative medicine as a medical educational tool: a systematic review. *Med Teach*. 2019;41(7):802-810. https://doi.org/10.1080/0142159X.2019.1584274
18. Winkel AF. Narrative medicine: a writing workshop curriculum for residents. *MedEdPORTAL*. 2016;12:10493. https://doi.org/10.15766/mep_2374-8265.10493
19. Kirkpatrick DL. Techniques for evaluating training programs. *J Am Soc Train Dir*. 1959;13(12):21-26.

**Received:** December 16, 2023
**Accepted:** May 9, 2024
**Published:** October 22, 2024

Copyright © 2024 Silver and Hussain This is an open-access publication distributed under the terms of the Creative Commons Attribution license.
